# Effects of N-methyl-D-aspartate receptor knockdown and hypoxia/reoxygenation injury on the neuronal proteome and transcriptome

**DOI:** 10.3389/fnmol.2022.1004375

**Published:** 2022-12-15

**Authors:** Jinting He, Kaili Chen, Yujie Sui, Qiwei Yang

**Affiliations:** ^1^Department of Neurology, China-Japan Union Hospital of Jilin University, Changchun, China; ^2^Jilin Provincial Key Laboratory on Molecular and Chemical Genetic, Medical Research Center, Second Hospital of Jilin University, Changchun, Jilin, China

**Keywords:** hypoxia/reoxygenation neuronal injury, N-methyl-D-aspartate receptor, proteome, transcriptome, long non-coding RNA

## Abstract

**Introduction:**

Brain tissue is extremely sensitive to hypoxia/reoxygenation (H/R) injury, which can easily cause irreversible damage to neurons. H/R injury can induce neuronal apoptosis through glutamate-mediated excitotoxicity. N-methyl-d-aspartate receptor (NMDAR) is one of the main receptors of excitatory glutamate, and blocking NMDAR protects brain tissue from ischemic and hypoxic injury. However, NMDAR hypofunction can also cause psychotic symptoms or cognitive impairment. There is still a lack of systematic research on the changes in the proteome and transcriptome in neuronal cells under conditions of NMDAR hypofunction and H/R injury.

**Methods:**

We compared the changes in the proteome, transcriptome and lncRNA expression levels in neurons after NMDAR knockdown and H/R by isobaric tags for relative and absolute quantitation (iTRAQ) and RNA sequencing (RNA-Seq).

**Results:**

The results showed that the proteins Rps9, Rpl18 and Rpl15 and the lncRNAs XLOC_161072 and XLOC_065271 were significantly downregulated after NMDAR knockdown but upregulated after H/R; in contrast, the mRNAs Bank1 and Pcp4l1 and the lncRNAs XLOC_159404 and XLOC_031922 were significantly upregulated after NMDAR knockdown but downregulated after H/R.

**Discussion:**

In this study, we demonstrated the characterization of protein, mRNA, and lncRNA expression profiles in neurons following NMDAR knockdown and H/R injury. These molecules are involved in multiple biological functions and signaling pathways, and their roles in neurons lacking NMDAR and subjected to H/R injury deserve further study. Additionally, we found that lncRNAs respond fastest to hypoxic stimulation and that Gapdh is not suitable as a reference protein for NMDAR-reduced neuron-related experiments.

## Introduction

Brain tissue has high metabolic levels and is sensitive to ischemia and hypoxia. Hyperoxia/reoxygenation (H/R) in brain tissue easily causes irreversible damage to neurons. A large amount of evidence shows that a variety of hypoxic–ischemic brain diseases could trigger the excitotoxic effects of overactivation of receptors by excitatory amino acids, which eventually leads to neural injury (Lipton and Rosenberg, 1994; Shibasaki et al., 2018).

During cerebral hypoxia, the oxygen required by neurons to maintain ion homeostasis is exhausted. This destroys the ion gradient and depolarizes the membrane, resulting in the release of the excitatory neurotransmitter glutamate into the synaptic space (Calabresi et al., 1995; Pamenter et al., 2011; Borisova et al., 2018). In addition, energy consumption damages the function of the reuptake transporter, making it unable to remove excess glutamate, which results in accumulation of excitatory glutamate outside cells and overactivation of glutamate receptors (Johnston, 2005; Pregnolato et al., 2019).

N-methyl-D-aspartate receptor (NMDAR), one of the main subtypes of excitatory glutamate receptors, has ligand and voltage double-gated properties and high permeability to calcium ions (Haque et al., 2021). Different temporal and spatial expression levels of NMDARs control the generation and maturation of synapses and mediate synaptic transmission and plasticity (Gambrill and Barria, 2011; Johansson et al., 2020).

NMDAR is associated with many neurological and psychiatric disorders. Excessive or persistent activation of NMDAR, such as during traumatic brain injury or stroke, leads to excessive increases in intracellular calcium, which produces a delayed form of neuronal damage, resulting in neuronal excitotoxic death (Parsons and Raymond, 2014). Excitotoxicity induced by NMDAR has also been suggested as a potential mechanism of neurodegeneration in diseases such as Parkinsonism (Wang et al., 2020), Alzheimerism (Wang and Reddy, 2017), and Huntington’s disease (Kornhuber and Weller, 1997; Fan and Raymond, 2007). Activation of NMDAR is also associated with neurological sequelae after hypoxic injury (Chen et al., 2006; Ji et al., 2021). Therefore, NMDAR is a molecular target with strong therapeutic significance (Seillier et al., 2022). In previous research, NMDAR specific antagonists could significantly inhibit neuronal apoptosis in ischemic brain injury models (Mishra et al., 2011; Yu et al., 2015). Therefore, the evidence suggests that blockade of NMDAR protects against ischemic brain injury.

However, NMDAR hypofunction is also detrimental. Subanaesthetic doses of NMDAR pore blockers (such as ketamine and phencyclidine) in healthy volunteers can cause cognitive impairment and schizophrenia-like symptoms (Moghaddam and Javitt, 2012; Nakazawa and Sapkota, 2020). Similarly, anti-NMDAR encephalitis, which is characterized by reduced NMDAR expression and function, induces psychosis, abnormal behavior, and cognitive impairment (Seery et al., 2022). In addition, NMDAR antagonists or reduced NMDAR activity have been found to induce psychotic-like behavioral symptoms in various animal models (Jones et al., 2011). Decreased NMDAR function is a key feature of age-related cognitive deficits and may also occur in neurodegenerative disorders such as Alzheimer’s disease (Geoffroy et al., 2022).

However, under conditions of NMDAR knockdown or NMDAR knockdown with H/R injury, the intracellular proteomes and transcriptomes of neurons have not been systematically investigated. In this study, we compared the intracellular proteomes and transcriptomes of neurons under NMDAR knockdown and H/R injury conditions through isobaric tags for relative and absolute quantitation (iTRAQ) and RNA sequencing (RNA-Seq) in order to explore the effects of NMDAR and H/R on neurons.

## Materials and methods

### Neuronal cell culture and H/R treatment

The mouse hippocampal neuron HT22 cell line was provided by Procell Life Science & Technology (China). The cells were cultured in high-glucose DMEM (HyClone, United States) containing 10% FBS (Gibco, United States) and 1% penicillin–streptomycin (HyClone, United States) at 37°C in a humidified environment containing 5% carbon dioxide and 95% air. The medium was changed every 2–3 days, and when the cell density reached ~80%, passage was carried out at a ratio of 1:3–1:4.

For H/R of HT22 cells, the cells were cultured under hypoxic conditions of 1% oxygen, 5% carbon dioxide, and 94% nitrogen; the other culture conditions remained unchanged. Normal conditions were resumed after 2 h.

### Knockdown of NMDAR using small interfering RNA

SiRNA targeting the NMDAR [Mus musculus glutamate receptor, ionotropic, NMDA1 (zeta 1) (Grin1), transcript variant 1, mRNA] was designed by DSIR website.^1^ The siRNA sequences scoring top 2 were chosen and synthesized by GeneCreate Bioengineering Co., Ltd. (Wuhan, China). The specific sequences were CCAUGCACCUGCUGACAUUTT (si-NMDAR-1) and GCAGUAAACCAGGCCAAUATT (si-NMDAR-2). HT22 cells were seeded in a 6-well plate at a density of 1 × 10^5^ cells per well. When the cell density reached 70%, Lipofectamine 2000 (Invitrogen, United States) was used for transfection according to the instructions. Two micrograms of siRNA was used in each well for the experimental group, and an equal volume of PBS was used for the control group. The medium was changed to conventional medium 6 h after transfection.

### Real-time quantitative PCR detection

Total RNA was extracted using TRIzol Reagent (Ambion, United States), and reverse transcription was performed using a ReverTra Ace qPCR RT Kit (Toyobo, Japan) according to the manual. The primers used for RT-qPCR were designed and synthesized by GeneCreate Bioengineering Co., Ltd. (Wuhan, China), and the specific sequences were as follows: NMDAR-forward: GGTGGCTGGAGGCATCGTAG, NMDAR-reverse: GGCATCCTTGTGTCGCTTGTAG, Gapdh-forward: AGGTTGTCTCCTGCGACTTCA, Gapdh-reverse: TGGTCCAGGGTTTCTTACTCC. RT-qPCR was performed according to the manual of SYBR GREEN Realtime PCR Master Mix (Toyobo, Japan). The reaction conditions were predenaturation at 95°C for 1 min; 40 cycles of denaturation at 95°C for 15 s and annealing and extension at 60°C for 30 s; and melting curve detection.

### Western blot detection

Total protein was extracted using RIPA lysis buffer (Beyotime, China) containing protease inhibitor cocktail (Beyotime, China) according to the manual. Fifty five micrograms of total protein from each sample were used for SDS-PAGE and then transferred to PVDF membranes (Millipore, United States). The antibodies used were anti-NMDAR (1:1,000, A01808, BOSTER, United States) and anti-β-actin (1:2,000, 60008-1-Ig, Proteintech, China). A luminescent and fluorescent biological image analysis system (Furi Science & Technology, China) was used to detect exposure after adding the enhanced chemiluminescent (ECL) reagent.

### Cell counting kit-8 detection of cell proliferation ability

Cells were seeded into 96-well plates at a density of 1,000 cells per well. Then, 100 μl of cell culture medium was added to each well, and the cells were cultured in a 37°C, 5% carbon dioxide incubator. For detection, 10 μl of CCK-8 solution (Solarbio Science & Technology, China) was added to each well; the same volumes of cell culture medium and CCK-8 solution were added to blank control wells, but no cells were seeded. After incubation for 30 min, the absorbance was measured at 450 nm with a microplate reader.

### Fluorescence staining detection of calcium ion content

Cells were seeded in thin-bottom dishes at a density of 1 × 10^4^ cells per dish. After fixation in 4% paraformaldehyde for 10–15 min, the cells were washed three times with PBS, stained according to the instructions of a Fluo-4 AM kit (Beyotime Biotechnology, China), and incubated at 37°C for 30 min for fluorescent probe loading. Subsequently, the cells were washed three times with PBS. After further incubation for 30 min, the nuclei were stained with DAPI (Solarbio Science & Technology, China). An anti-fluorescence quencher was added dropwise. Photographs were taken with a laser confocal microscope. ImageJ was employed to analyze and measure the mean fluorescence intensity.

### iTRAQ detection of proteomes

Cells were collected with a cell scraper, and then protein lysis buffer (7 M urea + 2 M thiourea + 4% SDS + 40 mM Tris–HCl, pH 8.5 + 1 mM PMSF + 2 mM EDTA) was added and mixed. The cells were incubated on ice for 5 min. DTT with a final concentration of 10 mM was added, and the cells were sonicated in an ice bath for 15 min before being centrifuged at 13,000 × *g* for 20 min at 4°C. The supernatant was collected, four times the volume of precooled acetone was added, and the mixture was allowed to stand at −20°C overnight. The protein pellet was collected by centrifugation and air-dried. Add 8 M urea/100 mM Tetraethylammonium bromide (TEAB) (pH 8.0) solution to redissolve the protein, and DTT was added to a final concentration of 10 mM. The reduction reaction was conducted in a water bath at 56°C for 30 min. Subsequently, iodoacetamide was added to a final concentration of 55 mM, and the alkylation reaction was carried out at room temperature in the dark for 30 min. Next, 100 μg of protein was diluted five times with 100 mM TEAB, 2 μg of trypsin was added, and the proteins were digested at 37°C overnight. The enzymatically hydrolyzed peptides were desalted with a C18 chromatographic column, and then the desalted peptides were vacuum freeze-dried.

Peptides were dissolved in 0.5 M TEAB. The samples were labeled and mixed according to the instructions of the iTRAQ labeling kit (SCIEX, United States). The pooled peptides were then fractionated using an Ultimate 3000 HPLC system (Thermo DINOEX, United States) with a Durashell C18 column (5 μm, 100 Å, 4.6 × 250 mm). A flow rate of 1 ml/min was used, and one tube was collected every minute. A total of 42 secondary fractions were collected and combined into 15 fractions. The combined fractions were desalted on a Strata-X column and dried *in vacuo*.

Peptide samples were dissolved in 2% acetonitrile +0.1% formic acid and analyzed using a TripleTOF 5600+ mass spectrometer (SCIEX, United States) coupled to an Eksigent nano LC system (SCIEX, United States). The peptide solution was applied to a C18 capture column (5 μm, 100 μm × 20 mm), and then gradient elution was performed on a C18 analytical column (3 μm, 75 μm × 150 mm) with a gradient time of 90 min and a flow rate of 300 nl/min. The two mobile phases were 2% acetonitrile +0.1% formic acid +98% H_2_O and 98% acetonitrile +0.1% formic acid +2% H_2_O. For data-dependent acquisition, primary mass spectra were scanned with an ion accumulation time of 250 ms, and secondary mass spectra of 30 precursor ions were acquired with an ion accumulation time of 50 ms. MS1 spectra were collected in the range of 350–1,500 m/z, and MS2 spectra were collected in the range of 100–1,500 m/z. The precursor ion dynamic exclusion time was set to 15 s. The detection results were identified and annotated with ProteinPilot v4.5.

### RNA-Seq detection of the mRNA and lncRNA transcriptome

RNA-Seq detection was performed by GeneCreate Bioengineering Co., Ltd. (Wuhan, China). Briefly, ribosomal RNA was removed from the total RNA. The RNA was then broken down into short fragments of 250–300 bp using the enzyme RNase R. The fragmented RNA was used as a template, and random oligonucleotides were used as primers to synthesize the first strand of cDNA. Subsequently, the RNA strand was degraded by RNase H, and the second strand of cDNA was synthesized from dNTPs under the DNA polymerase I system. The purified double-stranded cDNA was end-repaired, A-tailed, and ligated with sequencing adapters. AMPure XP beads (Beckman Coulter, United States) were used to screen cDNAs of ~350–400 bp. The second strand of U-containing cDNA was degraded by the USER enzyme. Finally, PCR amplification was performed to obtain cDNA libraries.

The libraries were pooled after quantification and subjected to Illumina PE150 sequencing. Illumina Casava 1.8 was used for quality control and annotation of sequencing results, and then the software programs TopHat2,^2^ HISAT2,^3^ and STAR^4^ were used for alignment analysis of the sequencing data.

### Statistical analysis

At least 3 biological replicates were performed for each group of experiments. A *t*-test was used to compare measurement data between two groups, and ANOVA was used to compare multiple groups. The expression values of the samples were clustered using a hierarchical clustering method. *p* < 0.05 was defined as the criterion for a significant difference. Differential expression analysis was performed using edgeR, DESeq2, and DEGSeq software. GOseq software was used for Gene Ontology (GO) enrichment analysis, and KOBAS (2.0) was used for Kyoto Encyclopedia of Genes and Genomes (KEGG) pathway enrichment analysis.

## Results

### Knockdown efficiency of NMDAR siRNA

**Figure 1:**
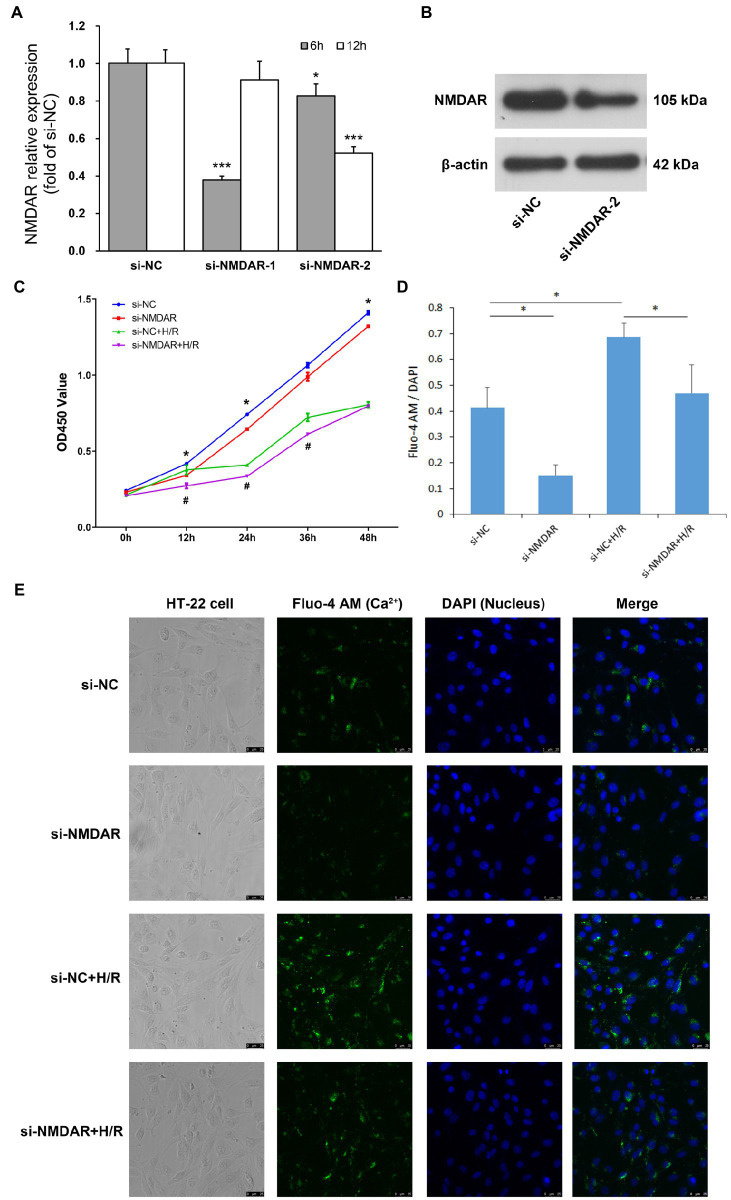
**(A)** NMDAR mRNA relative expression level. *T*-test was used to compare data between: ① si-NC and si-NMDAR-1 (6 h), ② si-NC and si-NMDAR-1 (12 h), ③ si-NC and si-NMDAR-2 (6 h), ④ si-NC and si-NMDAR-2 (12 h). * represents *p* < 0.05; *** represents *p* < 0.001. **(B)** NMDAR protein western blot detection. **(C)** HT22 cell proliferation curve. *T*-test was used to compare data between: ① si-NC and si-NMDAR at each time point, ② si-NC + H/R and si-NMDAR+H/R at each time point. * represents *p* < 0.05 between si-NC and si-NMDAR; # represents *p* < 0.05 between si-NC + H/R and si-NMDAR+H/R. **(D)** Mean fluorescence intensity of calcium staining. *T*-test was used to compare data between: ① si-NC and si-NMDAR, ② si-NC and si-NC + H/R, ③ si-NC + H/R and si-NMDAR+H/R. * represents *p* < 0.05. **(E)** Calcium staining micrographs of HT22 cells.

We designed two siRNAs against NMDAR, transfected them into HT22 cells, and detected the expression levels of NMDAR by RT-qPCR at 6 h and 12 h after transfection. The results showed that si-NMDAR-1 significantly knocked down the expression of NMDAR at 6 h after transfection but that the expression level rebounded after 12 h; in contrast, si-NMDAR-2 significantly knocked down the expression of NMDAR at both 6 and 12 h after transfection, with knockdown efficiencies of 82.56% and 52.11%, respectively (Figure 1A). This result was also confirmed by the western blot results of the protein 12 h after the si-NMDAR-2 knockdown (Figure 1B). Therefore, we selected si-NMDAR-2 (hereafter referred to as si-NMDAR) for subsequent knockdown experiments.

### After knockdown of NMDAR, the resistance of neurons to H/R injury was significantly reduced

To investigate the effect of NMDAR on the sensitivity of neuronal cells to H/R, we divided HT22 cells into four groups: the si-NC group was the control group, the si-NMDAR group was treated with siRNA to knockdown the expression level of NMDAR, the si-NC + H/R group was subjected to H/R but without NMDAR knockdown, and the si-NMDAR+H/R group was subjected to H/R after knockdown of NMDAR by siRNA. After the cells were transfected with siRNA, they were subjected to hypoxic conditions immediately after replacement of the transfection medium with conventional medium, subjected to hypoxia for 2 h and then subjected to reoxygenation. CCK-8 assays were performed after 0, 12, 24, 36, and 48 h of reoxygenation. The results showed that after knockdown of NMDAR in HT22 cells, the proliferation ability of the cells was weakened, especially at 12 h after transfection. In addition, the effect of NMDAR knockdown on cell proliferation exceeded the effect of H/R. After H/R injury, the proliferation ability of HT22 cells was significantly weakened beginning at 12 h but rebounded after 24 h. Knockdown of NMDAR further aggravated the weakening of the proliferation ability caused by H/R injury (Figure 1C).

### The calcium ion transport ability of neuronal cells during H/R injury was significantly reduced after knockdown of NMDAR

To investigate the effect of NMDAR on the calcium absorption of neuronal cells, we divided HT22 cells into four groups as described above and performed calcium and DAPI fluorescence staining after 12 h of reoxygenation. The results showed that the uptake of calcium ions in HT22 cells was significantly reduced after knockdown of NMDAR but enhanced after H/R treatment; however, calcium ion uptake was also significantly reduced after knockdown of NMDAR followed by H/R treatment (Figures 1D,E).

### Effects of NMDAR on the neuronal proteome during H/R injury

To explore which proteins were affected by NMDAR knockdown and H/R in neuronal cells, we applied iTRAQ to detect the protein expression profiles in the si-NC (negative control) group, the si-NMDAR (siRNA-mediated NMDAR-knockdown) group, and the si-NMDAR+H/R (NMDAR-knockdown and H/R-exposed) group.

**Figure 2:**
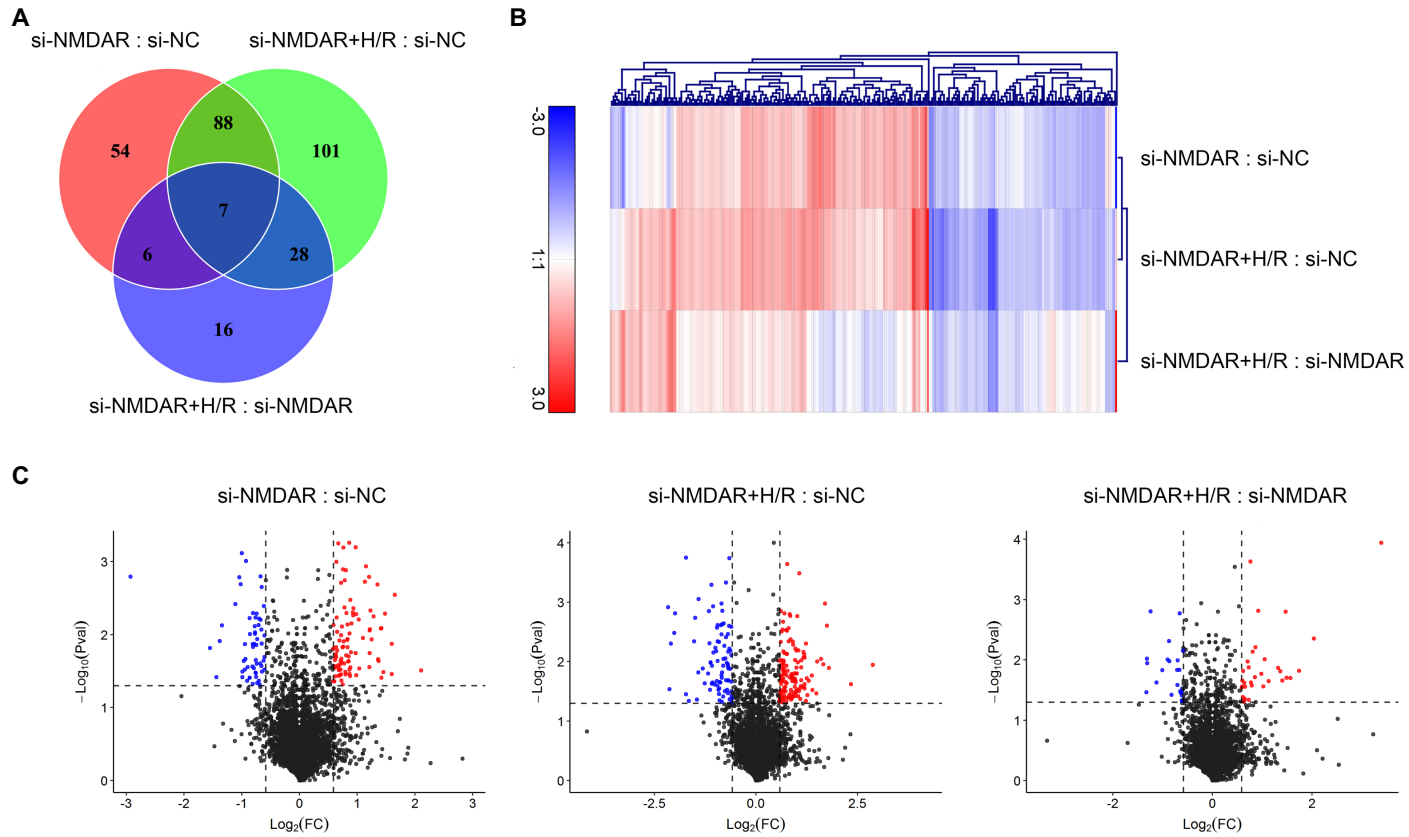
**(A)** Venn diagram showing the numbers of differentially expressed proteins common among comparisons and unique to each comparison. **(B)** Cluster analysis heatmap showing the differentially expressed proteins in each comparison. The vertical axis indicates the grouping, the horizontal axis indicates the differentially expressed proteins, the top indicates the clustering of proteins according to the degree of expression similarity, and the right side indicates the clustering of the samples according to the similarity degree of the expression profile. The expression level gradually increases as the color changes from blue to red. The data is the log_2_ logarithmic value of the ratio of protein abundance between pairs of comparison groups. **(C)** Volcano plots showing the distribution of differentially expressed proteins in each comparison. The horizontal axis indicates the fold change in differential expression, the vertical axis indicates the statistical significance of the differential expression. Value of *p* < 0.05 [−Log_10_(Pval) > 1.30], at the same time FC < −1.5 [Log_2_(FC) < −0.58] or FC > 1.5 [Log_2_(FC) > 0.58] were considered to be significantly different in expression level. Red dots represent significantly upregulated proteins, and the blue dots represent significantly downregulated proteins.

Three independent iTRAQ experiments were performed in each group. The numbers of proteins identified in three independent experiments were 4,706, 4,592, and 4,676 (when filtered by at least two unique peptides, 3,864, 3,759, and 3,848 proteins were identified, respectively). A total of 6,249 proteins were identified (union), of which 3,251 proteins (intersection) were simultaneously present in three independent experiments and quantified after combining biological replicates. In the relative quantification results, we found that there were significant differences in expression between the compared groups (si-NMDAR:si-NC, si-NMDAR+H/R:si-NC, si-NMDAR+H/R:si-NMDAR). When the fold change (FC) of the protein is >1.5 and the *p*-value is <0.05, the protein is regarded as differentially expressed between groups. There were 155, 224, and 57 differentially expressed proteins, respectively (Figure 2A). Table 1 lists the top 10 up-/downregulated proteins in each comparison. A cluster analysis heatmap and volcano plot are shown in Figures 2B,C, and the detailed cluster analysis heatmap is shown in Supplementary material 1.

**Table 1:** The top 10 proteins up-/downregulated in each comparison group.

Group	Gene name	State	Log_2_Fold change	Value of *p*
si-NMDAR: si-NC	Tfrc	Up	2.108357	0.031
	Ddx5	Up	1.65306	0.003
	Canx	Up	1.60027	0.035
	Hist2h2aa1	Up	1.596458	0.013
	Shmt2	Up	1.482848	0.005
	Lrpprc	Up	1.451013	0.033
	Plec	Up	1.420078	0.008
	Slc25a5	Up	1.419539	0.039
	Pdia3	Up	1.411426	0.008
	Cs	Up	1.370164	0.023
	Acy1	Down	−0.9885	0.038
	Ehd2	Down	−0.99712	0.001
	Gpi	Down	−1.01742	0.002
	Gapdh	Down	−1.04097	0.002
	Rpl15	Down	−1.11092	0.004
	Prdx1	Down	−1.34008	0.007
	Lrrc71	Down	−1.38082	0.012
	Cavin1	Down	−1.43831	0.038
	Rpl18	Down	−1.54793	0.015
	Rps9	Down	−2.92139	0.002
si-NMDAR+H/R: si-NC	Rpl6	Up	2.882252	0.012
	Tfrc	Up	2.347382	0.024
	Mybbp1a	Up	1.798673	0.013
	Ddx5	Up	1.750607	0.002
	Anxa2	Up	1.703101	0.001
	Lrrc59	Up	1.664483	0.011
	H1f5	Up	1.606442	0.009
	Shmt2	Up	1.508429	0.010
	Slc25a5	Up	1.49211	0.034
	H1-4	Up	1.391218	0.030
	S100a4	Down	−1.52699	0.005
	Lrrc71	Down	−1.52699	<0.001
	Nasp	Down	−1.65745	0.0458
	Lgals3	Down	−1.72261	0.0355
	Pgk1	Down	−1.72738	0.0002
	Prdx1	Down	−1.99424	0.0015
	Aldoa	Down	−2.01742	0.0033
	EG433182	Down	−2.09542	0.0050
	S100a6	Down	−2.12658	0.0290
	Tpi1	Down	−2.16488	0.0012
si-NMDAR+H/R: si-NMDAR	Rps9	Up	3.393691	<0.001
	Rpl6	Up	2.037031	0.004
	H1-4	Up	1.74373	0.015
	Rpl18	Up	1.569005	0.020
	H1f0	Up	1.49057	0.020
	Rpl14	Up	1.473008	0.002
	Rps8	Up	1.40163	0.022
	Rpl15	Up	1.36233	0.016
	H1f5	Up	1.31904	0.014
	Rpl24	Up	1.130272	0.022
	Rps29	Down	−0.81858	0.038
	Pgk1	Down	−0.8625	0.010
	Hmgb1	Down	−0.87039	0.005
	Txn	Down	−0.89701	0.010
	Cfl1	Down	−1.00289	0.015
	Tpi1	Down	−1.12658	0.024
	Aldoa	Down	−1.24127	0.002
	EG433182	Down	−1.30401	0.011
	S100a4	Down	−1.31115	0.010
	Lgals3	Down	−1.32554	0.034

The differentially expressed proteins were functionally annotated by GO analysis. To determine the functions of the differentially expressed proteins more clearly, we performed independent functional annotation of the up-and downregulated differentially expressed proteins. The results showed large differences in GO functional classifications between the up-and downregulated differentially expressed proteins. For example, GO functions such as “virion” and “protein binding transcription factor activity” were associated with the upregulated proteins but not with the downregulated proteins. In addition, there were clear differences in the degrees of functional concentration (Supplementary material 2).

Furthermore, we performed GO enrichment analysis on the differentially expressed proteins in each comparison. The GO functional enrichment analysis revealed GO functional terms that were significantly enriched for the differentially expressed proteins compared to all identified proteins and identifies which biological functions are associated with the differentially expressed proteins. The top 20 terms from the significant enrichment analysis in each category (biological process, cellular component, and molecular function) are shown in Supplementary material 3. The results showed that the most significantly enriched terms differed among the comparisons. For example, in the biological process category, the most enriched term in the si-NMDAR:si-NC comparison was “response to stimulus,” followed by “response to chemical stimulus”; the most enriched term in the si-NMDAR+H/R:si-NC comparison was “multicellular organismal process,” followed by “response to chemical stimulus”; and the most enriched term in the si-NMDAR+H/R:si-NMDAR comparison was “reproductive process,” followed by “protein target” (Supplementary material 3).

We also performed KEGG functional annotation on the signaling pathways related to these differentially expressed proteins. To determine the most important biochemical metabolic pathways and signal transduction pathways associated with the proteins, we performed a proportional analysis of the top 10 related pathways according to the number of differentially expressed proteins. However, there were significant changes in “metabolic pathways” in all comparisons, and except for the upregulated proteins in the si-NMDAR+HR:si-NMDAR group, there were changes in the “microbial metabolism in diverse environments” pathways in the other groups. Similarly, except for the downregulated proteins in the si-NMDAR+HR:si-NC group, there were changes in the “ribosome” pathway in the other groups (Supplementary material 4). Furthermore, we performed pathway enrichment analysis on the differentially expressed proteins and determined the top 20 pathways for each comparison. The results showed that “ribosome,” “base excision repair,” “drug metabolism-cytochrome p450,” “glycolysis/gluconeogenesis,” “metabolism of xenobiotics by cytochrome p450,” “microbial metabolism in diverse environments,” and “tyrosine metabolism” were associated with all three comparisons (Supplementary material 4).

Based on the above results, we found that the proteins Rps9, Rpl18, and Rpl15 showed completely opposite expression characteristics in the si-NMDAR:si-NC comparison and the si-NMDAR+H/R:si-NMDAR comparison. These expression of these proteins was significantly downregulated in the si-NMDAR:si-NC comparison and significantly upregulated in the si-NMDAR+H/R:si-NMDAR comparison. The proteins all belong to the ribosomal protein family, their functions are essentially the same according to GO enrichment analysis, and they were all located in the “ribosome” KEGG pathway (Supplementary material 5).

### Effects of NMDAR on the neuronal mRNA transcriptome during H/R injury

**Figure 3:**
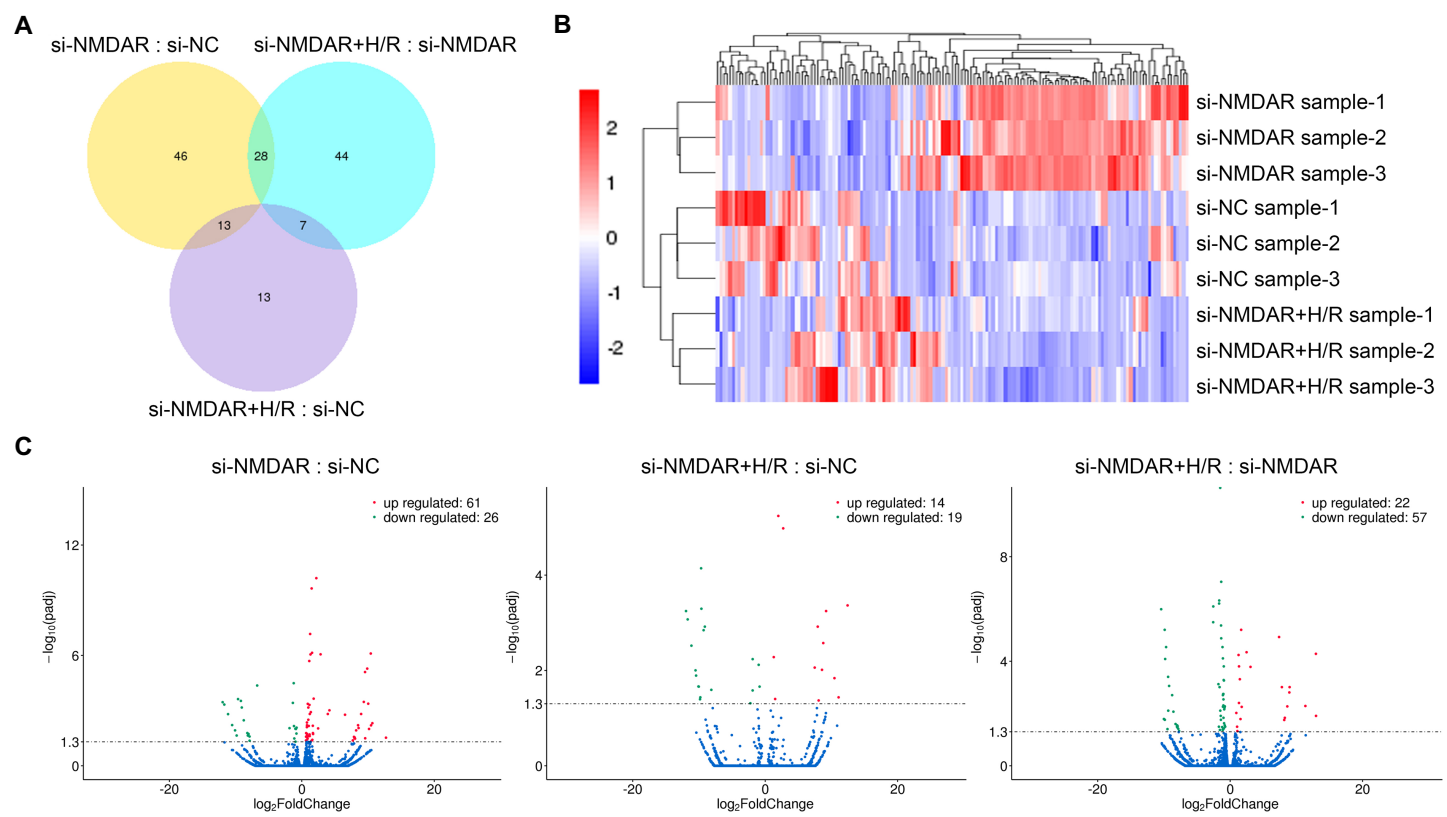
**(A)** Venn diagram showing the numbers of differentially expressed mRNAs common among comparisons and unique to each comparison. **(B)** Cluster analysis heatmap showing the differentially expressed mRNAs in each comparison. The vertical axis indicates the samples, the horizontal axis indicates the differentially expressed mRNAs, the top indicates the clustering of mRNAs according to the degree of expression similarity, and the right side indicates the clustering of the samples according to the similarity degree of the expression profile. The expression level gradually increases as the color changes from blue to red. The data is the log_2_ logarithmic value of the ratio of protein abundance between pairs of comparison groups. **(C)** Volcano plots showing the distribution of differentially expressed mRNAs in each comparison. The horizontal axis indicates the fold change in differential expression, the vertical axis indicates the statistical significance of the differential expression. *p*-adj < 0.05 [−Log_10_(Padj) > 1.30] was considered to be significantly different in expression level. Red dots represent significantly upregulated mRNAs, and the green dots represent significantly downregulated mRNAs.

To further explore the changes in intracellular mRNA transcript levels after NMDAR knockdown and H/R exposure, we used RNA-Seq to detect the mRNA transcriptomes in the si-NC group, si-NMDAR group, and si-NMDAR+H/R group. When the adjusted *p*-value (*p*-adj) is <0.05, the mRNA is regarded as differentially expressed between groups. The relative quantification results showed that 87, 33, and 79 mRNAs were significantly differentially expressed in each comparison (Figure 3A). Table 2 lists the top 10 up-/downregulated mRNAs in each comparison. The cluster analysis heatmap and volcano plot are shown in Figures 3B,C, and the detailed cluster analysis heatmap is shown in Supplementary material 6.

**Table 2:** The top 10 mRNAs up-/downregulated in each comparison group.

Group	Gene name	State	Log_2_Fold change	*p*-adj
si-NMDAR: si-NC	Gm43738	Up	12.711	0.030
	Slc22a14	Up	11.752	<0.001
	AC125149.3	Up	10.656	0.005
	Astn2	Up	10.459	0.006
	Bank1	Up	10.414	<0.001
	Morn5	Up	10.154	0.010
	B4galnt2	Up	10.033	<0.001
	Pcp4l1	Up	9.881	<0.001
	Myrfl	Up	9.573	0.032
	Hist1h3e	Up	9.551	<0.001
	Yipf7	Down	−8.765	0.003
	Gm4907	Down	−9.064	0.001
	Fam184b	Down	−9.201	<0.001
	Col25a1	Down	−9.631	<0.001
	Gabrb1	Down	−9.838	0.023
	Csmd3	Down	−10.079	0.012
	Cacna2d3	Down	−10.519	0.006
	Lrrc69	Down	−11.136	0.002
	Gpc5	Down	−11.704	<0.001
	Gm14327	Down	−11.955	<0.001
si-NMDAR+H/R: si-NC	Astn2	Up	12.441	<0.001
	Gm49333	Up	11.083	0.037
	Gm44973	Up	10.460	0.015
	Hist1h3e	Up	10.207	<0.001
	Adtrp	Up	9.197	0.001
	Kif28	Up	8.769	0.003
	Adcy8	Up	8.576	0.010
	Tnfrsf17	Up	8.073	0.043
	S100a7a	Up	7.933	0.001
	Hist2h3c1	Up	7.475	0.009
	Tmprss11e	Down	−9.678	<0.001
	4930486L24Rik	Down	−9.793	0.037
	Gabrb1	Down	−9.841	0.040
	Ankfn1	Down	−10.030	0.022
	Csmd3	Down	−10.082	0.022
	L3mbtl4	Down	−10.434	0.013
	Cacna2d3	Down	−10.521	0.010
	Lrrc69	Down	−11.139	0.003
	Gpc5	Down	−11.707	0.001
	Gm14327	Down	−11.957	0.001
si-NMDAR+H/R: si-NMDAR	Gm49333	Up	12.966	0.012
	Gm15093	Up	12.950	<0.001
	Gcat	Up	11.360	0.005
	Sh2d4b	Up	8.980	0.001
	Cfap57	Up	8.940	0.002
	Mmrn2	Up	8.632	0.005
	Spink5	Up	8.257	0.015
	Gm1123	Up	8.168	0.018
	S100a7a	Up	7.811	0.001
	Hist2h3c2	Up	7.387	<0.001
	Cadm1	Down	−9.349	0.008
	Gm4724	Down	−9.369	<0.001
	Dync1i1	Down	−9.466	0.039
	Gm3488	Down	−9.671	<0.001
	Scn7a	Down	−9.813	<0.001
	Gbp8	Down	−9.863	0.017
	Pcp4l1	Down	−9.886	<0.001
	Magea5	Down	−9.987	0.016
	Trpc5	Down	−10.006	0.016
	Bank1	Down	−10.417	<0.001

Similar to the method for proteome analysis, the differentially expressed mRNAs in each comparison were further subjected to GO enrichment analysis. The results showed that the differentially expressed mRNAs had large differences in the most enriched terms (Supplementary material 7A–C). A directed acyclic graph (DAG) was drawn for the biological processes, cellular components, and molecular functions according to their potential regulatory relationships in order to reveal the potential functional connections of these differentially expressed mRNAs (Supplementary material 8). We also performed pathway enrichment analysis on the differentially expressed mRNAs to determine the top 20 related pathways for each comparison. Two pathways, “bladder cancer” and “intestinal immune network for IgA production,” were enriched in all three comparisons (Supplementary material 7D–F).

Based on the above results, we found that the two mRNAs Bank1 and Pcp4l1 showed completely opposite expression characteristics in the si-NMDAR:si-NC comparison and the si-NMDAR+H/R:si-NMDAR comparison: they were significantly upregulated in the si-NMDAR:si-NC comparison and significantly downregulated in the si-NMDAR+H/R:si-NMDAR comparison. GO enrichment analysis revealed that the mRNAs Bank1 and Pcp4l1 were both involved in “binding” and “protein binding” processes. However, unexpectedly, these two mRNAs were not enriched in the annotated pathways in this study.

### Effects of NMDAR on neuronal lncRNA expression during H/R injury

**Figure 4:**
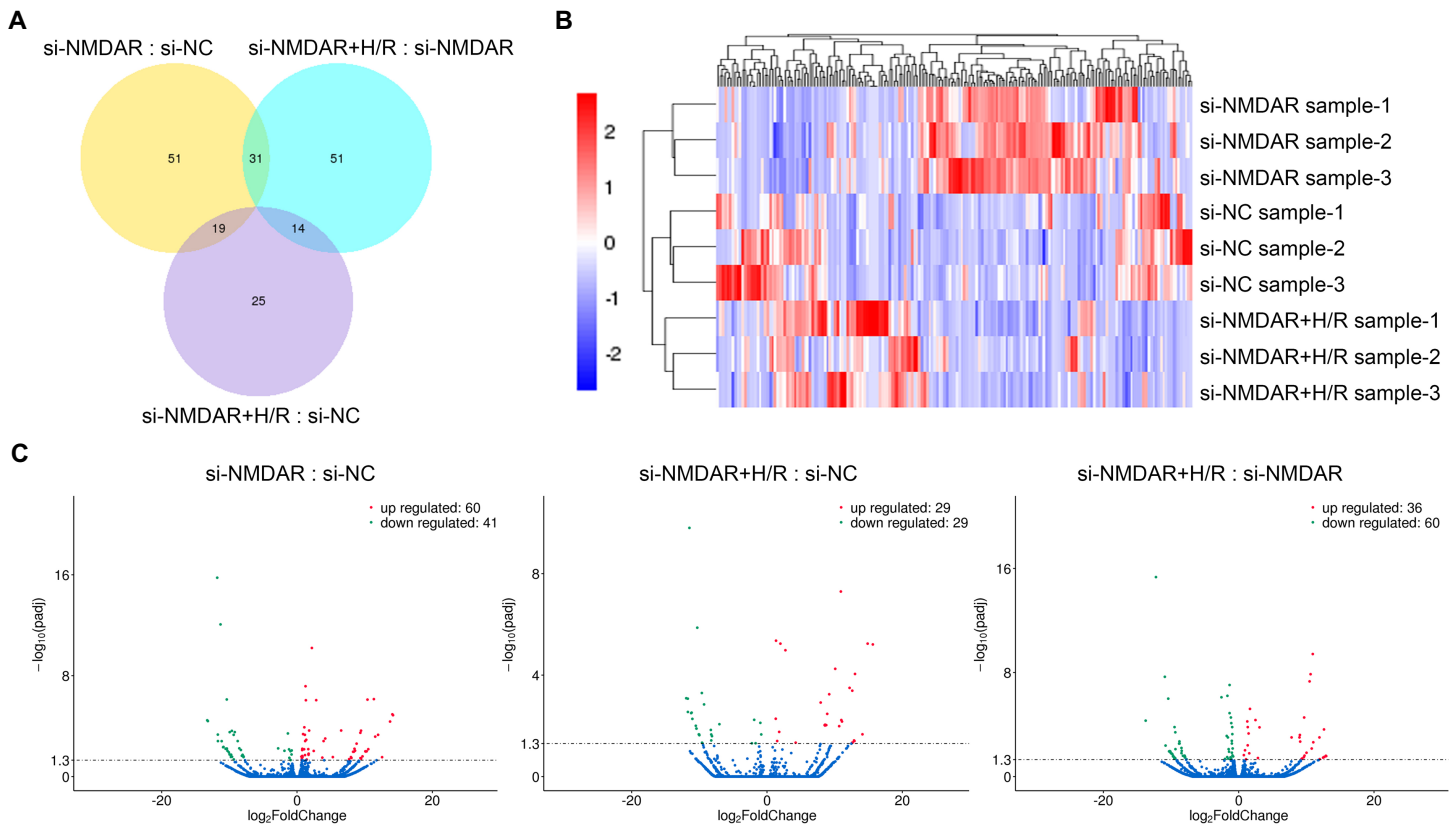
**(A)** Venn diagram showing the numbers of differentially expressed lncRNAs common among comparisons and unique to each comparison. **(B)** Cluster analysis heatmap showing the differentially expressed lncRNAs in each comparison. The vertical axis indicates the samples, the horizontal axis indicates the differentially expressed lncRNAs, the top indicates the clustering of lncRNAs according to the degree of expression similarity, and the right side indicates the clustering of the samples according to the similarity degree of the expression profile. The expression level gradually increases as the color changes from blue to red. The data is the log_2_ logarithmic value of the ratio of protein abundance between pairs of comparison groups. **(C)** Volcano plots showing the distribution of differentially expressed lncRNAs in each comparison. The horizontal axis indicates the fold change in differential expression, the vertical axis indicates the statistical significance of the differential expression. *p*-adj < 0.05 [−Log_10_(Padj) > 1.30] was considered to be significantly different in expression level. Red dots represent significantly upregulated lncRNAs, and the green dots represent significantly downregulated lncRNAs.

Similar to the method for mRNA transcriptome detection, we also detected the expression profiles of lncRNAs in the above comparisons to further investigate the changes in intracellular lncRNA expression levels after NMDAR knockdown and H/R exposure. When the *p*-adj is <0.05, the lncRNA is regarded as differentially expressed between groups. The relative quantification results showed that 101, 58, and 96 lncRNAs were significantly differentially expressed in each comparison (Figure 4A). Table 3 lists the top 10 up-/downregulated lncRNAs in each comparison. The cluster analysis heatmap and volcano plot are shown in Figures 4B,C, and the detailed cluster analysis heatmap is shown in Supplementary material 9.

**Table 3:** The top 10 lncRNAs up-/downregulated in each comparison group.

Group	lncRNA name	State	Log_2_Fold change	*p*-adj
si-NMDAR: si-NC	XLOC_029864	Up	14.191	<0.001
	Gm49936	Up	14.094	<0.001
	XLOC_159404	Up	13.752	<0.001
	XLOC_005151	Up	12.575	0.029
	XLOC_004709	Up	12.458	<0.001
	XLOC_155543	Up	11.985	<0.001
	XLOC_003058	Up	11.559	0.001
	Gm11508	Up	11.359	<0.001
	XLOC_031922	Up	10.865	<0.001
	Gm45193	Up	10.534	0.007
	XLOC_065271	Down	−10.559	0.006
	XLOC_157103	Down	−10.869	0.004
	XLOC_061664	Down	−10.925	0.005
	Lrrc69	Down	−11.136	0.002
	XLOC_078536	Down	−11.309	<0.001
	Gm33696	Down	−11.692	0.002
	Gpc5	Down	−11.704	<0.001
	4930401G09Rik	Down	−11.795	<0.001
	XLOC_029806	Down	−13.179	<0.001
	XLOC_161072	Down	−13.269	<0.001
si-NMDAR+H/R: si-NC	XLOC_029864	Up	15.644	<0.001
	XLOC_029876	Up	14.875	<0.001
	Gm49936	Up	14.110	0.022
	XLOC_003058	Up	13.008	<0.001
	XLOC_028364	Up	12.925	0.040
	XLOC_031910	Up	12.866	0.038
	XLOC_005156	Up	12.662	0.045
	Gm8739	Up	12.605	<0.001
	XLOC_111180	Up	12.597	0.046
	XLOC_004709	Up	12.205	<0.001
	L3mbtl4	Down	−10.434	0.013
	Cacna2d3	Down	−10.521	0.010
	XLOC_002463	Down	−11.030	0.005
	Lrrc69	Down	−11.139	0.003
	XLOC_161023	Down	−11.211	0.003
	XLOC_046129	Down	−11.469	<0.001
	Gm33696	Down	−11.694	0.003
	Gpc5	Down	−11.707	0.001
	XLOC_124858	Down	−11.807	<0.001
	Gm38048	Down	−11.939	0.001
si-NMDAR+H/R: si-NMDAR	XLOC_161072	Up	13.013	<0.001
	XLOC_028364	Up	12.935	0.027
	XLOC_031910	Up	12.886	0.025
	XLOC_005156	Up	12.672	0.031
	Gm8739	Up	12.609	<0.001
	XLOC_111180	Up	12.607	0.032
	XLOC_135584	Up	12.473	0.035
	XLOC_058931	Up	11.965	0.046
	Gm41496	Up	11.932	0.001
	XLOC_065271	Up	11.024	0.002
	8430426J06Rik	Down	−10.108	0.014
	XLOC_057817	Down	−10.165	0.012
	XLOC_026109	Down	−10.181	0.011
	Gm16685	Down	−10.295	0.012
	Bank1	Down	−10.417	<0.001
	XLOC_161023	Down	−10.815	0.004
	XLOC_127612	Down	−10.920	<0.001
	Gm38048	Down	−12.232	<0.001
	XLOC_031922	Down	−12.756	<0.001
	XLOC_159404	Down	−13.753	<0.001

On this basis, we further carried out GO enrichment analysis for the target genes of the differentially expressed lncRNAs in each comparison to elucidate the target genes that were coexpressed with the lncRNAs and the target genes that colocalized with the lncRNAs. Coexpression refers to a relationship between lncRNAs and detectable mRNAs indicated by a correlation of expression regulation; colocation refers to a relationship between lncRNAs and mRNAs indicated by the presence of lncRNAs within the upstream and downstream 100 kb range. The results showed that the target genes of the differentially expressed lncRNAs were significantly different in terms of enrichment, whether between comparisons or between the coexpression and colocation analyses within the same comparison (Supplementary material 10). A DAG was drawn for each GO process according to the potential regulatory relationships to reveal the potential functional connections of these differentially expressed lncRNAs (Supplementary material 11). We also performed pathway enrichment analysis on the target genes to determine the top 20 related pathways in each group. The results showed that the “HIF-1 signaling pathway” was involved in coexpression in all three comparisons, “mismatch repair” was involved in colocation in all three comparisons, and “mino sugar and nucleotide sugar metabolism” was involved in both coexpression and colocation in the si-NMDAR+H/R:si-NMDAR comparison (Supplementary material 12).

Based on the above results, we found that four lncRNAs, XLOC_159404, XLOC_031922, XLOC_161072, and XLOC_065271, showed completely opposite expression characteristics in the si-NMDAR:si-NC comparison and the si-NMDAR+H/R:si-NMDAR comparison. XLOC_159404 and XLOC_031922 were significantly upregulated in the si-NMDAR:si-NC comparison but downregulated in the si-NMDAR+H/R:si-NMDAR comparison; in contrast, XLOC_161072 and XLOC_065271 were significantly downregulated in the si-NMDAR:si-NC comparison but upregulated in the si-NMDAR+H/R:si-NMDAR comparison. Notably, Bank1, which was significantly differentially expressed at the mRNA level, also exhibited differences at the lncRNA level: it was upregulated in the si-NMDAR:si-NC comparison (ranked 11th) and downregulated in the si-NMDAR+H/R:si-NMDAR comparison (ranked 6th). The target genes of XLOC_159404, XLOC_031922, XLOC_161072, XLOC_065271, and Bank1 are shown in Table 4, and the pathways are listed in Supplementary material 13.

**Table 4:** The target gene of lncRNA XLOC_159404, XLOC_031922, XLOC_161072, XLOC_065271 and Bank1.

lncRNA Gene ID	Co-expression		Co-location	
	mRNA Gene ID	mRNA Symbol	mRNA Gene ID	mRNA Symbol
XLOC_159404	ENSMUSG00000024048	Myl12a	ENSMUSG00000031132	Cd40lg
	ENSMUSG00000067562	Dmrtc1c1	ENSMUSG00000031133	Arhgef6
	ENSMUSG00000034164	Emid1	ENSMUSG00000031130	Brs3
	ENSMUSG00000042289	Hsd3b7	ENSMUSG00000031131	Vgll1
	ENSMUSG00000023047	Amhr2	ENSMUSG00000053852	Adgrg4
	ENSMUSG00000026463	Atp2b4	ENSMUSG00000067873	Htatsf1
	ENSMUSG00000116024	Gm49527		
	ENSMUSG00000026923	Notch1		
	ENSMUSG00000043298	Smco3		
	ENSMUSG00000081607	Gm15294		
	ENSMUSG00000042485	Mustn1		
	ENSMUSG00000110040	Gm49369		
	ENSMUSG00000044702	Palb2		
XLOC_031922	ENSMUSG00000024841	Eif1ad	ENSMUSG00000060807	Serpina6
	ENSMUSG00000027797	Dclk1	ENSMUSG00000079015	Serpina1c
	ENSMUSG00000031015	Swap70	ENSMUSG00000066366	Serpina1a
	ENSMUSG00000090451	Gm6133	ENSMUSG00000071178	Serpina1b
	ENSMUSG00000039220	Ppp1r10	ENSMUSG00000071179	Serpina16
	ENSMUSG00000047793	Sned1	ENSMUSG00000071177	Serpina1d
	ENSMUSG00000053178	Mterf1b	ENSMUSG00000021081	Serpina1f
	ENSMUSG00000024620	Pdgfrb		
	ENSMUSG00000032806	Slc10a3		
	ENSMUSG00000054342	Kcnn4		
	ENSMUSG00000056204	Pgpep1		
	ENSMUSG00000031146	Plp2		
	ENSMUSG00000023022	Lima1		
	ENSMUSG00000030111	A2m		
	ENSMUSG00000028268	Gbp3		
	ENSMUSG00000050914	Ankrd37		
	ENSMUSG00000011958	Bnip2		
	ENSMUSG00000038925	E330034G19Rik		
	ENSMUSG00000037242	Clic4		
	ENSMUSG00000028463	Car9		
	ENSMUSG00000028789	Azin2		
XLOC_161072	ENSMUSG00000037617	Spag1	ENSMUSG00000067441	H2afb1
XLOC_065271	ENSMUSG00000018678	Sp2	ENSMUSG00000035842	Ddx11
	ENSMUSG00000034157	Cipc	ENSMUSG00000052105	Mtcl1
	ENSMUSG00000042515	Pwwp3b		
	ENSMUSG00000022744	Cldnd1		
	ENSMUSG00000110104	Gm45717		
	ENSMUSG00000069184	Zfp72		
	ENSMUSG00000049232	Tigd2		
	ENSMUSG00000064289	Tank		
	ENSMUSG00000050786	Ccdc126		
Bank1	None	None	ENSMUSG00000037922	Bank1

## Discussion

This study revealed that changes in the intracellular proteome, mRNA transcriptome, and lncRNA expression levels occurred in neurons under NMDAR knockdown and H/R exposure. The experimental results showed that the differential expression trend in the si-NMDAR+H/R:si-NC comparison was essentially the same as that in the other two comparisons in terms of protein, mRNA and lncRNA expression levels. For example, Rpl6 was the most upregulated protein in the si-NMDAR+H/R:si-NC comparison it was also upregulated and ranked second in the si-NMDAR+H/R:si-NMDAR comparison. Tfrc was the second most upregulated protein in the si-NMDAR+H/R:si-NC comparison and the first-ranking upregulated protein the si-NMDAR:si-NC comparison (Table 1; Supplementary material 14). We consider that for proteins/mRNAs/lncRNAs with the same regulatory trends between the si-NMDAR:si-NC comparison and the si-NMDAR+H/R:si-NC comparison, the changes were mainly affected by NMDAR knockdown but not related to H/R injury; similarly, for proteins/mRNAs/lncRNAs with the same regulatory trends between the si-NMDAR+H/R:si-NC comparison and the si-NMDAR+H/R:si-NMDAR comparisons, the changes were mainly affected by H/R injury but not related to NMDAR knockdown. We also noticed that no proteins/mRNAs/lncRNAs had the same regulatory trends between the si-NMDAR:si-NC comparison and the si-NMDAR+H/R:si-NMDAR comparison. Therefore, we focused on the proteins/mRNAs/lncRNAs whose regulatory trends were completely opposite between the si-NMDAR:si-NC comparison and the si-NMDAR+H/R:si-NMDAR comparison. The expression levels of these molecules were significantly changed under the influence of NMDAR knockdown, but the expression changes were significantly reversed after H/R exposure.

In the proteome analysis, the expression levels of Rps9, Rpl18, and Rpl15 were significantly reduced after NMDAR knockdown in neuronal cells but significantly increased after NMDAR knockdown followed by H/R. These three proteins are all ribosomal component proteins. We were unable to find any relevant studies on the effects of NMDAR or H/R injury on the protein expression of Rps9, Rpl18, and Rpl15. We speculate that knockdown of NMDAR in neurons may have inhibited the PI3K/mTOR signaling pathway and limited ribosome synthesis (Xu et al., 2018; Fabbri et al., 2021), which was reflected in the decreased expression levels of the Rps9, Rpl18, and Rpl15 proteins; when H/R was applied, various compensatory pathways for ribosome synthesis were activated, causing a significant rebound in the expression of these proteins (Branco-Price et al., 2008).

Furthermore, in our analysis of the mRNA transcriptome, we noticed that the changes in mRNA expression levels did not correspond to the changes in protein expression levels. For example, in the si-NMDAR:si-NC comparison, the protein expression levels of Rps9, Rpl18, and Rpl15 were significantly reduced, but the mRNA expression levels were not significantly altered; in contrast, in the si-NMDAR:si-NC comparison, the mRNA expression levels of Bank1 and Pcp4l1 were significantly increased, but the protein expression levels were not significantly altered (Tables 1, 2; Supplementary material 14). This difference may have been caused by the time-series nature of intracellular molecular functional responses. Since we collected cells for proteome and transcriptome detection at a single time point after NMDAR knockdown and H/R for 12 h, there may have been time for an increase in mRNA expression but insufficient time for an increase in protein translation. To explain this phenomenon, continuous observations at multiple time points are needed in future studies.

After knockdown of NMDAR in neuronal cells, the mRNA expression levels of Bank1 and Pcp4l1 were significantly increased; however, when H/R was applied, the expression levels of these two mRNAs significantly decreased. GO enrichment analysis showed that these mRNAs are involved in “binding” and “protein binding” processes. Bank1, whose full name is B cell scaffold protein with ankyrin repeats 1, is a negative regulator of B cell activation. (Gómez Hernández et al., 2021) It has been reported that the intron polymorphism of the Bank1 gene is associated with the risk of anti-NMDAR encephalitis. (Shu et al., 2021) Pcp4l1, whose full name is Purkinje cell protein 4-like 1 is a potential calmodulin inhibitor. (Morgan and Morgan, 2012) We were unable to find any relevant studies on the effect of NMDAR or H/R injury on Pcp4l1 mRNA expression. We speculate that the expression level of Pcp4l1 was significantly increased after knockdown of NMDAR but decreased after H/R, which may have been related to the disturbance of intracellular calcium ions in neurons.

In the analysis of lncRNA expression levels, we noticed that the regulation of lncRNA expression had many similarities with the regulation of mRNA expression. For example, in the si-NMDAR:si-NC comparison, the mRNA expression levels of Gpc5 and Lrrc69 were significantly reduced, and the associated lncRNA expression levels were also significantly reduced; in the si-NMDAR+H/R:si-NMDAR comparison, the expression level of Bank1 was significantly reduced, and the associated lncRNA expression level was also significantly reduced (Tables 2, 3; Supplementary material 14). We consider that the expression of both lncRNAs and mRNAs is regulated at the transcriptional level, so there is a certain correlation. Notably, the “HIF-1 signaling pathway” was associated with the coexpression in all three comparisons; thus, we speculate that lncRNAs are expressed earlier than mRNAs and proteins in response to hypoxia, which is also consistent with our previous speculation on the time-series nature of intracellular molecular functional responses. In addition, we noticed several newly discovered lncRNAs: XLOC_159404 and XLOC_031922 were significantly upregulated after NMDAR knockdown but significantly downregulated after H/R, while XLOC_161072 and XLOC_065271 were significantly downregulated after NMDAR knockdown but significantly upregulated after H/R. Although the predicted target genes are involved in multiple GO functions and multiple pathways, we were unable to find any relevant studies on the effects of NMDAR or H/R injury on their expression, so their specific roles need to be further explored.

Furthermore, we observed that the expression levels of the Gapdh protein, which is often used as an internal reference protein in quantification experiments such as western blotting, were significantly downregulated in both the si-NMDAR:si-NC comparison and the si-NMDAR+H/R:si-NC comparison (Table 1; Supplementary material 14). Therefore, Gapdh is not suitable as an internal reference gene in experimental studies involving reduced NMDAR expression levels.

The limitation of this study was that it is difficult to conclude a clear pathway or network of the identified changes, because these molecules of differential expression involve a variety of different biological functions and processes. Therefore, we focused on the molecules whose expression levels were completely opposite after NMDAR knockdown and H/R, try to reveal the effects of NMDAR knockdown and H/R injury on neurons, and look for the potential target that may be very important in the treatment of cerebral ischemia. In this article, we present these changes truthfully without bias, providing research basis for researchers in related fields.

## Conclusion

This study investigated the regulation profiles of the intracellular proteome, mRNA transcriptome and lncRNA expression levels in neurons after NMDAR knockdown and H/R injury. We focused on the proteins/mRNAs/lncRNAs whose expression levels were completely opposite after NMDAR knockdown and H/R. The proteins Rps9, Rpl18, and Rpl15 and the lncRNAs XLOC_161072 and XLOC_065271 were significantly downregulated after NMDAR knockdown but upregulated after H/R; in contrast, the mRNAs Bank1 and Pcp4l1 and the lncRNAs XLOC_159404 and XLOC_031922 were significantly upregulated after NMDAR knockdown but downregulated after H/R. These molecules are involved in multiple biological functions and signaling pathways, and their roles in neurons that lack NMDAR and undergo H/R injury deserve further study. Additionally, we found that lncRNAs respond fastest to hypoxic stimulation and that Gapdh is not suitable as a reference protein for experiments involving proteins in which NMDAR expression is reduced.

## Data availability statement

All data generated or analyzed during this study are included in this published article (and its supplementary information files). The mass spectrometry proteomics data have been deposited at the ProteomeXchange Consortium (http://proteomecentral.proteomexchange.org) via the iProX partner repository with the dataset identifier PXD036251. The RNA-Seq data has been deposited at the GEO database (https://www.ncbi.nlm.nih.gov/geo/) with the dataset identifier GSE214716.

## Author contributions

JH: conceptualization, methodology, resources, data curation, writing – review and editing, supervision, and funding acquisition. KC and YS: validation and investigation. QY: investigation, formal analysis, data curation, writing – original draft, visualization, supervision, project administration, and funding acquisition. All authors contributed to the article and approved the submitted version.

## Funding

This work was supported by the National Natural Science Foundation of China (grant number 81902292), Jilin Province Department of Science and Technology (grant number 20210101312JC), Jilin Provincial Department of Finance (grant number 2020SCZ10), Jilin province health youth science and technology key project (grant number 2019Q018), Jilin Provincial Department of Finance (grant number 2019SCZ010), and Norman Bethune Health Science Center of Jilin University (grant number 2020B60).

## Conflict of interest

The authors declare that the research was conducted in the absence of any commercial or financial relationships that could be construed as a potential conflict of interest.

## Publisher’s note

All claims expressed in this article are solely those of the authors and do not necessarily represent those of their affiliated organizations, or those of the publisher, the editors and the reviewers. Any product that may be evaluated in this article, or claim that may be made by its manufacturer, is not guaranteed or endorsed by the publisher.

## Footnotes

^1^ http://biodev.extra.cea.fr/DSIR/DSIR.html

^2^ http://tophat.cbcb.umd.edu

^3^ http://ccb.jhu.edu/software/hisat2

^4^ http://code.google.com/p/rna-star

## Supplementary material

SUPPLEMENTARY MATERIAL 1 Cluster analysis heatmaps showing the differentially expressed proteins in each comparison. The horizontal axis indicates the grouping, the vertical axis indicates the differentially expressed proteins, the left side indicates the clustering of proteins according to the degree of expression similarity, the right side indicates the names of proteins, and the top indicates the clustering of the samples according to the similarity degree of the expression profile. The expression level gradually increases as the color changes from blue to red.

SUPPLEMENTARY MATERIAL 2 GO analysis results showing the terms and proportions of up-/downregulated proteins in the biological process, cellular component, and molecular function categories for each comparison. Comparison of the differentially expressed proteins between (A) the si-NMDAR group and the si-NC group, (B) the si-NMDAR+H/R group and the si-NC group, and (C) the si-NMDAR+H/R group and the si-NMDAR group.

SUPPLEMENTARY MATERIAL 3 Bubble chart of the GO analysis results for the differentially expressed proteins in each comparison. The vertical axis indicates the names of the top 20 terms, the horizontal axis indicates the enrichment factor (the proportion of the candidate proteins among the total proteins), the size of the dot represents the number of differentially expressed proteins in this biological process, and the color of the dot corresponds to the *p*-value.

SUPPLEMENTARY MATERIAL 4 Pie charts showing the top 10 pathways and their proportions of proteins whose expression levels were up-/downregulated in each comparison. The bubble charts show the pathway enrichment results for the differentially expressed proteins in each comparison. The vertical axis indicates the names of the top 20 pathways, the horizontal axis indicates the enrichment factor, the size of the dot represents the number of differentially expressed proteins in this pathway, and the color of the dot corresponds to the *p*-value.

SUPPLEMENTARY MATERIAL 5 Schematic diagram of the ribosome pathway and the proteins it contains.

SUPPLEMENTARY MATERIAL 6 Cluster analysis heatmap showing the differentially expressed mRNAs in each comparison. The horizontal axis indicates the samples, the vertical axis indicates the differentially expressed mRNAs, the left side indicates the clustering of mRNAs according to the degree of expression similarity, the right side indicates the names of the mRNAs, and the top indicates the clustering of the samples according to the similarity degree of the expression profile. The expression level gradually increases as the color changes from blue to red.

SUPPLEMENTARY MATERIAL 7 (A-C) GO analysis results showing the terms and proportions of mRNAs in the biological process, cellular component, and molecular function categories for each comparison. (D-F) Pathway analysis results showing the enrichment results for the differentially expressed mRNAs in each comparison. The vertical axis indicates the names of the top 20 pathways, the horizontal axis indicates the enrichment factor, the size of the dot represents the number of differentially expressed mRNAs in this pathway, and the color of the dot corresponds to the *p*-value.

SUPPLEMENTARY MATERIAL 8 DAG maps of the GO enrichment results for the differentially expressed mRNAs in each comparison. Each node represents a GO term, the boxes represent the top 10 GO terms in terms of enrichment degree, and the color represents the enrichment degree (a darker color represents a higher enrichment degree). Each node displays the name and *p*-value of the term.

SUPPLEMENTARY MATERIAL 9 Cluster analysis heatmap showing the differentially expressed lncRNAs in each comparison. The horizontal axis indicates the samples, the vertical axis indicates the differentially expressed lncRNAs, the left side indicates the clustering of lncRNAs according to the degree of expression similarity, the right side indicates the names of lncRNAs, and the top indicates the clustering of the samples according to the similarity degree of the expression profile. The expression level gradually increases as the color changes from blue to red.

SUPPLEMENTARY MATERIAL 10 GO analysis results showing the terms and proportions of lncRNA target genes in the biological process, cellular component, and molecular function category for each comparison. (A-C) GO analysis results of target genes that were coexpressed with differentially expressed lncRNAs. (D-F) GO analysis results of target genes that were colocalized with differentially expressed lncRNAs.

SUPPLEMENTARY MATERIAL 11 DAG maps of GO enrichment of the differentially expressed lncRNA target genes in each comparison. Each node represents a GO term, the boxes represent the top 10 GO terms with regard to enrichment degree, and the color represents the enrichment degree (a darker color represents a higher enrichment degree). Each node displays the name and *p*-value of the term.

SUPPLEMENTARY MATERIAL 12 Pathway analysis showing the enrichment results of the differentially expressed lncRNAs in each comparison. The vertical axis indicates the names of the top 20 pathways, the horizontal axis indicates the enrichment factor, the size of the dot represents the number of differentially expressed lncRNAs in this pathway, and the color of the dot corresponds to the *p*-value.

SUPPLEMENTARY MATERIAL 13 KEGG pathways enriched for the target genes of lncRNA XLOC_159404, XLOC_031922, XLOC_161072, XLOC_065271 and Bank1.

SUPPLEMENTARY MATERIAL 14 All proteins, mRNAs and lncRNAs that were up-/downregulated in each comparison.
