# Supplementary figures and images for: Effects of N-methyl-D-aspartate receptor knockdown and hypoxia/reoxygenation injury on the neuronal proteome and transcriptome
Source: Front Mol Neurosci. 2022 Dec 15;15:1004375. doi: 10.3389/fnmol.2022.1004375 (PMC9799235; doi:10.3389/fnmol.2022.1004375)

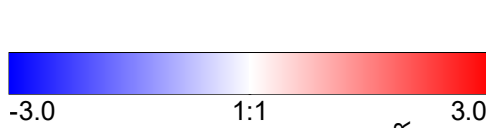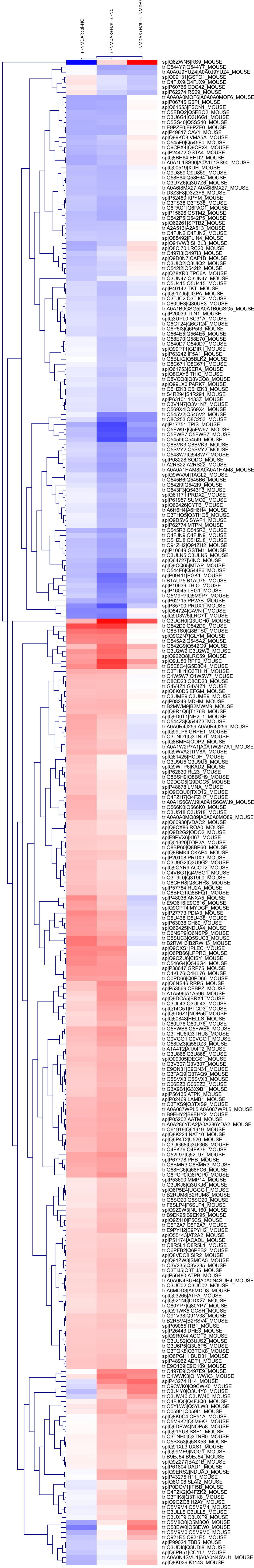

Supplement: SUPPLEMENTARY MATERIAL 1 — Cluster analysis heatmaps showing the differentially expressed proteins in each comparison. The horizontal axis indicates the grouping, the vertical axis indicates the differentially expressed proteins, the left side indicates the clustering of proteins according to the degree of expression similarity, the right side indicates the names of proteins, and the top indicates the clustering of the samples according to the similarity degree of the expression profile. The expression level gradually increases as the color changes from blue to red. [file Data_Sheet_1.PDF]

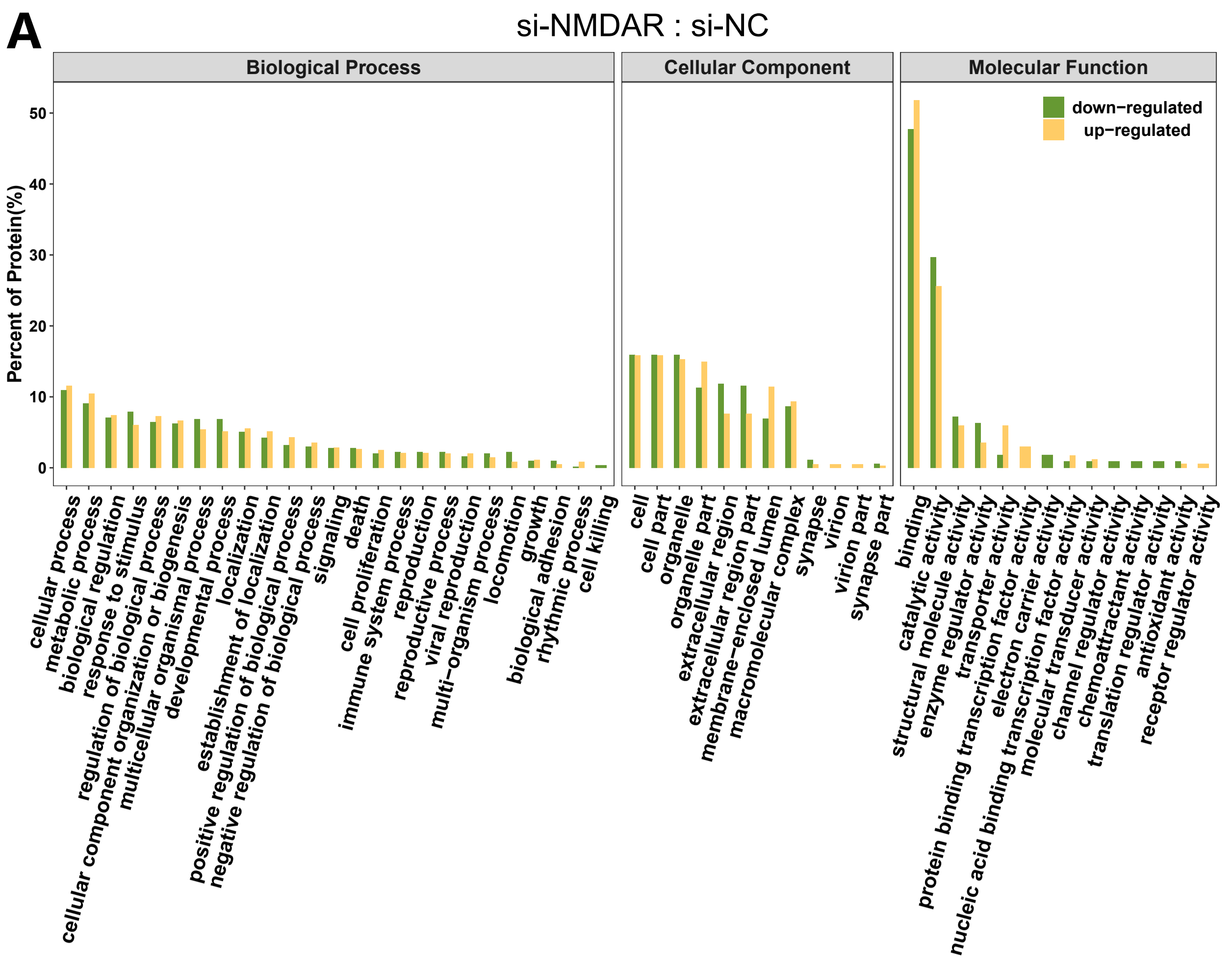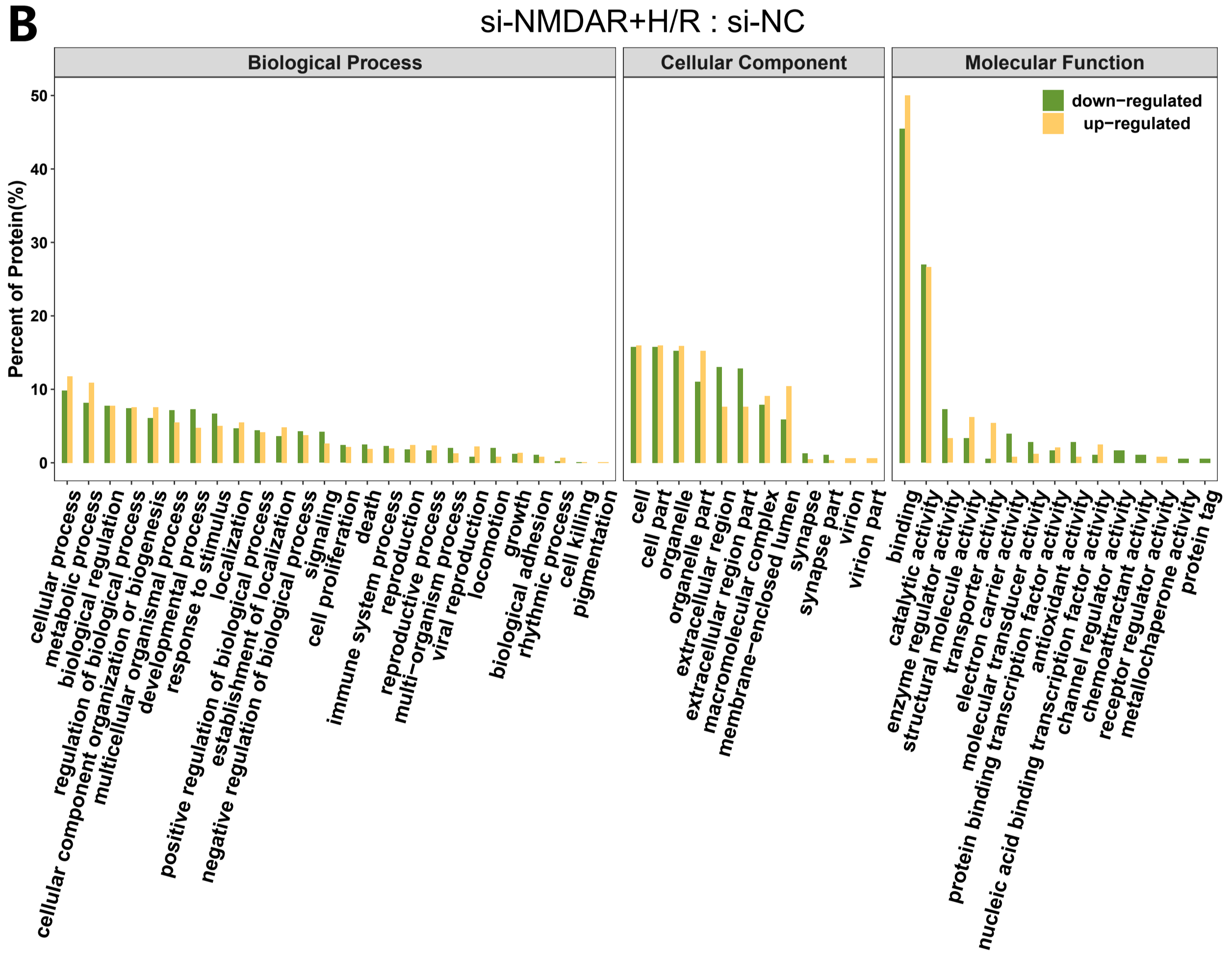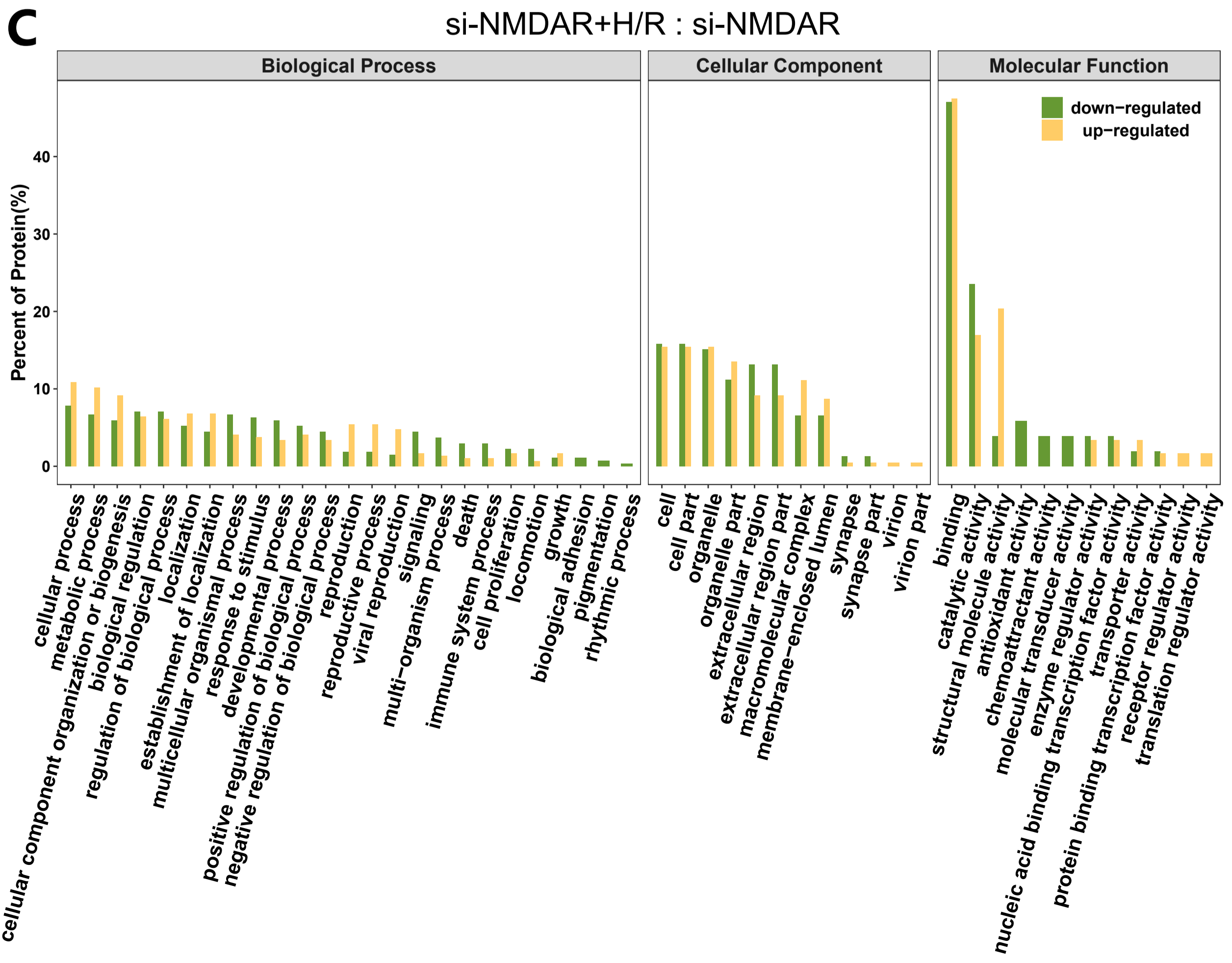

Supplement: SUPPLEMENTARY MATERIAL 2 — GO analysis results showing the terms and proportions of up-/downregulated proteins in the biological process, cellular component, and molecular function categories for each comparison. Comparison of the differentially expressed proteins between (A) the si-NMDAR group and the si-NC group, (B) the si-NMDAR+H/R group and the si-NC group, and (C) the si-NMDAR+H/R group and the si-NMDAR group. [file Data_Sheet_2.PDF]

si-NMDAR : si-NC

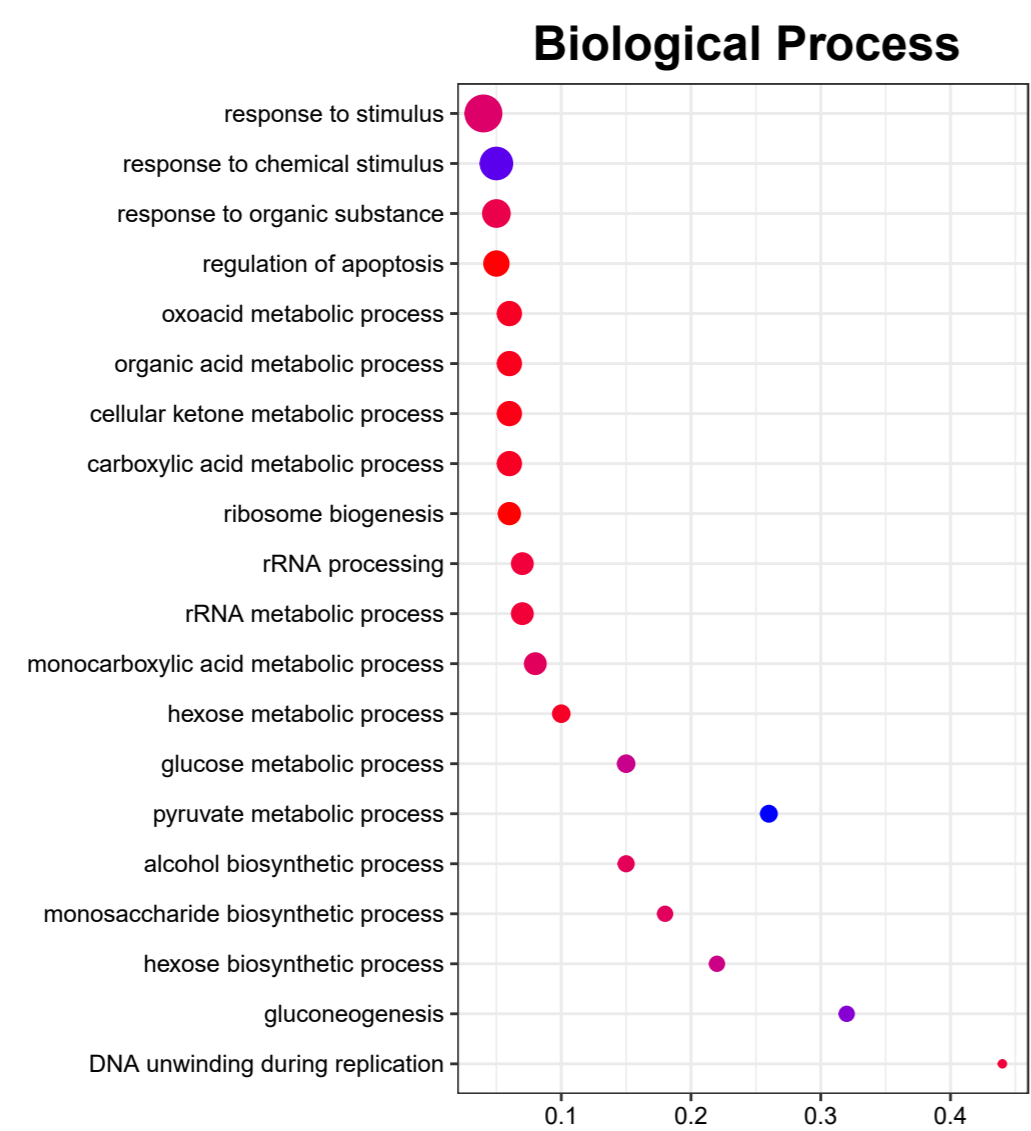

si-NMDAR+H/R : si-NC

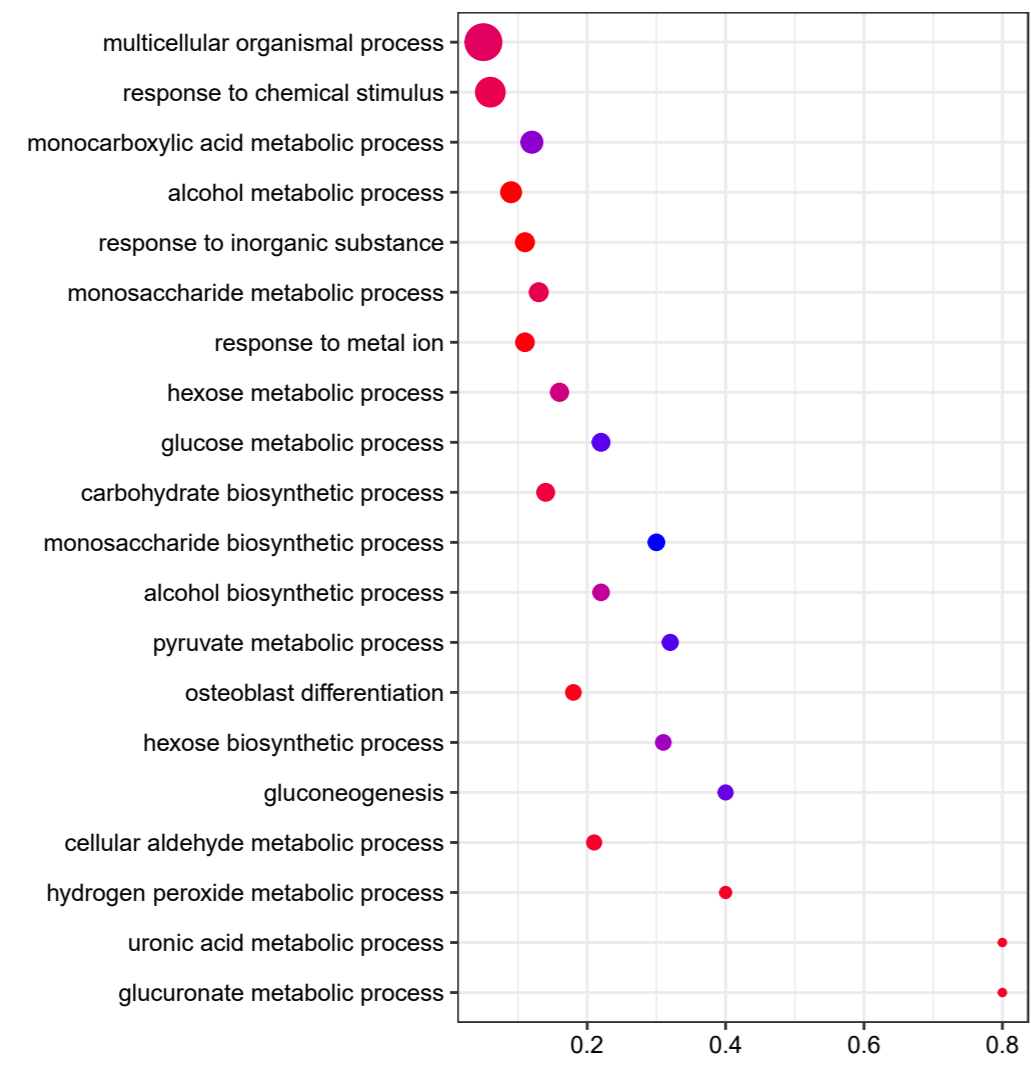

si-NMDAR+H/R : si-NMDAR

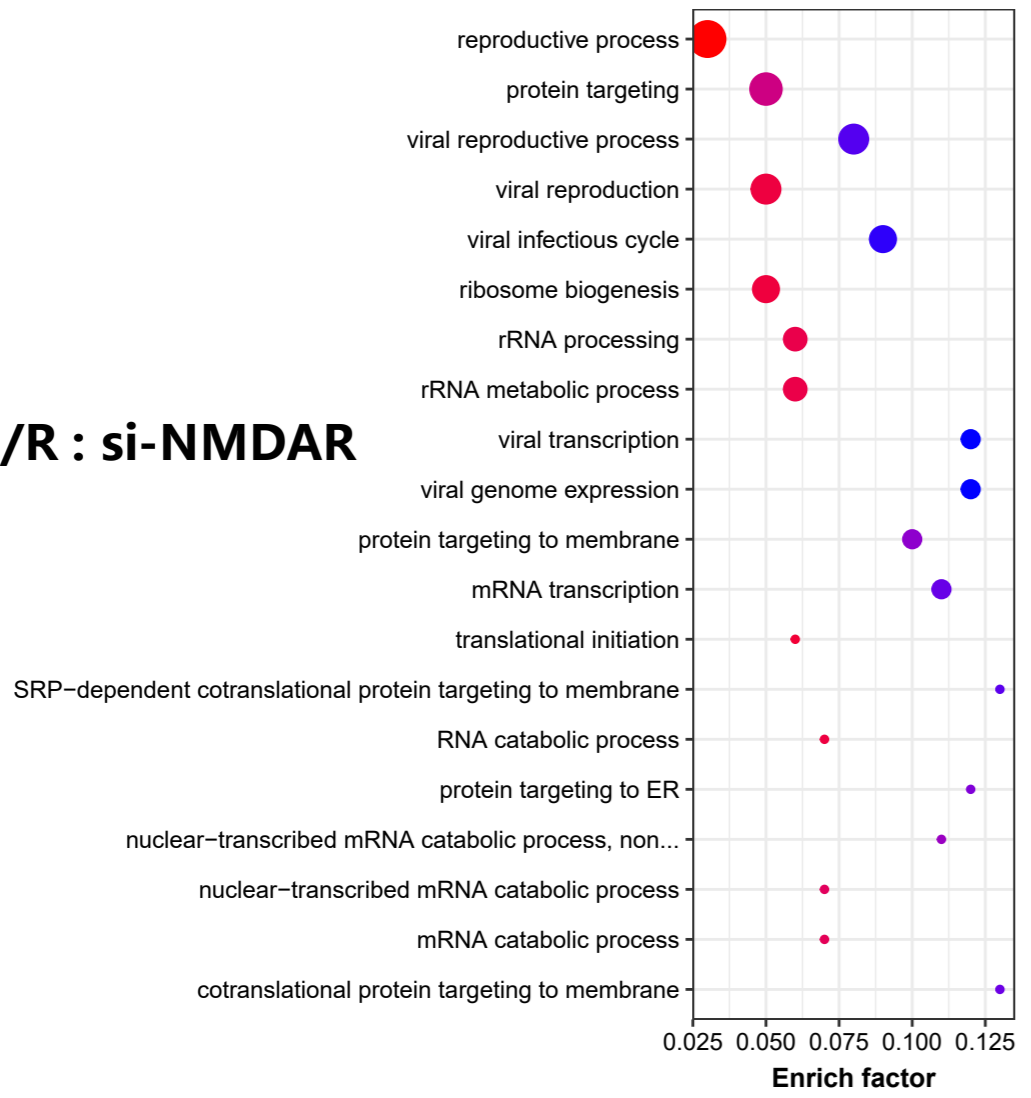

Cellular Component

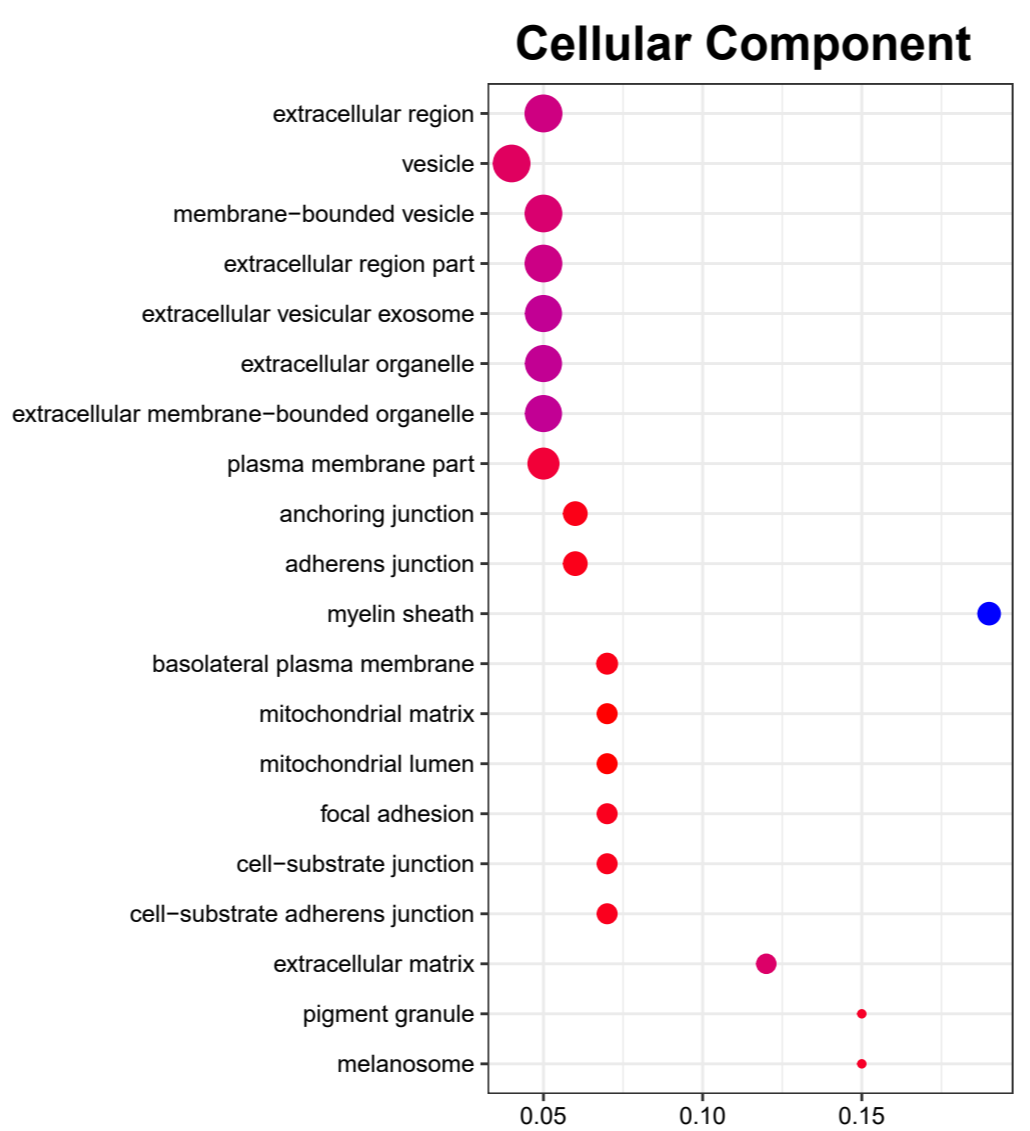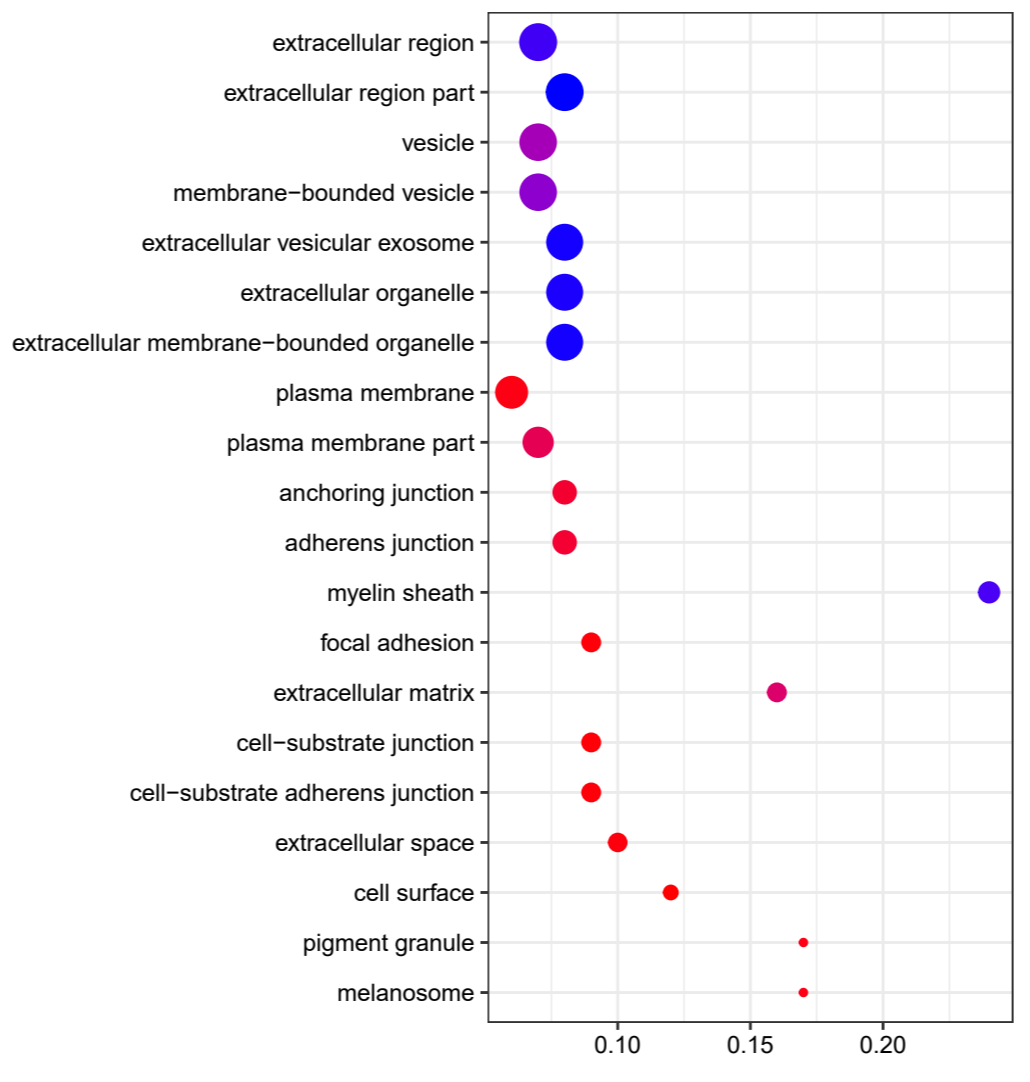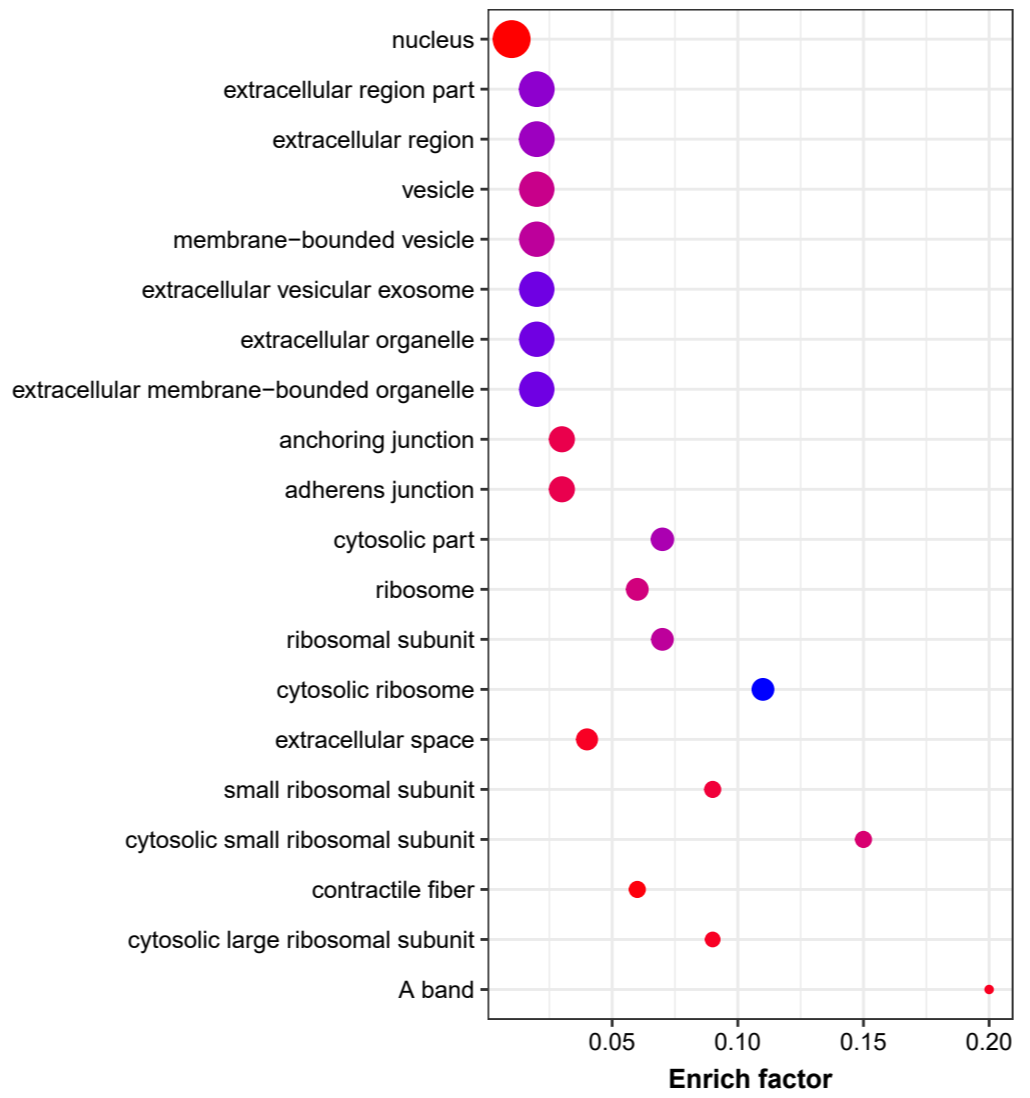

Molecular Function

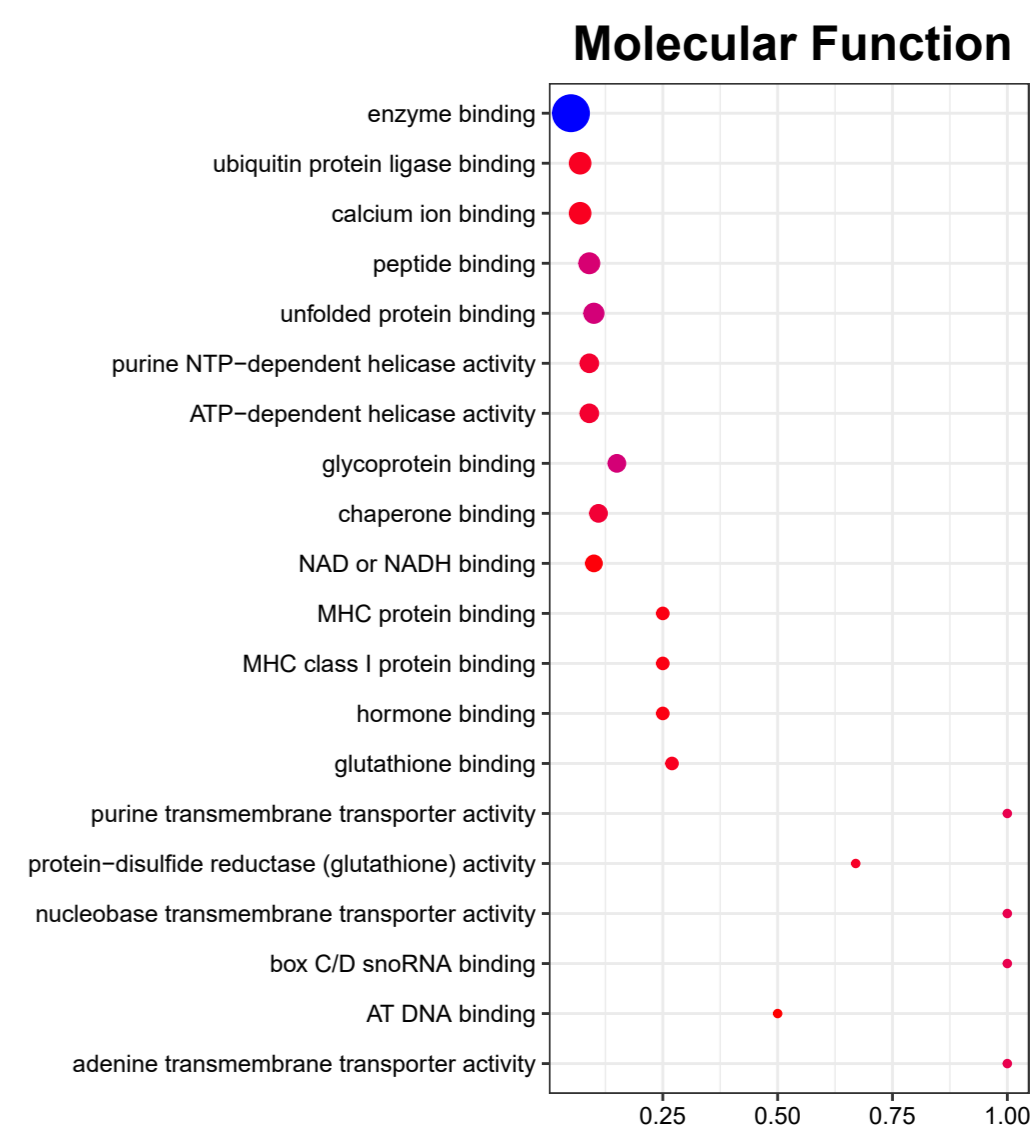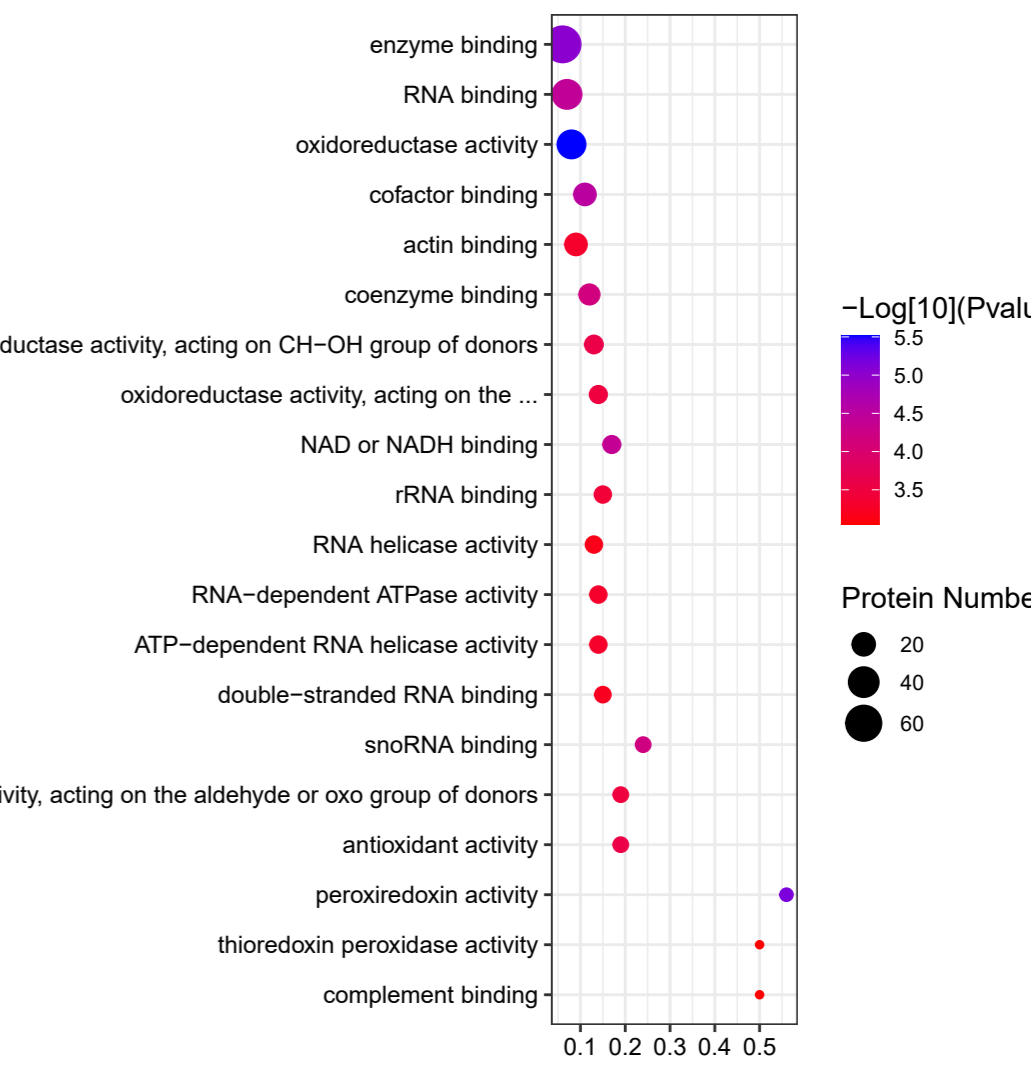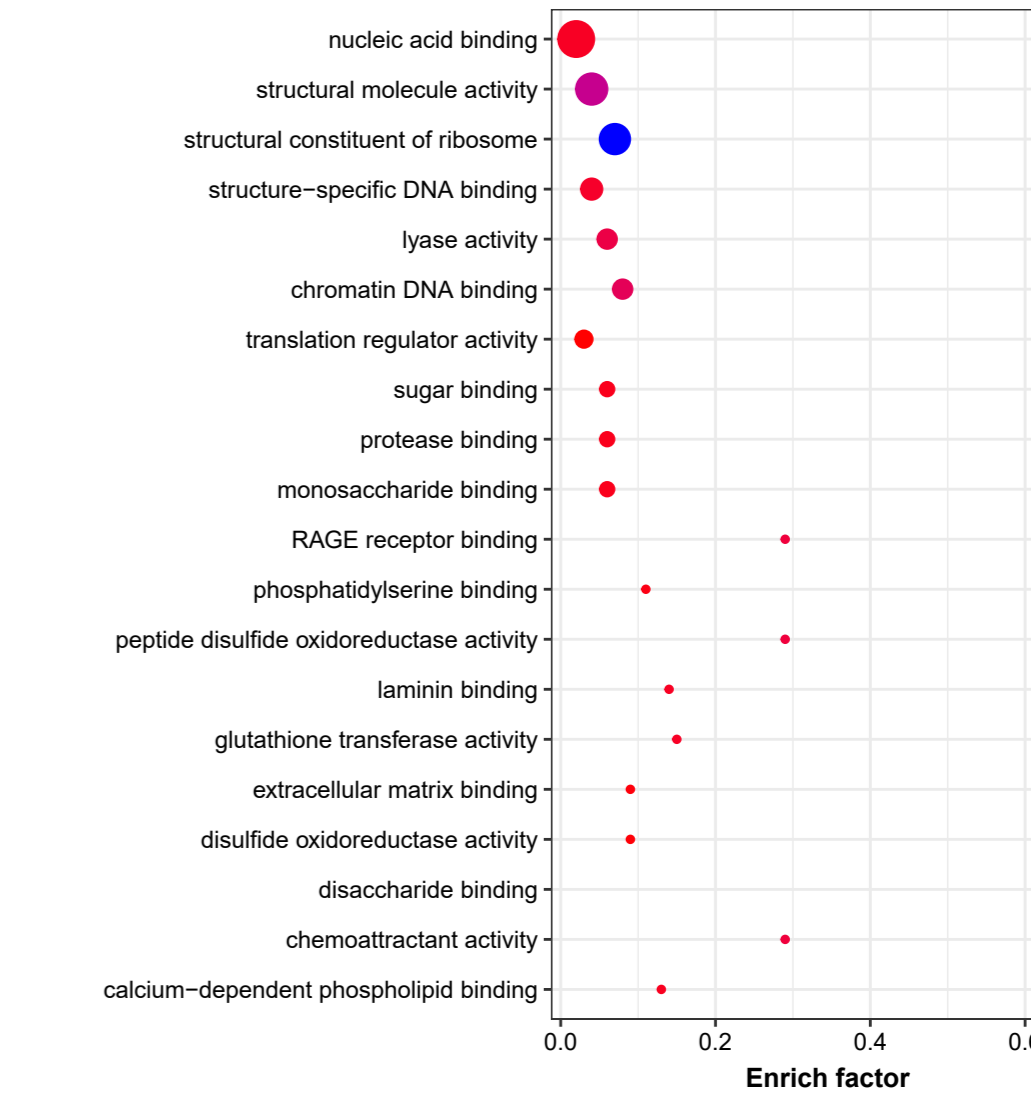

Supplement: SUPPLEMENTARY MATERIAL 3 — Bubble chart of the GO analysis results for the differentially expressed proteins in each comparison. The vertical axis indicates the names of the top 20 terms, the horizontal axis indicates the enrichment factor (the proportion of the candidate proteins among the total proteins), the size of the dot represents the number of differentially expressed proteins in this biological process, and the color of the dot corresponds to the p-value. [file Data_Sheet_3.PDF]

si-NMDAR : si-NC

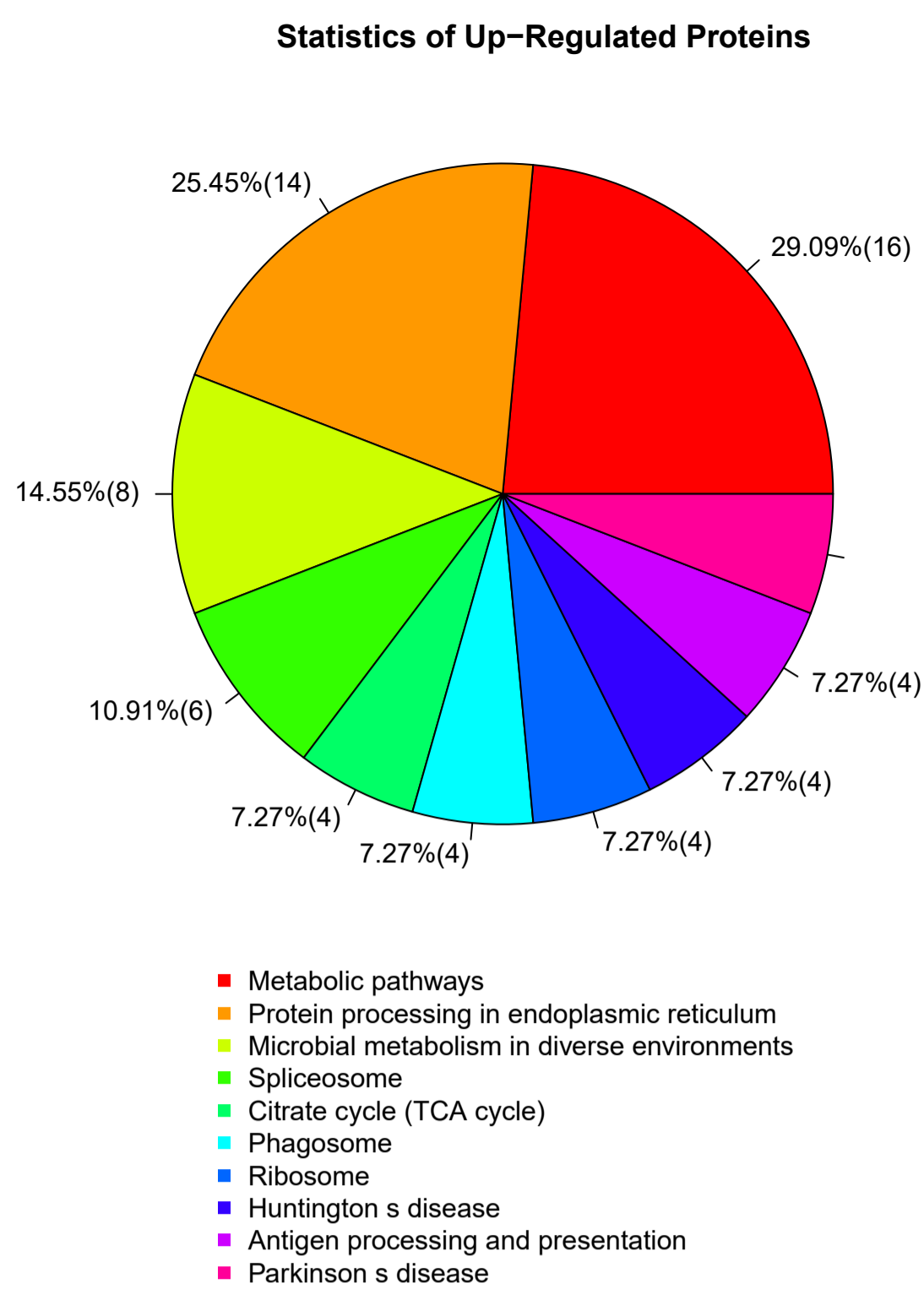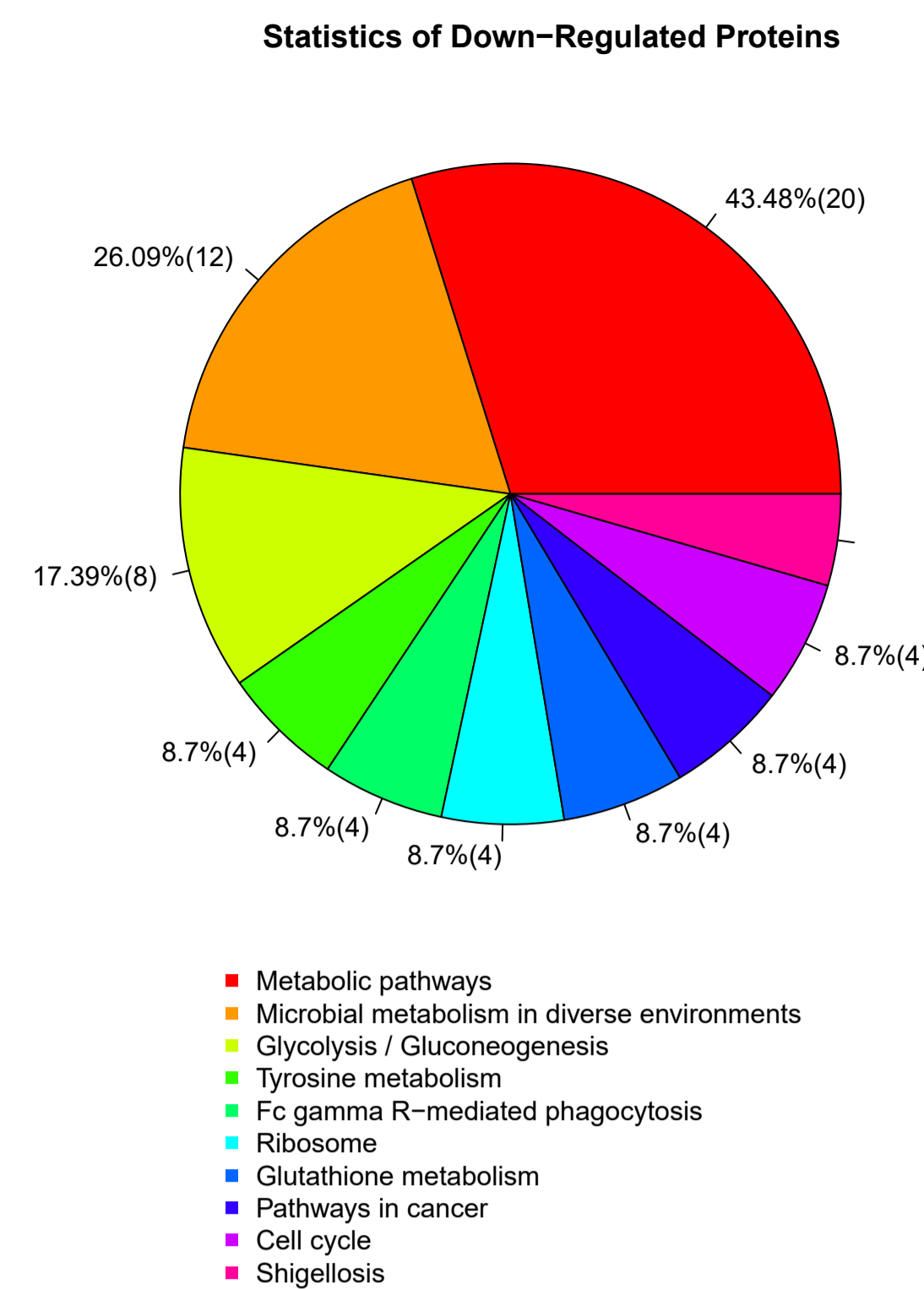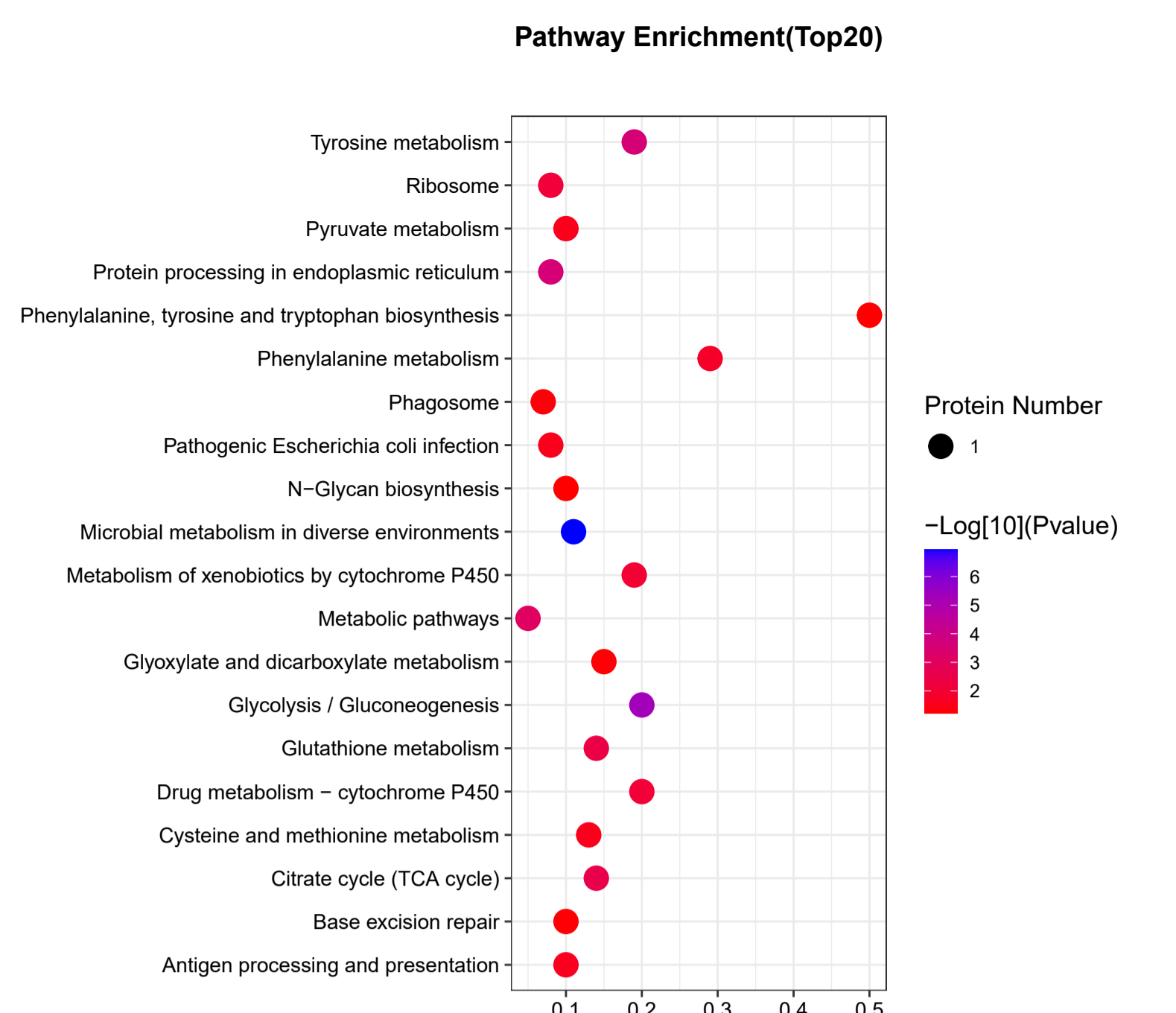

si-NMDAR+H/R : si-NC

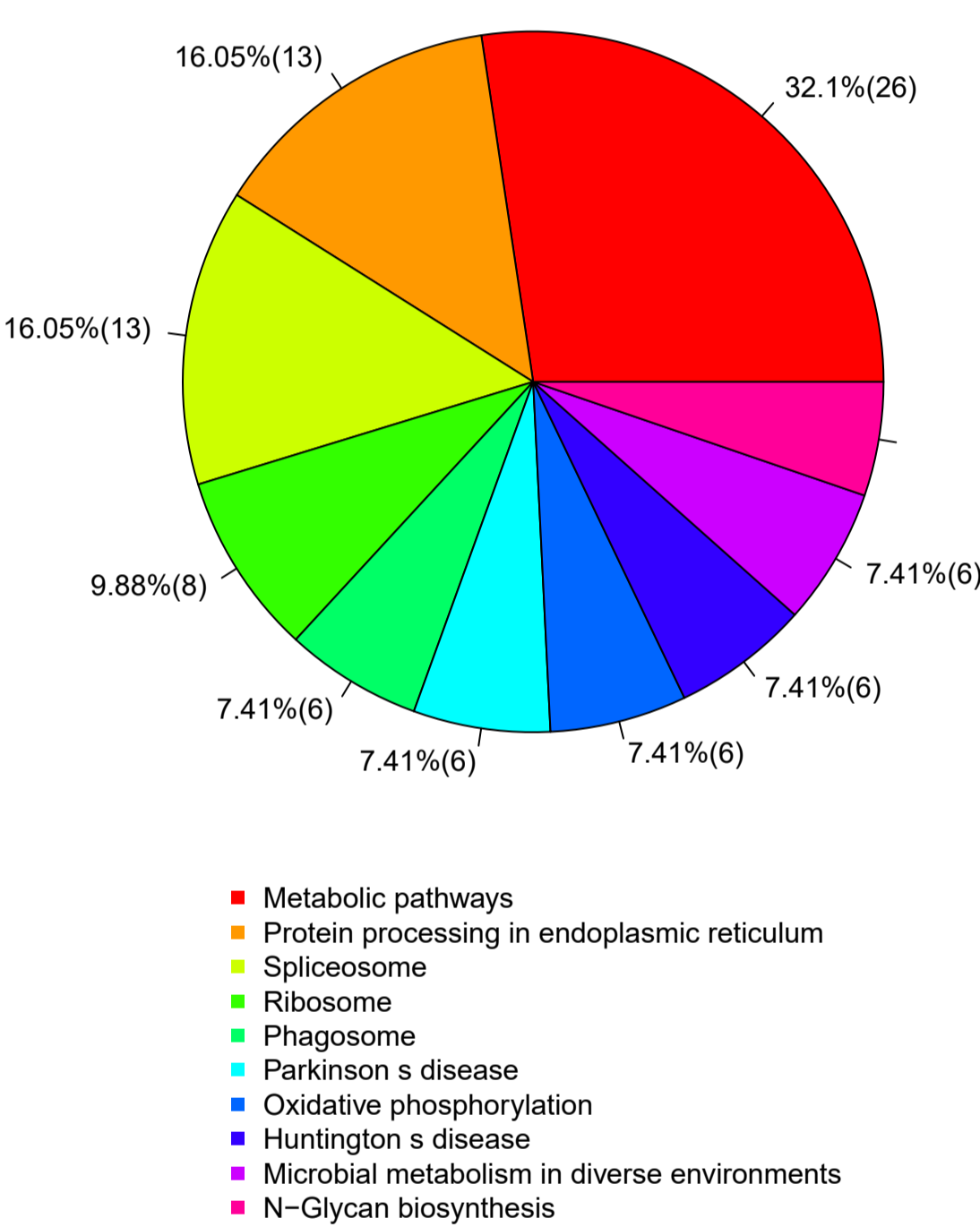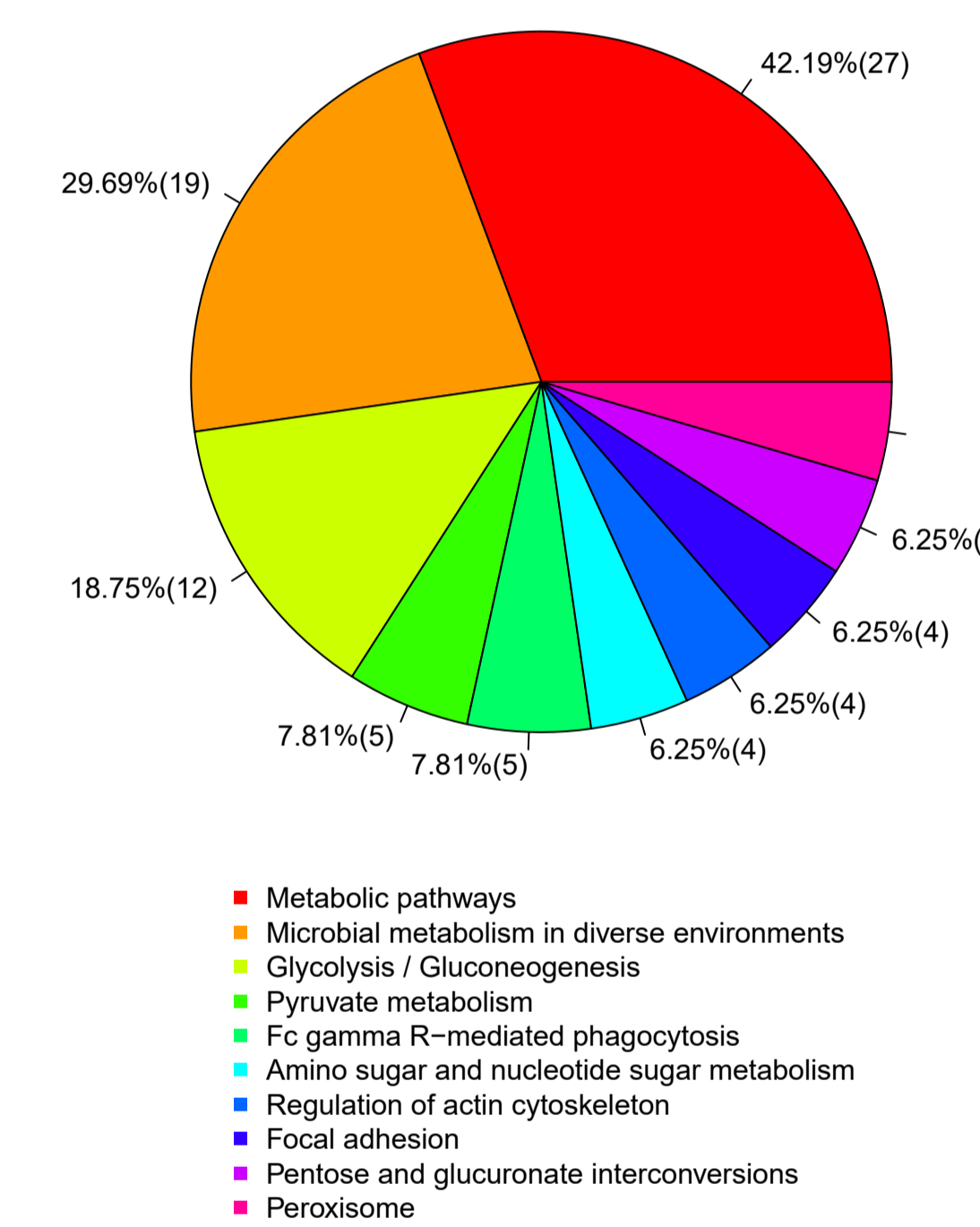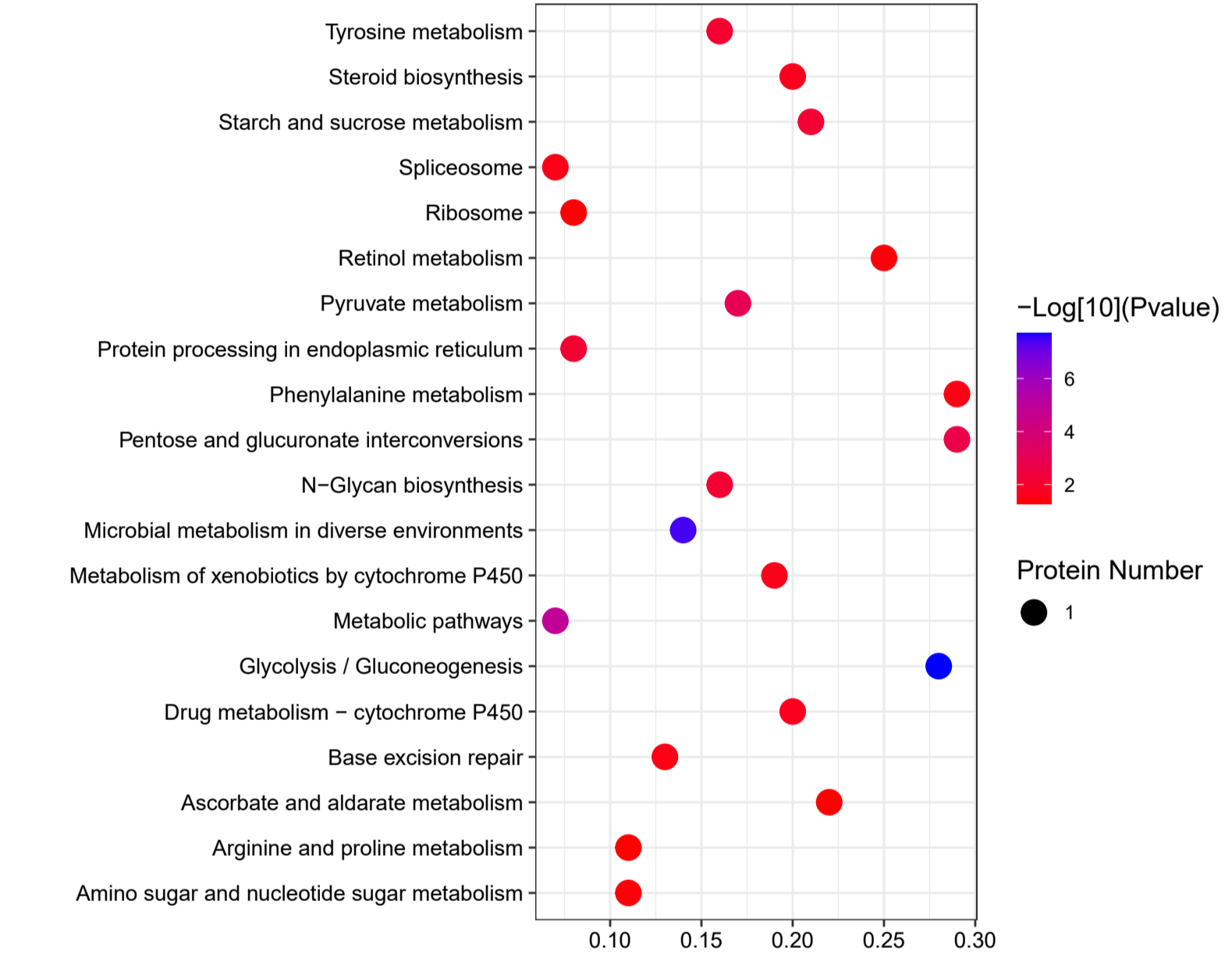

si-NMDAR+H/R : si-NMDAR

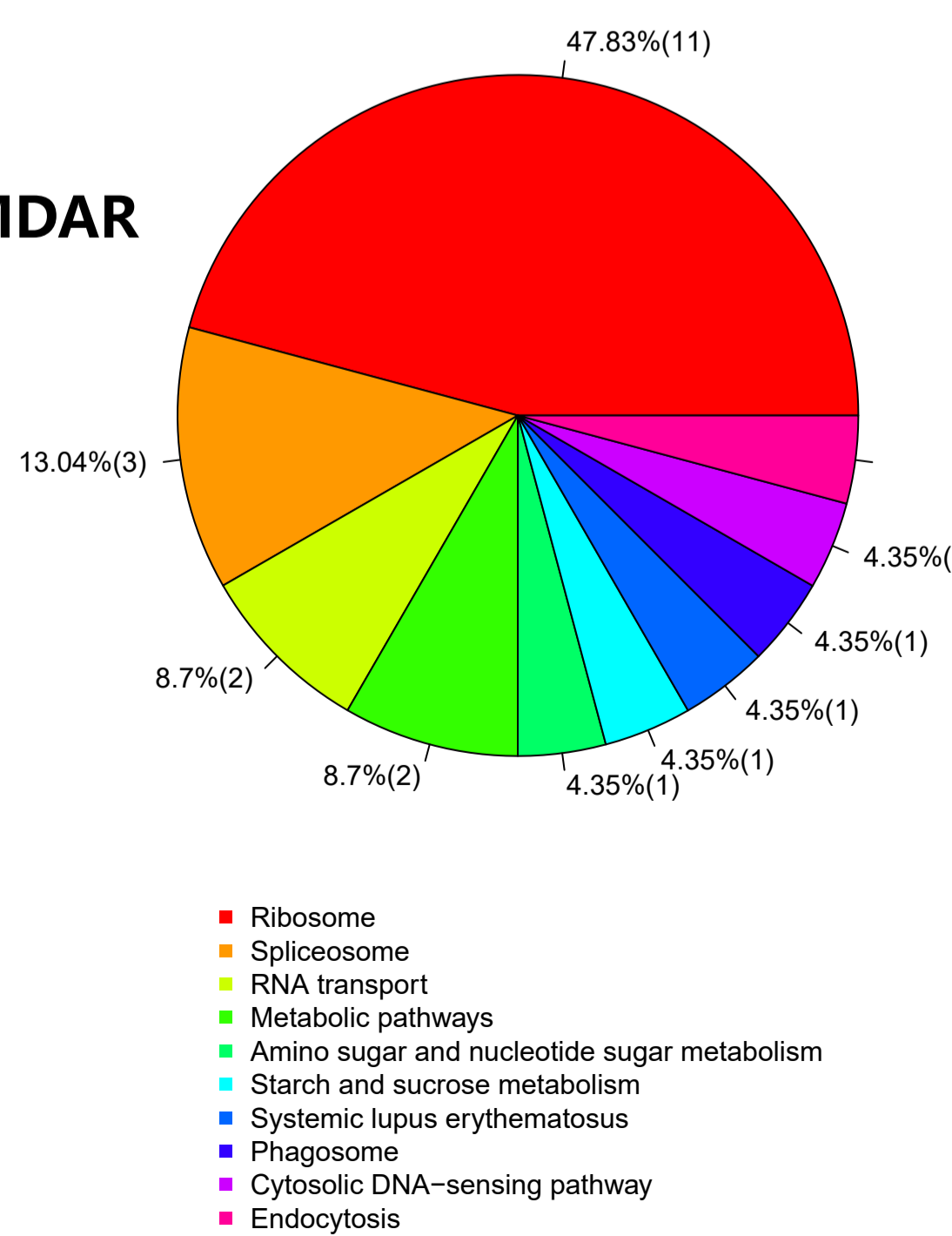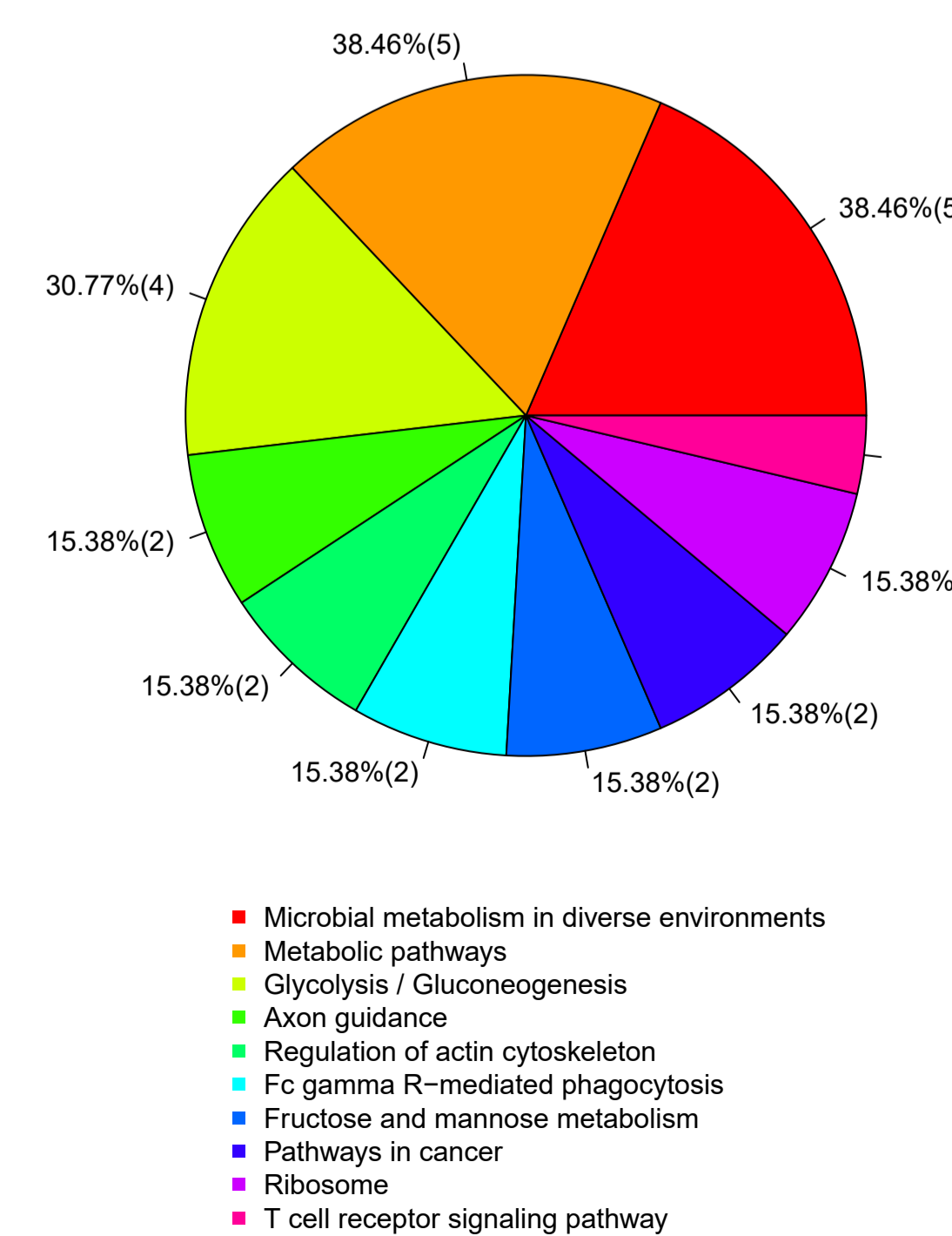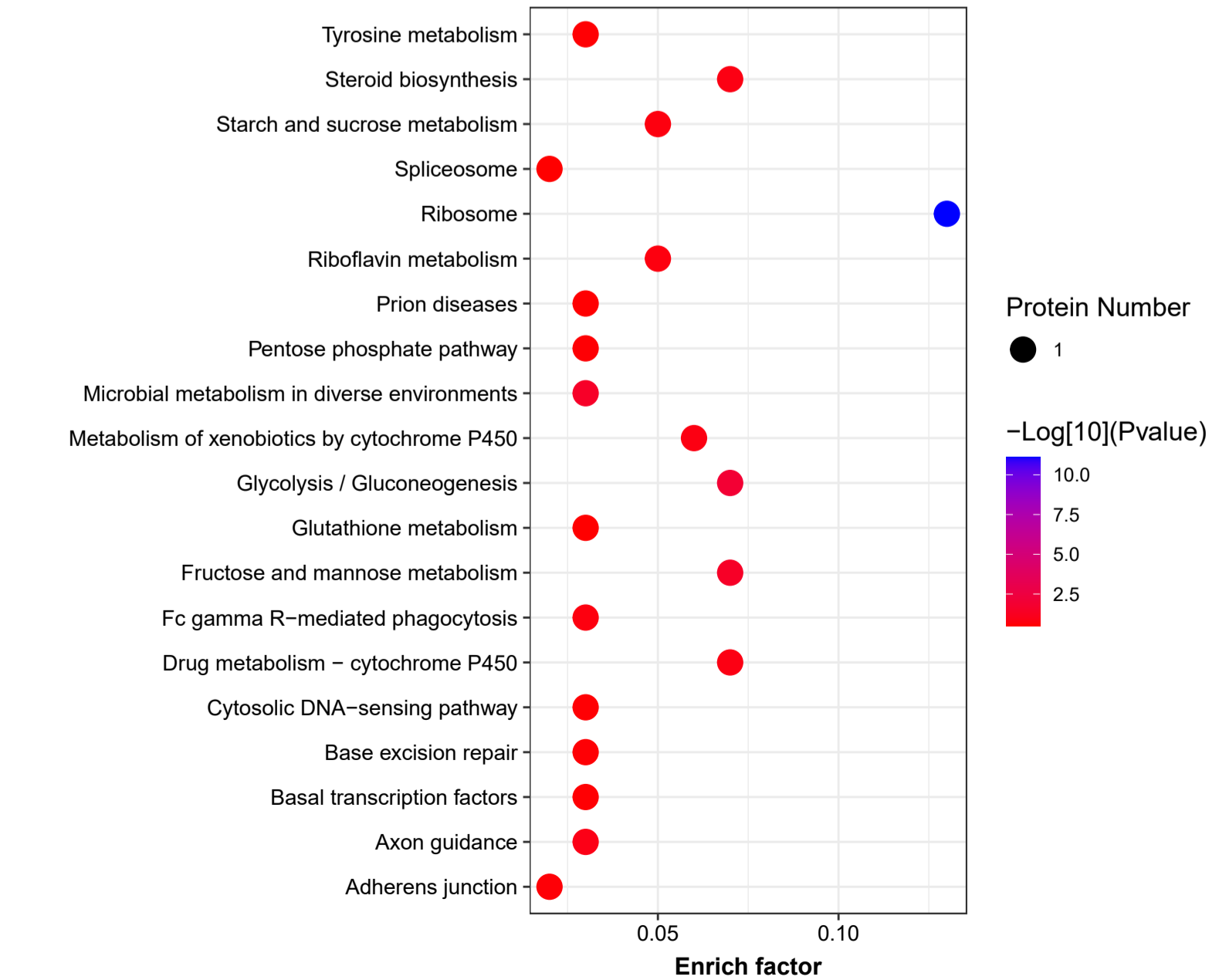

Supplement: SUPPLEMENTARY MATERIAL 4 — Pie charts showing the top 10 pathways and their proportions of proteins whose expression levels were up-/downregulated in each comparison. The bubble charts show the pathway enrichment results for the differentially expressed proteins in each comparison. The vertical axis indicates the names of the top 20 pathways, the horizontal axis indicates the enrichment factor, the size of the dot represents the number of differentially expressed proteins in this pathway, and the color of the dot corresponds to the p-value. [file Data_Sheet_4.PDF]

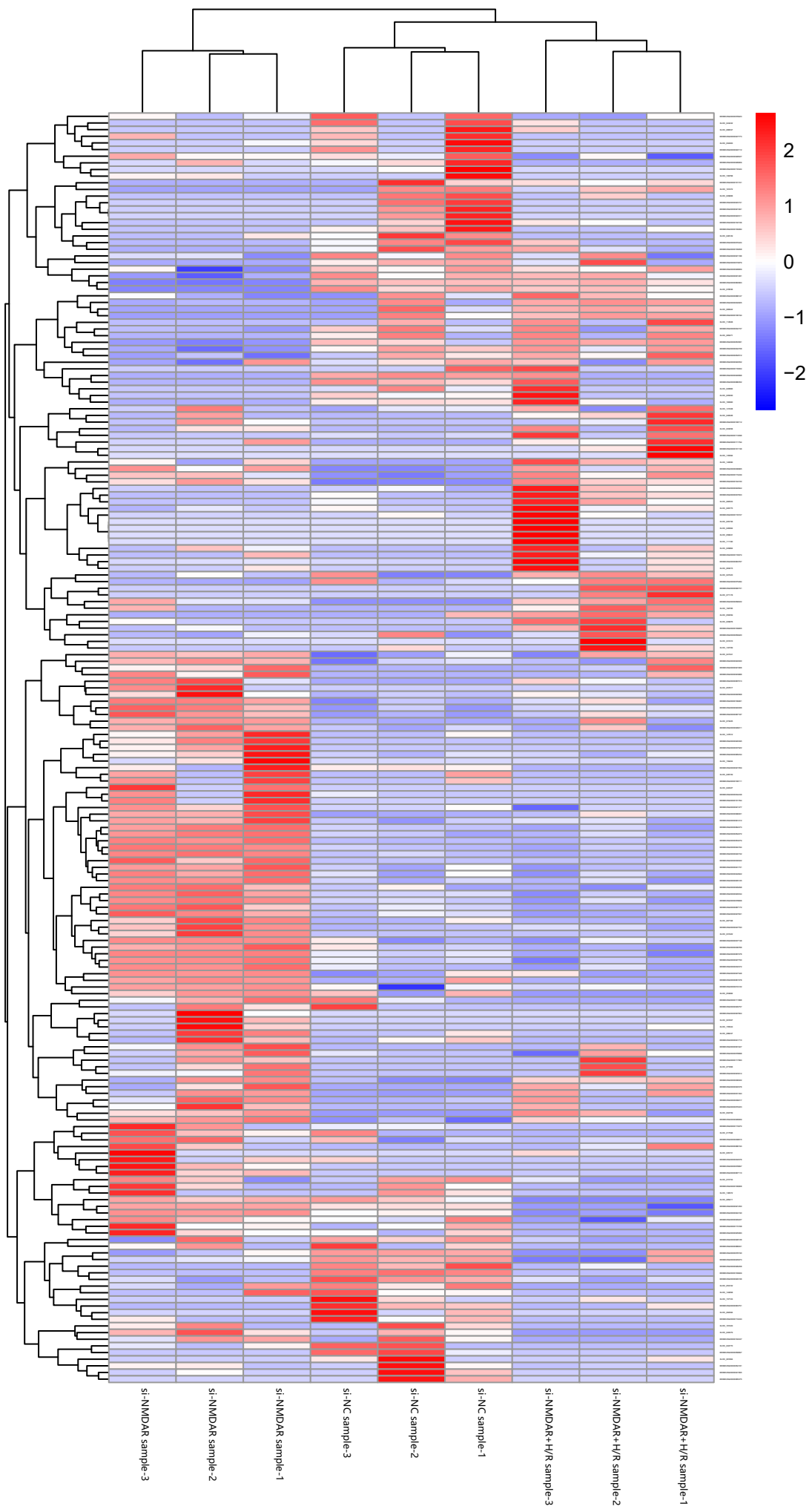

Supplement: SUPPLEMENTARY MATERIAL 6 — Cluster analysis heatmap showing the differentially expressed mRNAs in each comparison. The horizontal axis indicates the samples, the vertical axis indicates the differentially expressed mRNAs, the left side indicates the clustering of mRNAs according to the degree of expression similarity, the right side indicates the names of the mRNAs, and the top indicates the clustering of the samples according to the similarity degree of the expression profile. The expression level gradually increases as the color changes from blue to red. [file Data_Sheet_6.PDF]

A

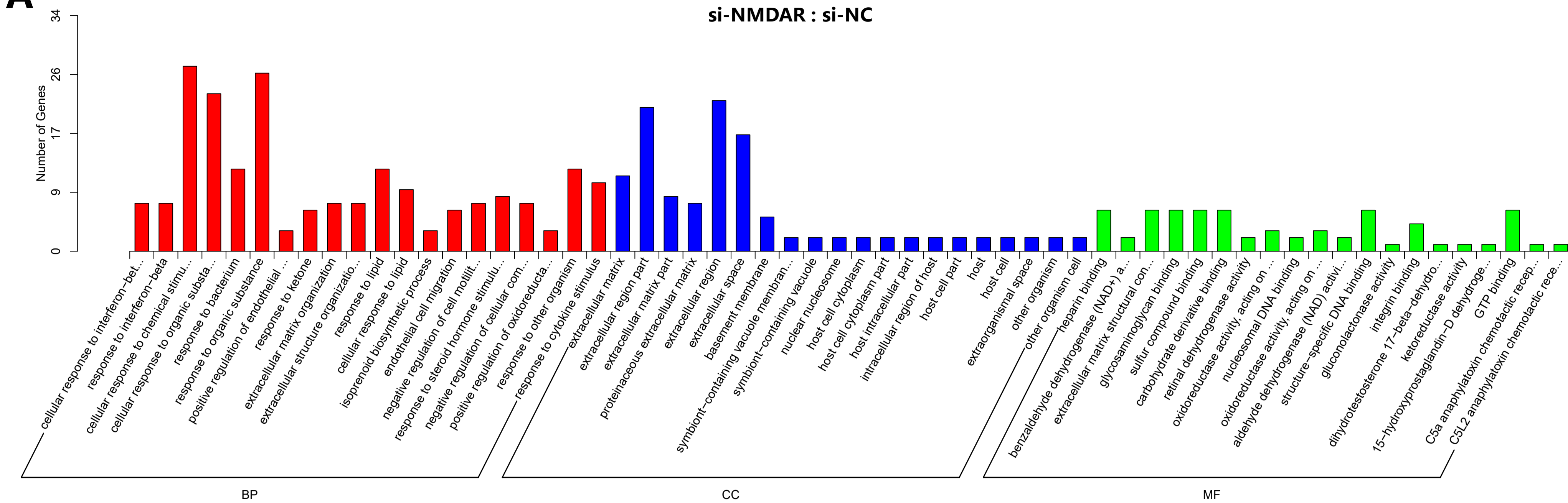

B

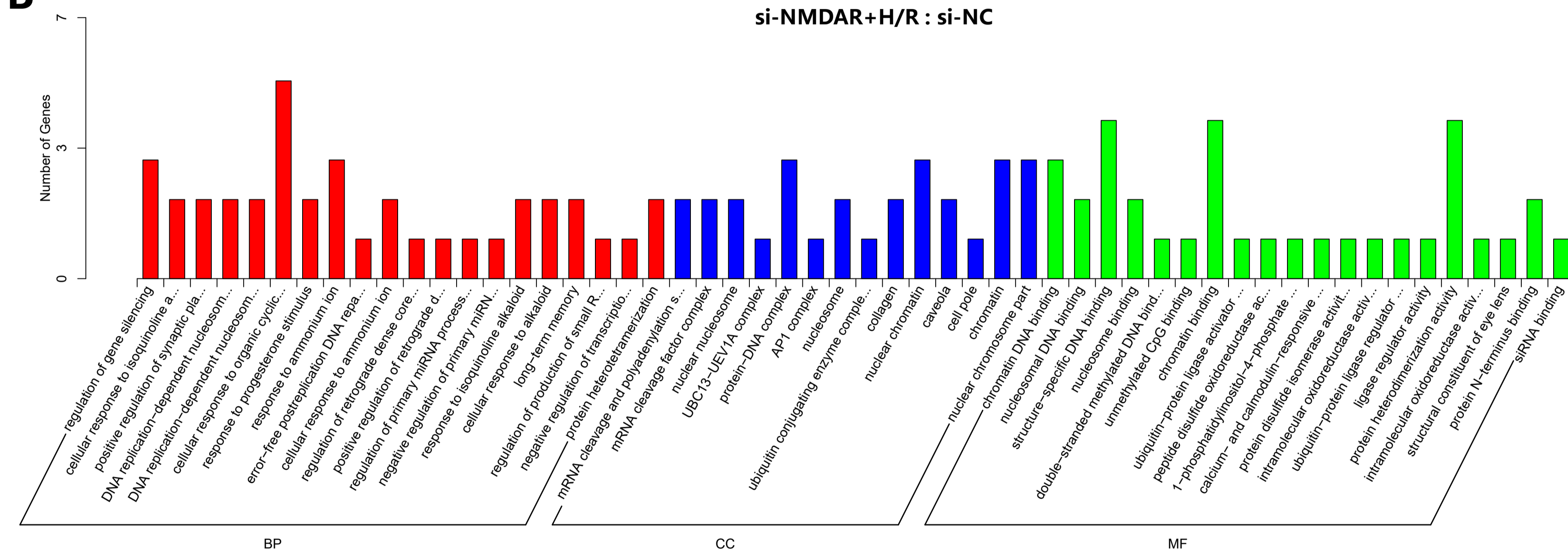

C

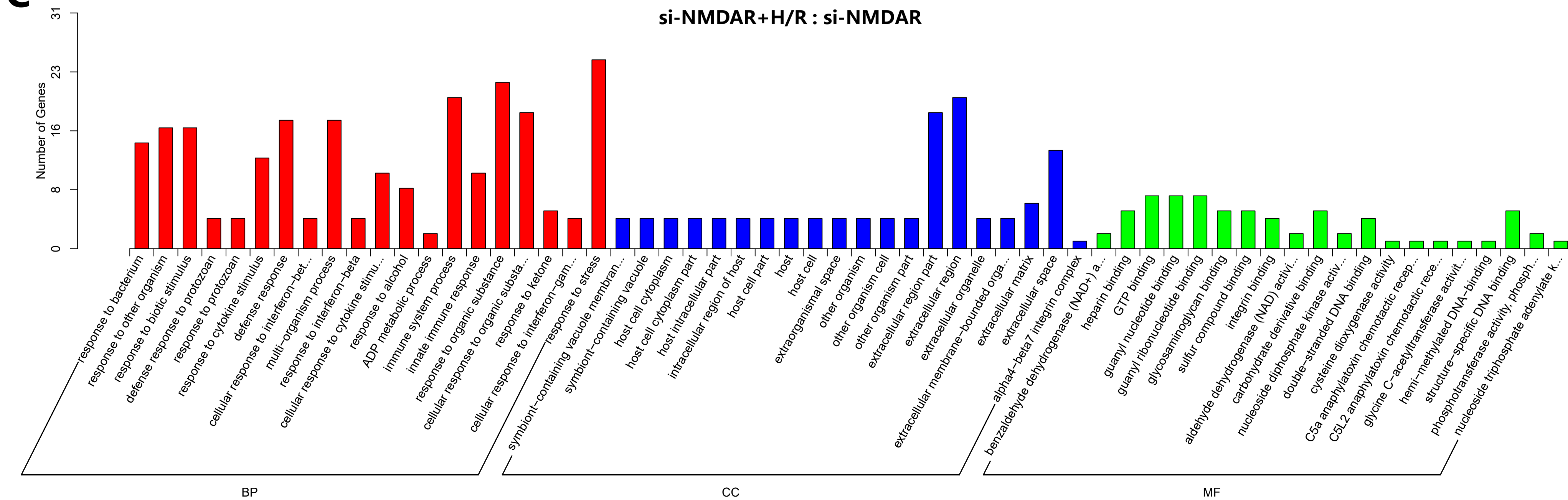

D

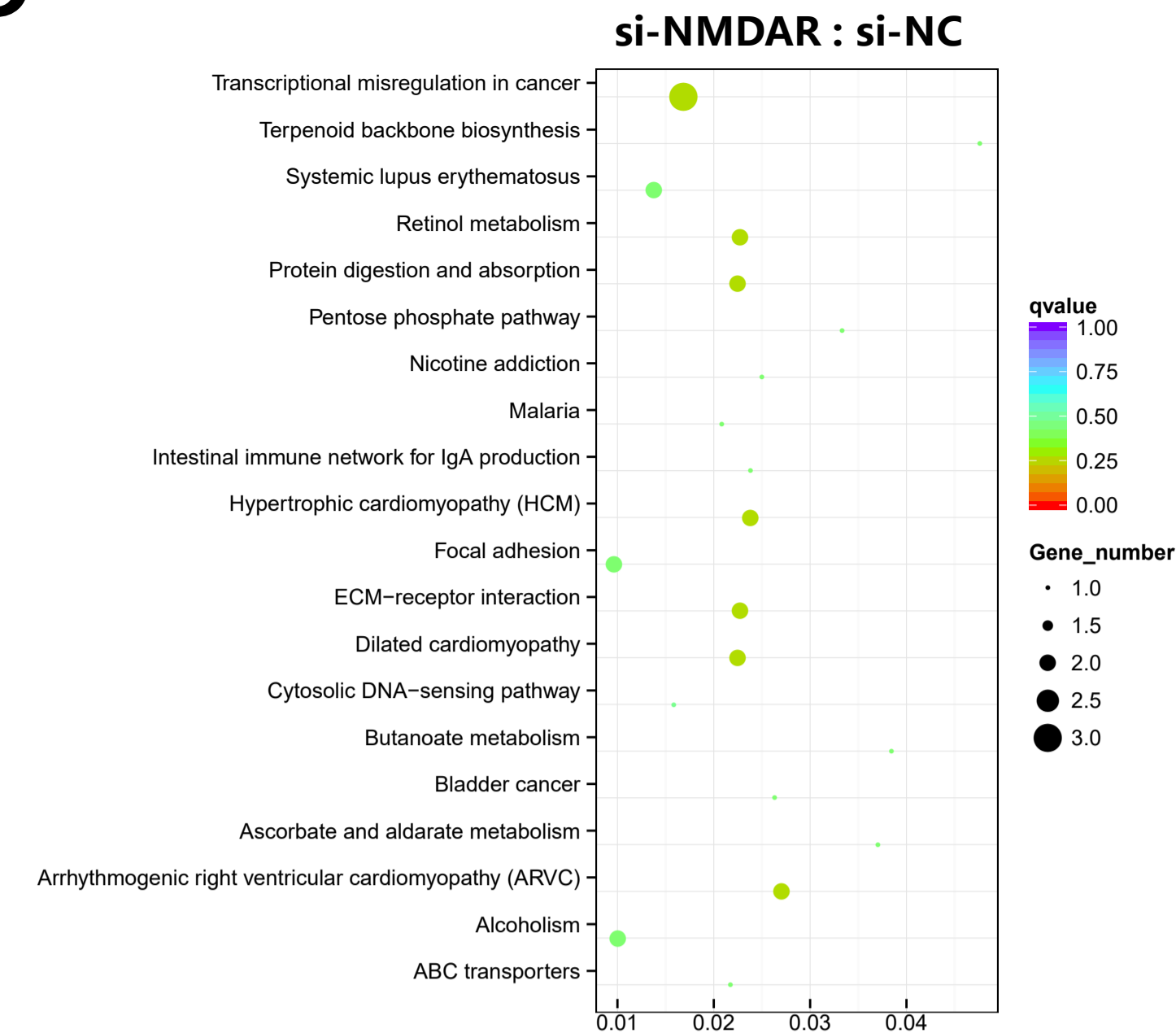

E

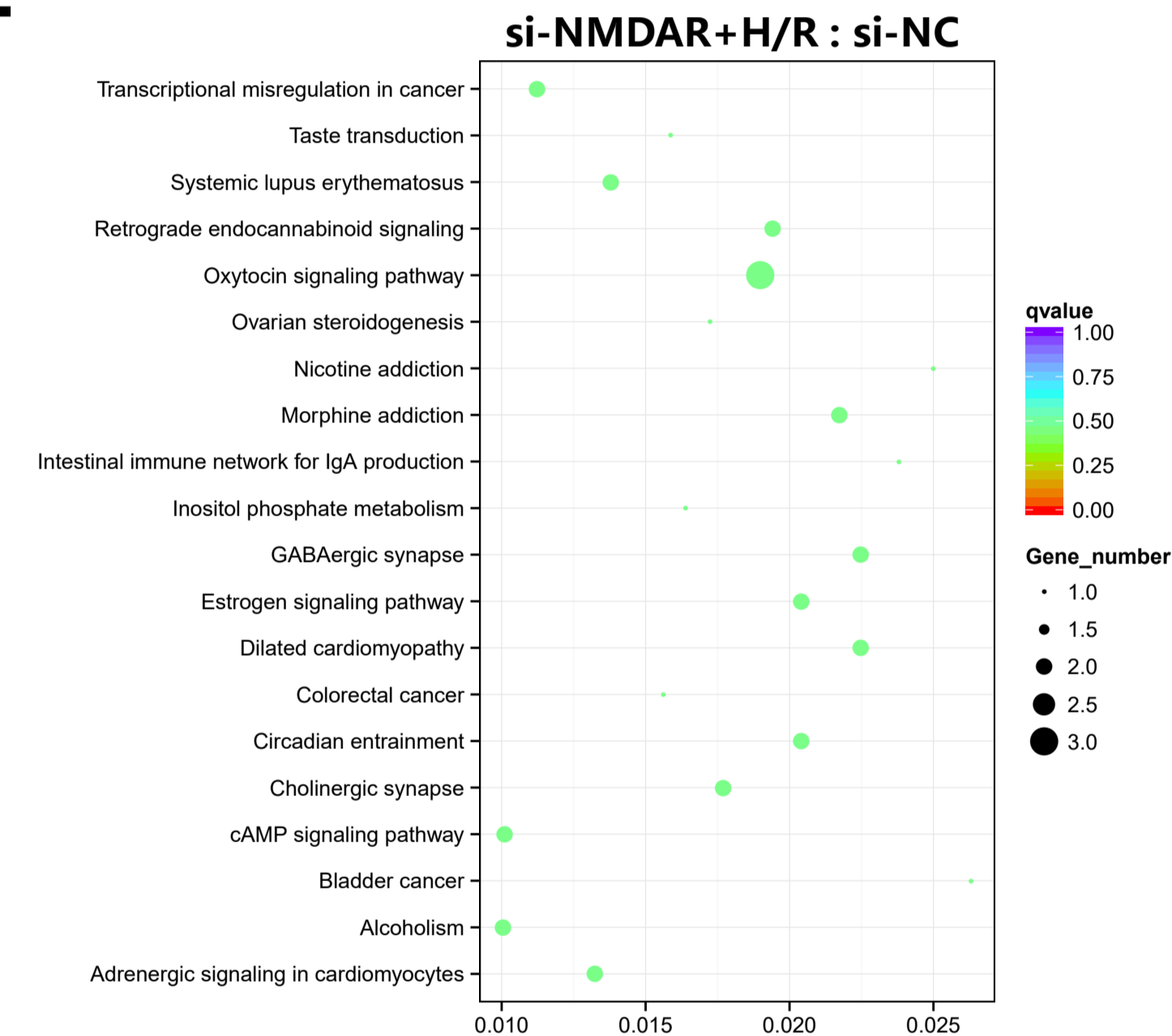

F

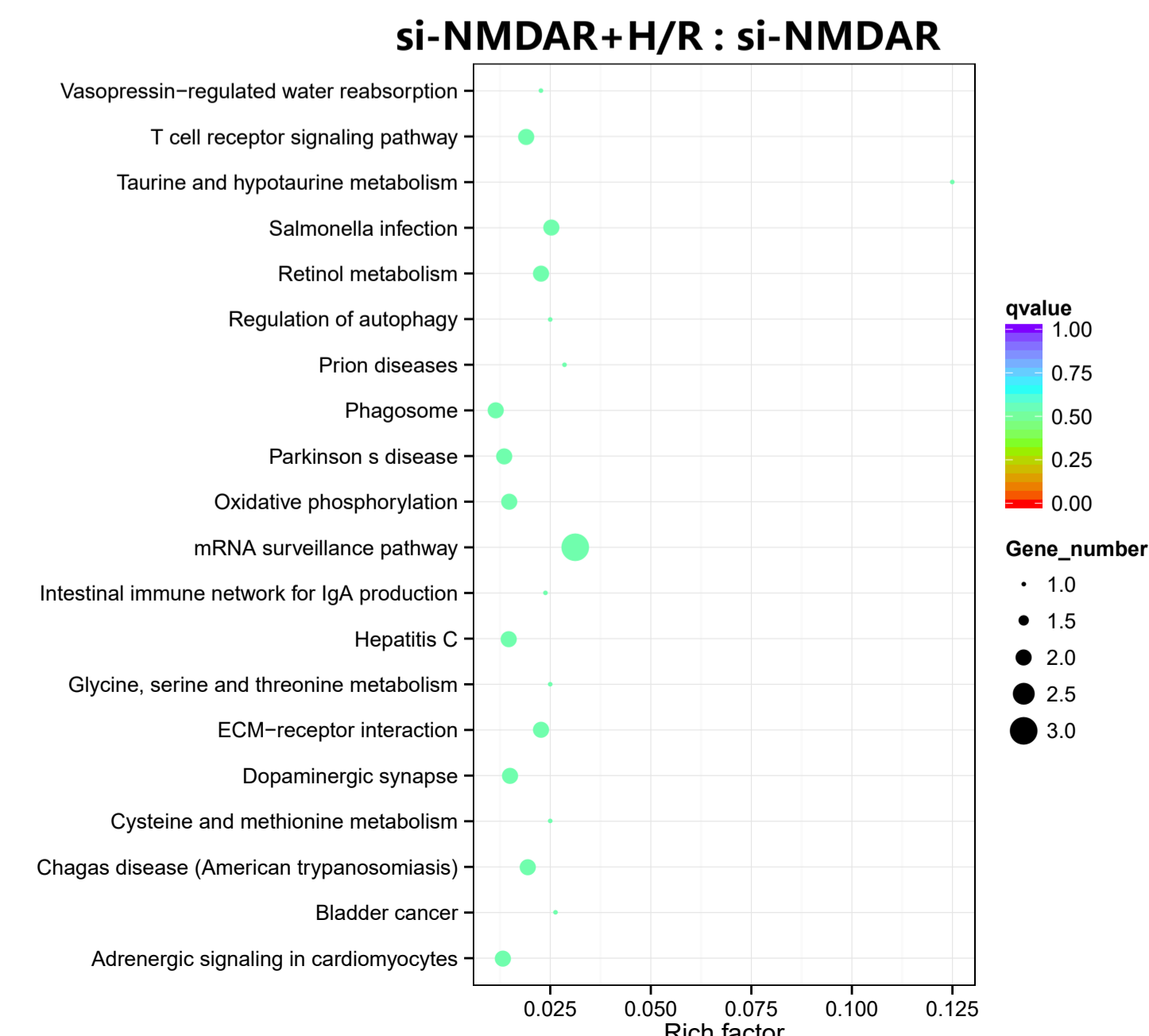

Supplement: SUPPLEMENTARY MATERIAL 7 — (A-C) GO analysis results showing the terms and proportions of mRNAs in the biological process, cellular component, and molecular function categories for each comparison. (D-F) Pathway analysis results showing the enrichment results for the differentially expressed mRNAs in each comparison. The vertical axis indicates the names of the top 20 pathways, the horizontal axis indicates the enrichment factor, the size of the dot represents the number of differentially expressed mRNAs in this pathway, and the color of the dot corresponds to the p-value. [file Data_Sheet_7.PDF]

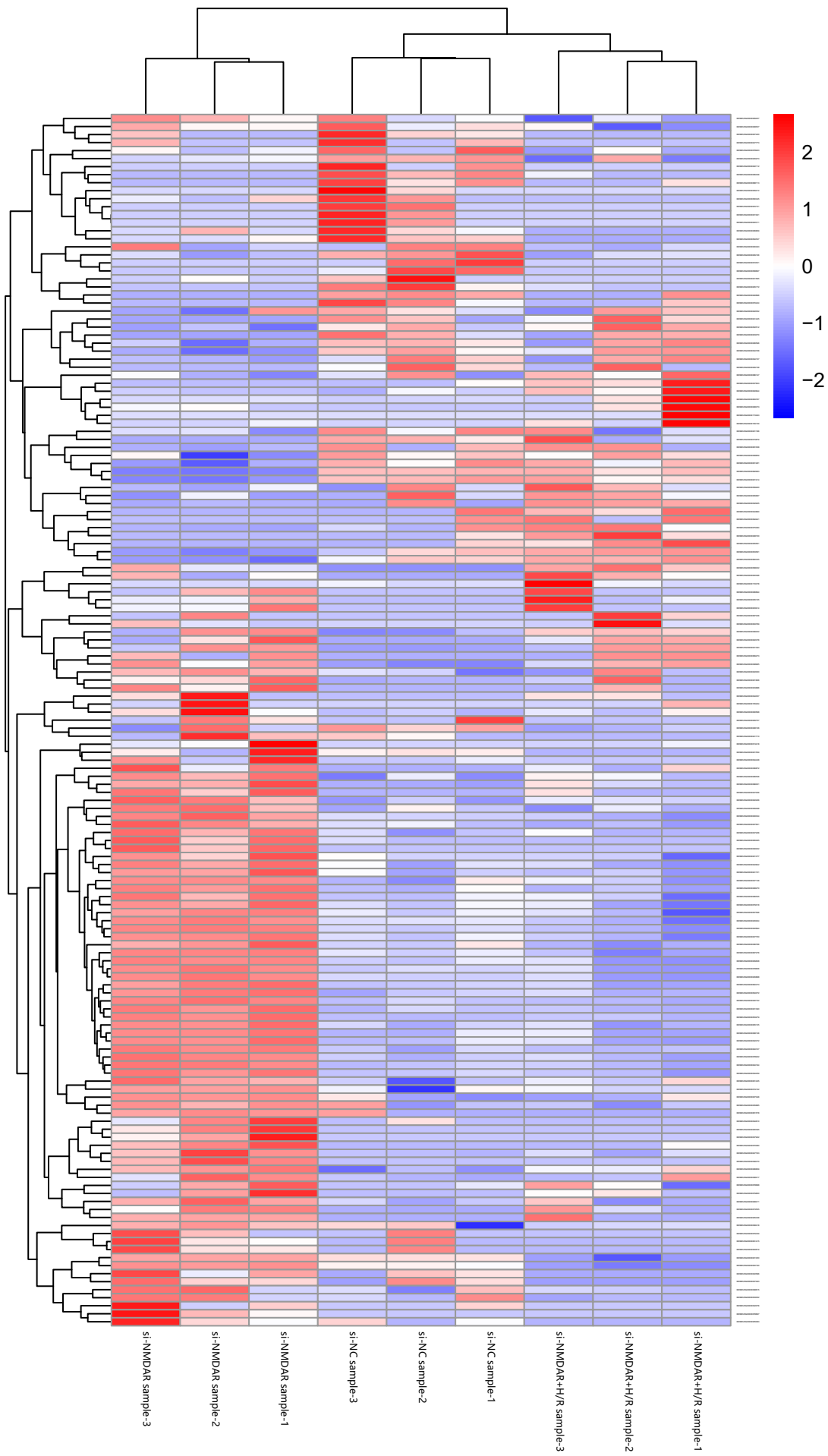

Supplement: SUPPLEMENTARY MATERIAL 9 — Cluster analysis heatmap showing the differentially expressed lncRNAs in each comparison. The horizontal axis indicates the samples, the vertical axis indicates the differentially expressed lncRNAs, the left side indicates the clustering of lncRNAs according to the degree of expression similarity, the right side indicates the names of lncRNAs, and the top indicates the clustering of the samples according to the similarity degree of the expression profile. The expression level gradually increases as the color changes from blue to red. [file Data_Sheet_9.PDF]

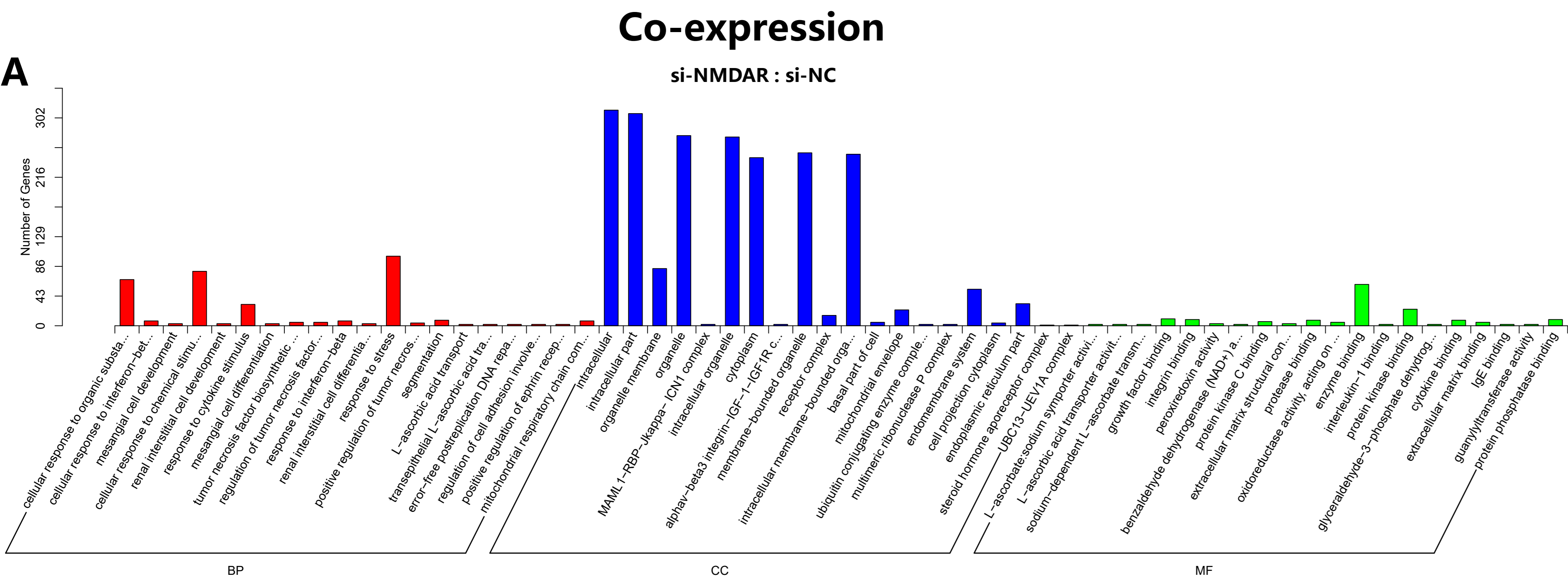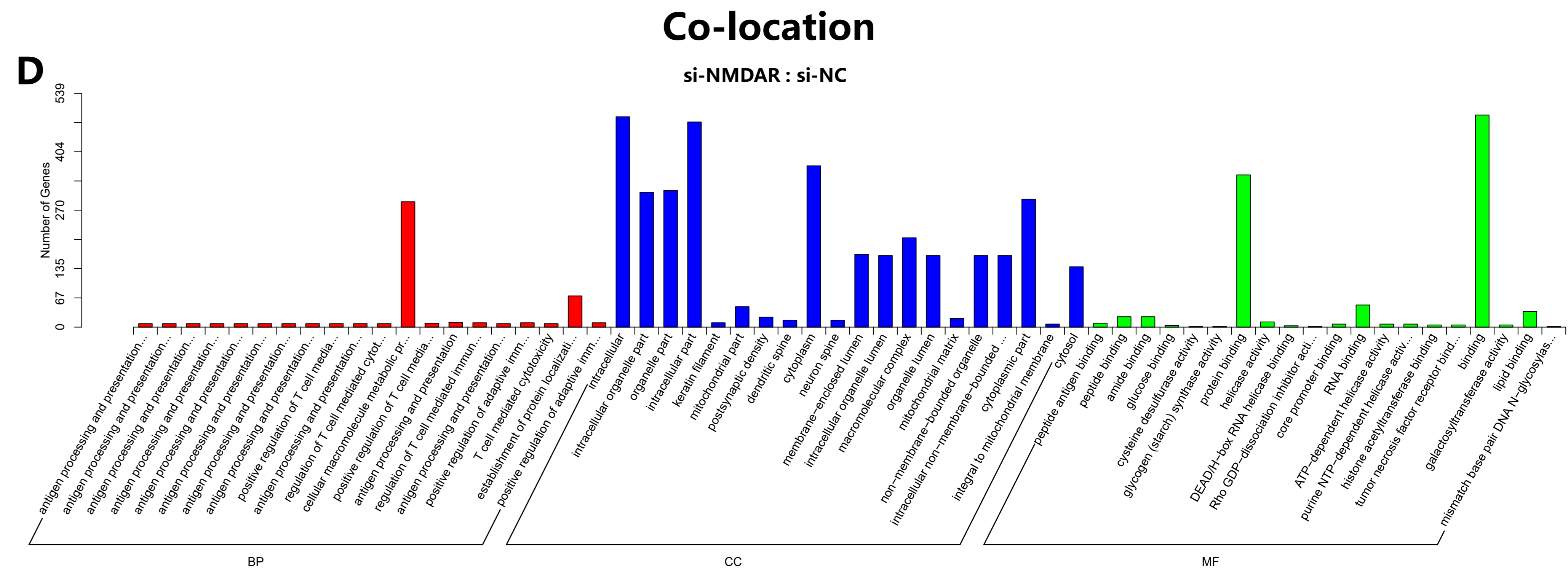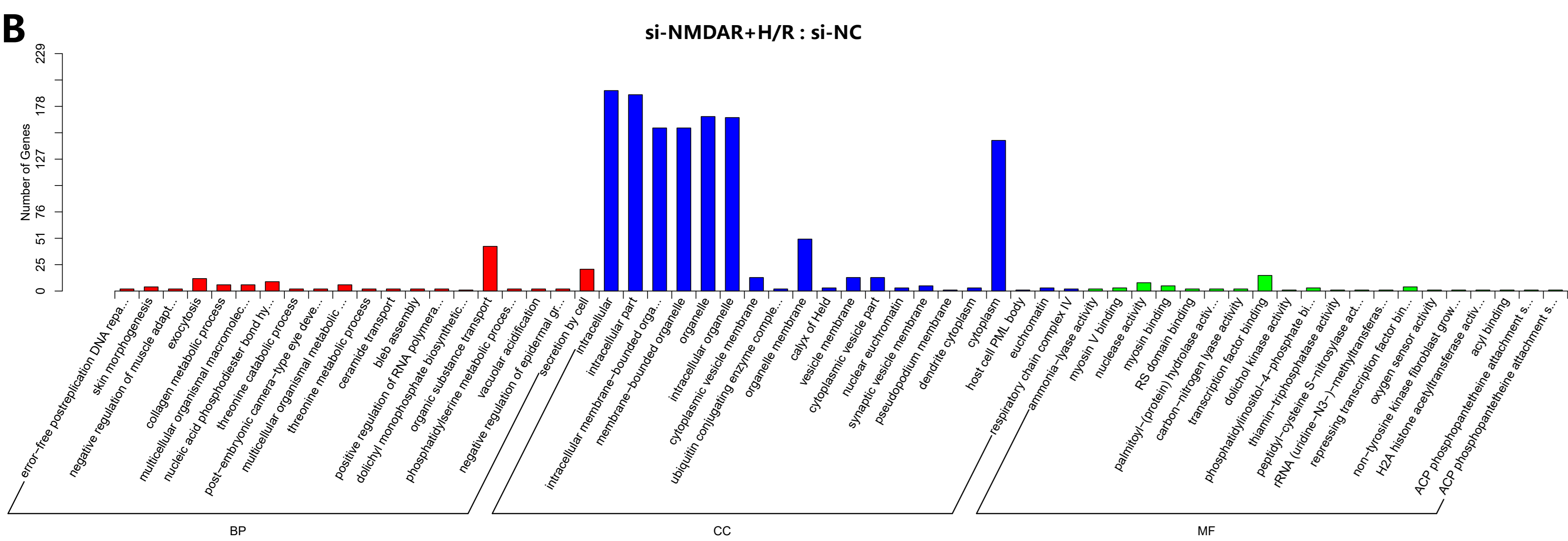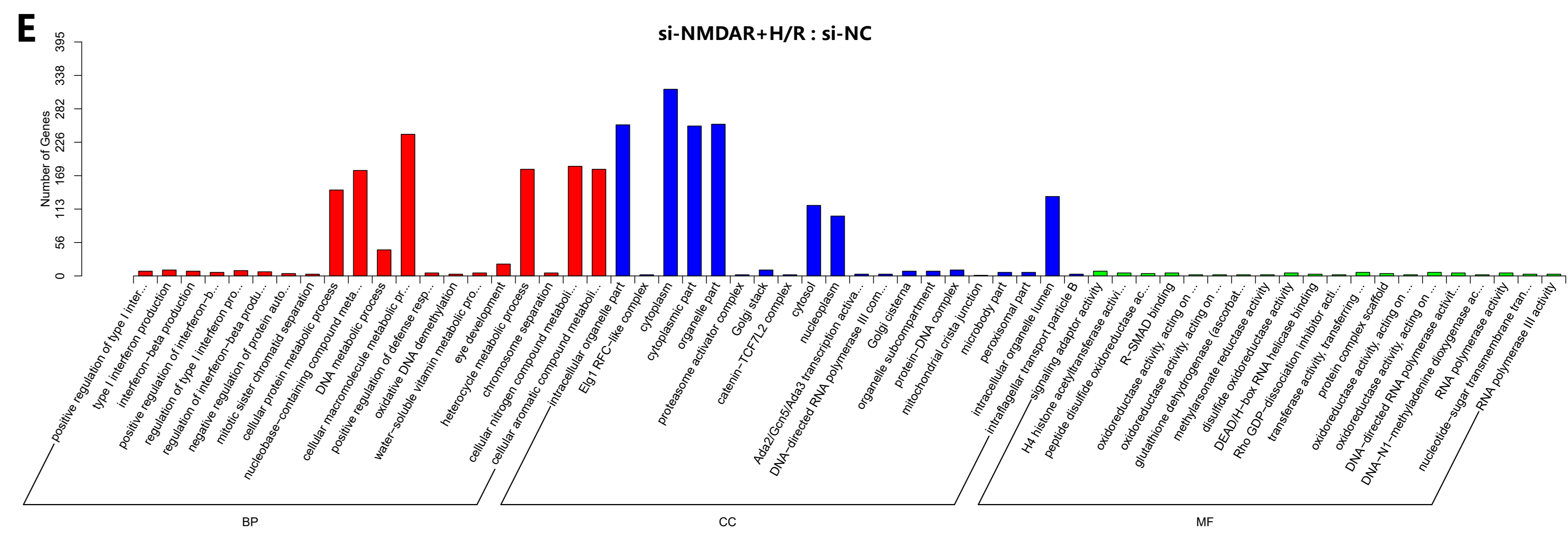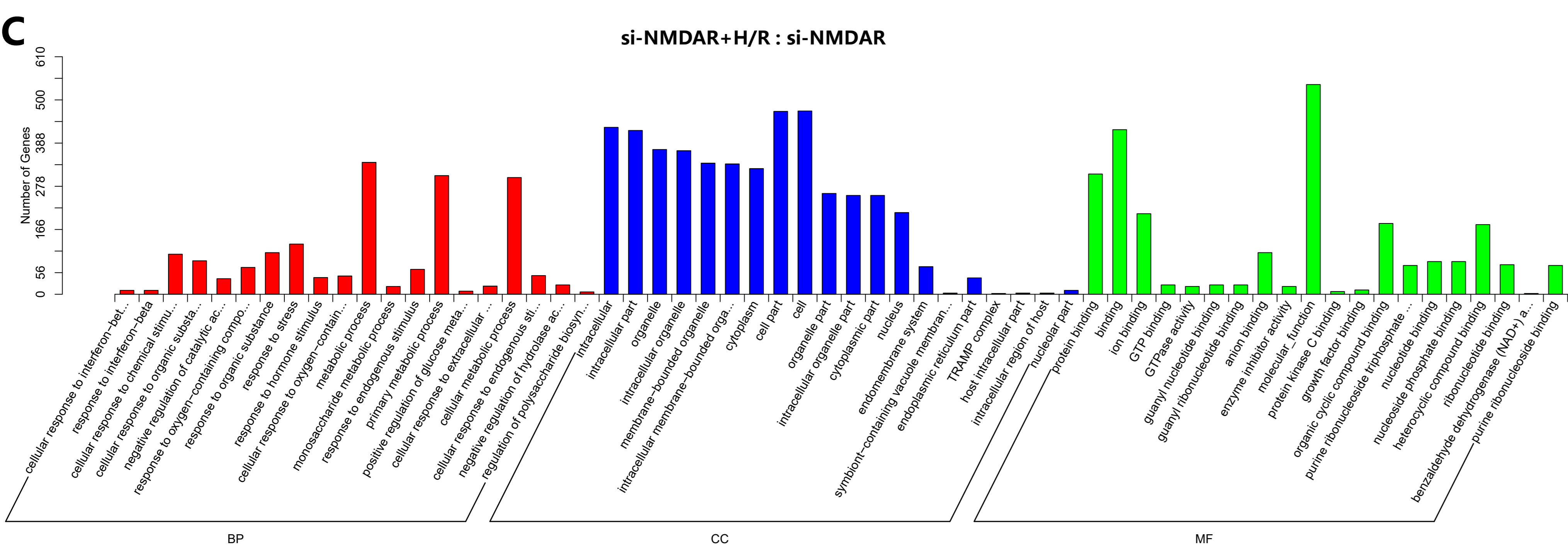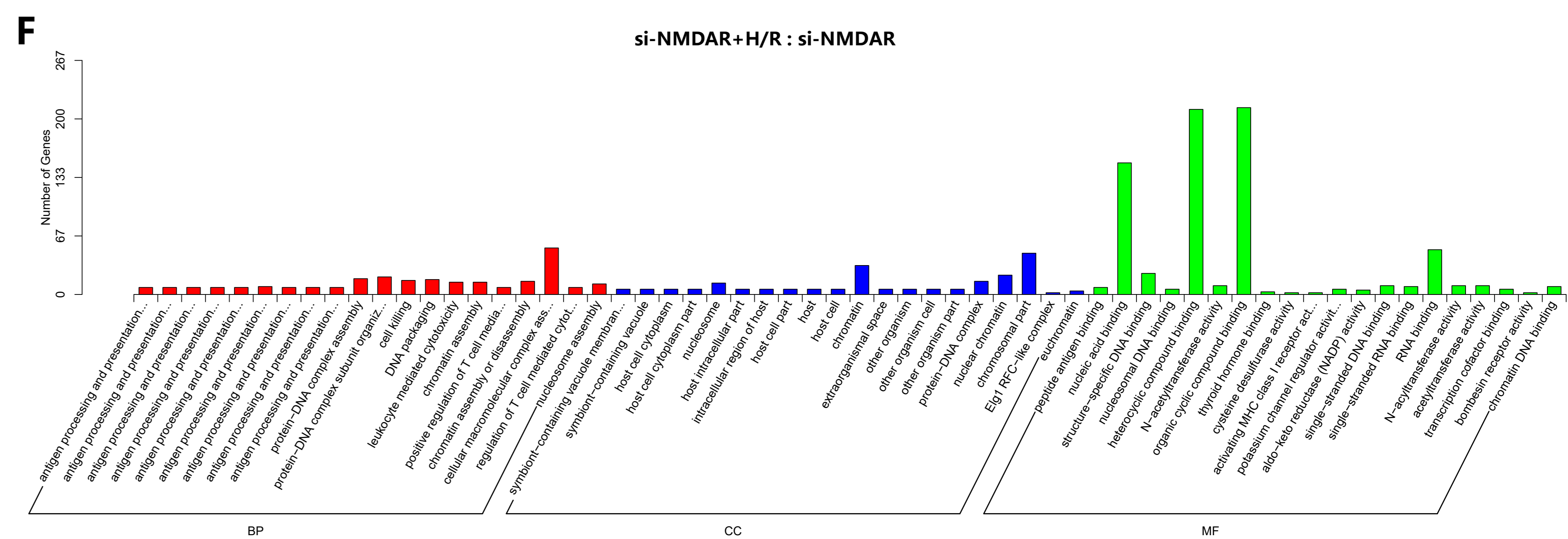

Supplement: SUPPLEMENTARY MATERIAL 10 — GO analysis results showing the terms and proportions of lncRNA target genes in the biological process, cellular component, and molecular function category for each comparison. (A-C) GO analysis results of target genes that were coexpressed with differentially expressed lncRNAs. (D-F) GO analysis results of target genes that were colocalized with differentially expressed lncRNAs. [file Data_Sheet_10.PDF]

si-NMDAR : si-NC

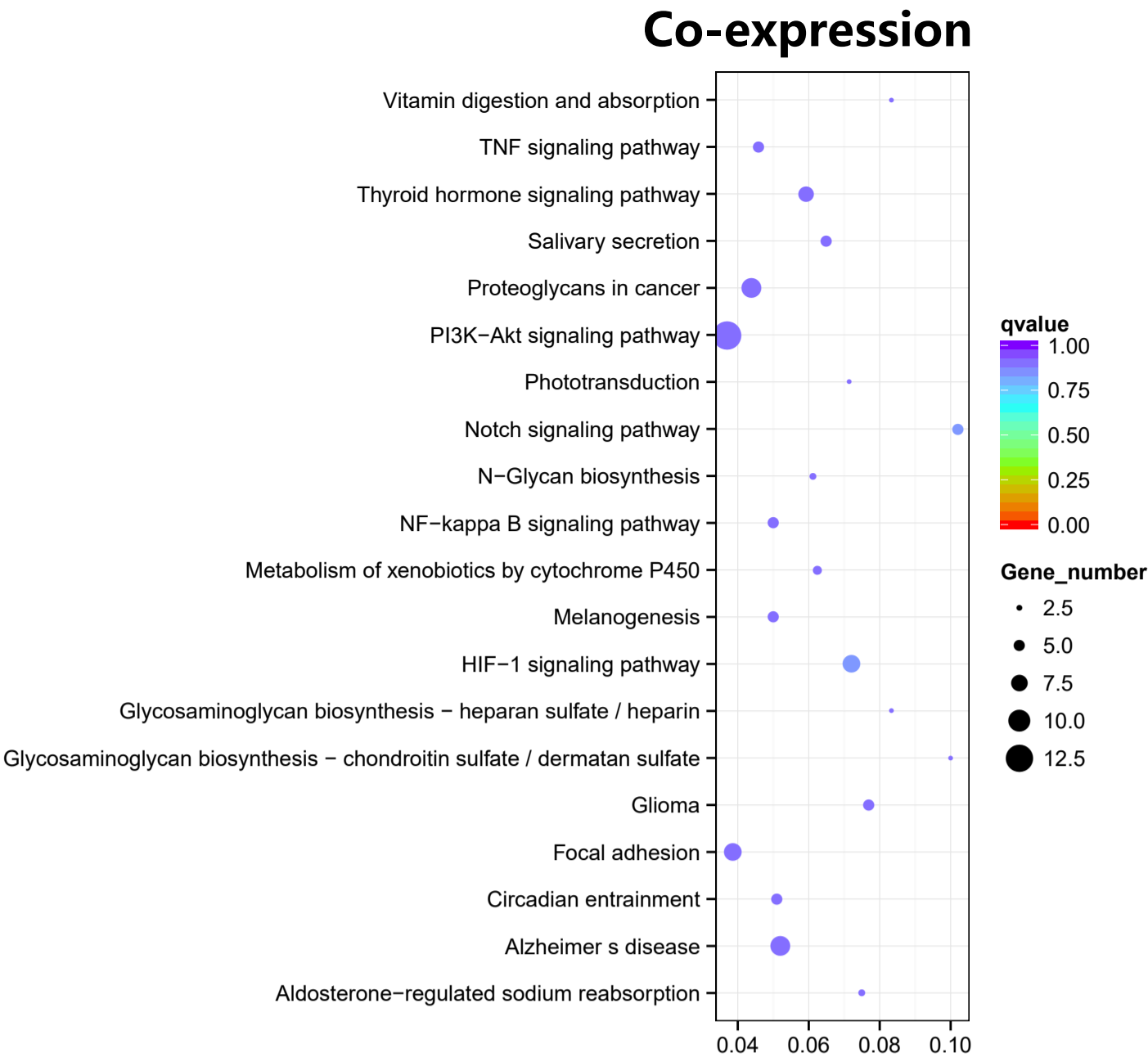

si-NMDAR+H/R : si-NC

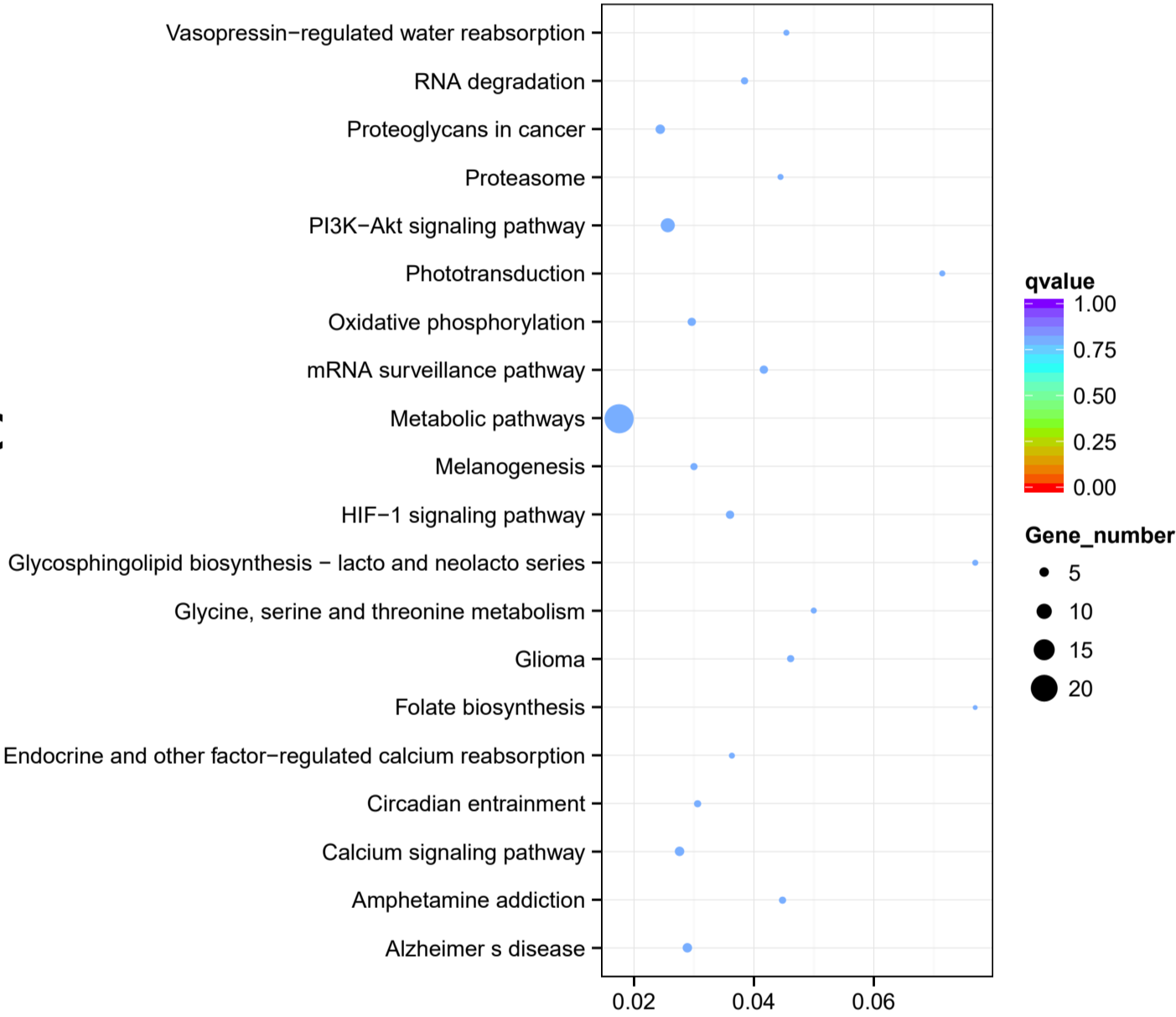

si-NMDAR+H/R : si-NMDAR

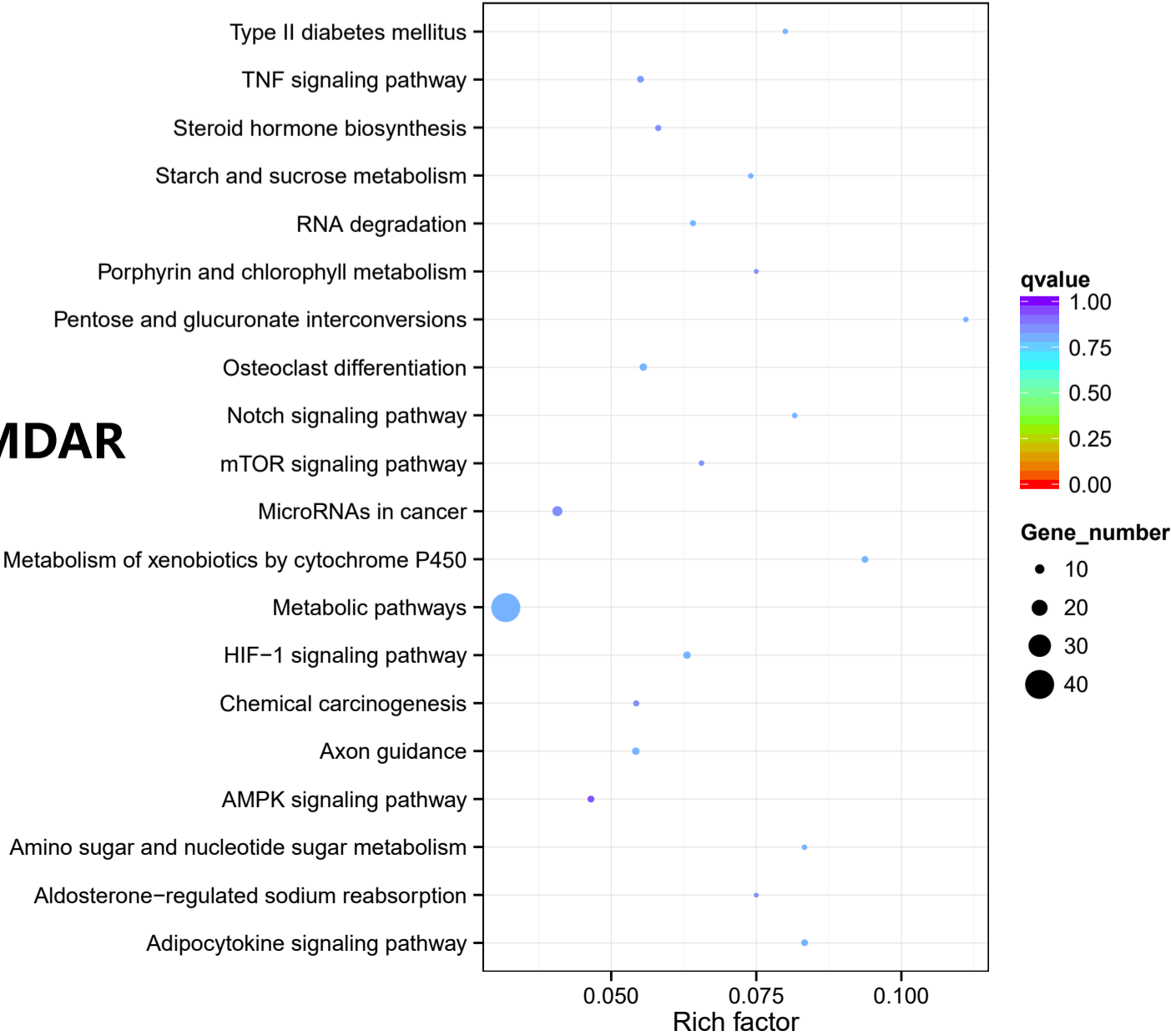

Co-location

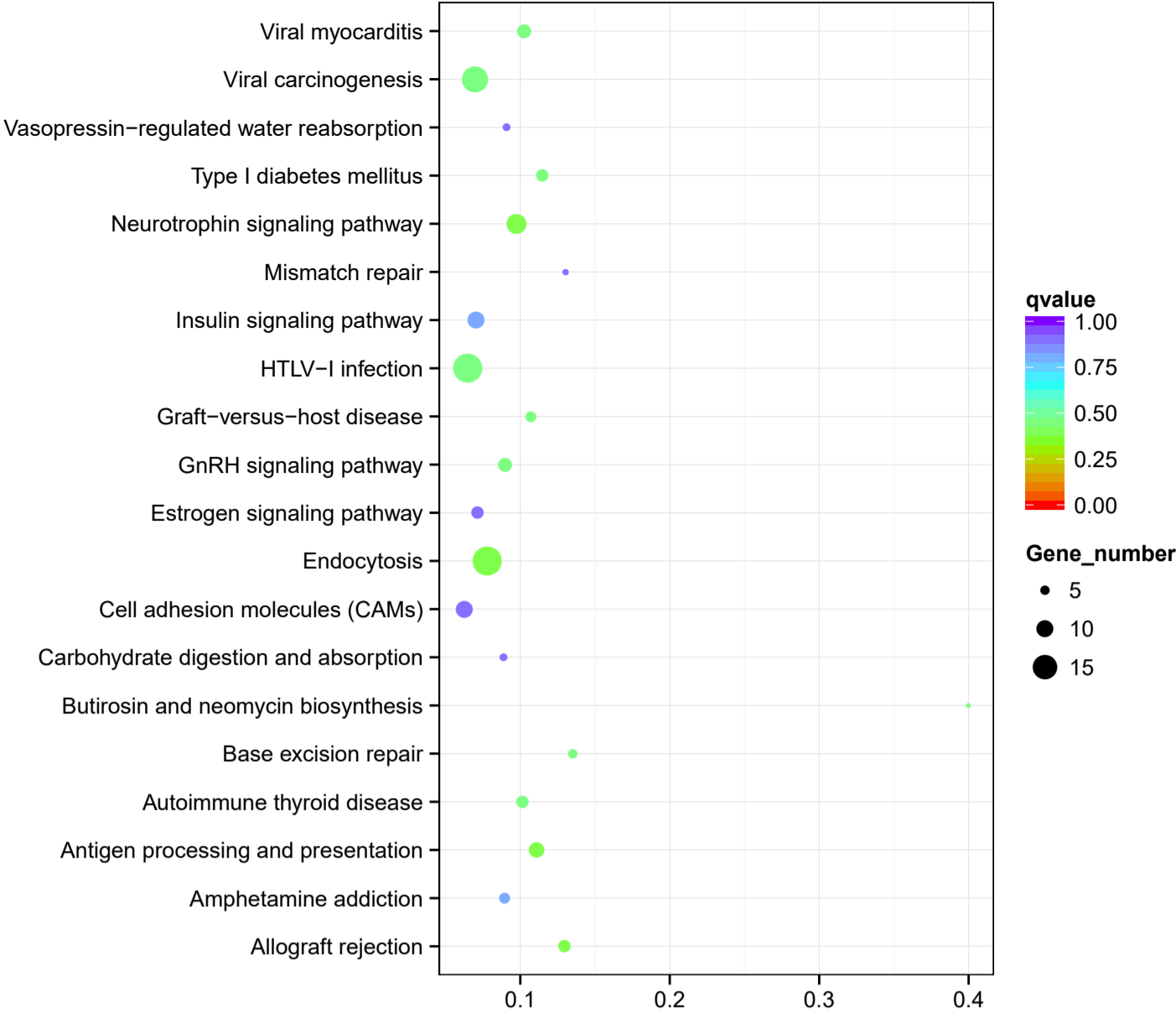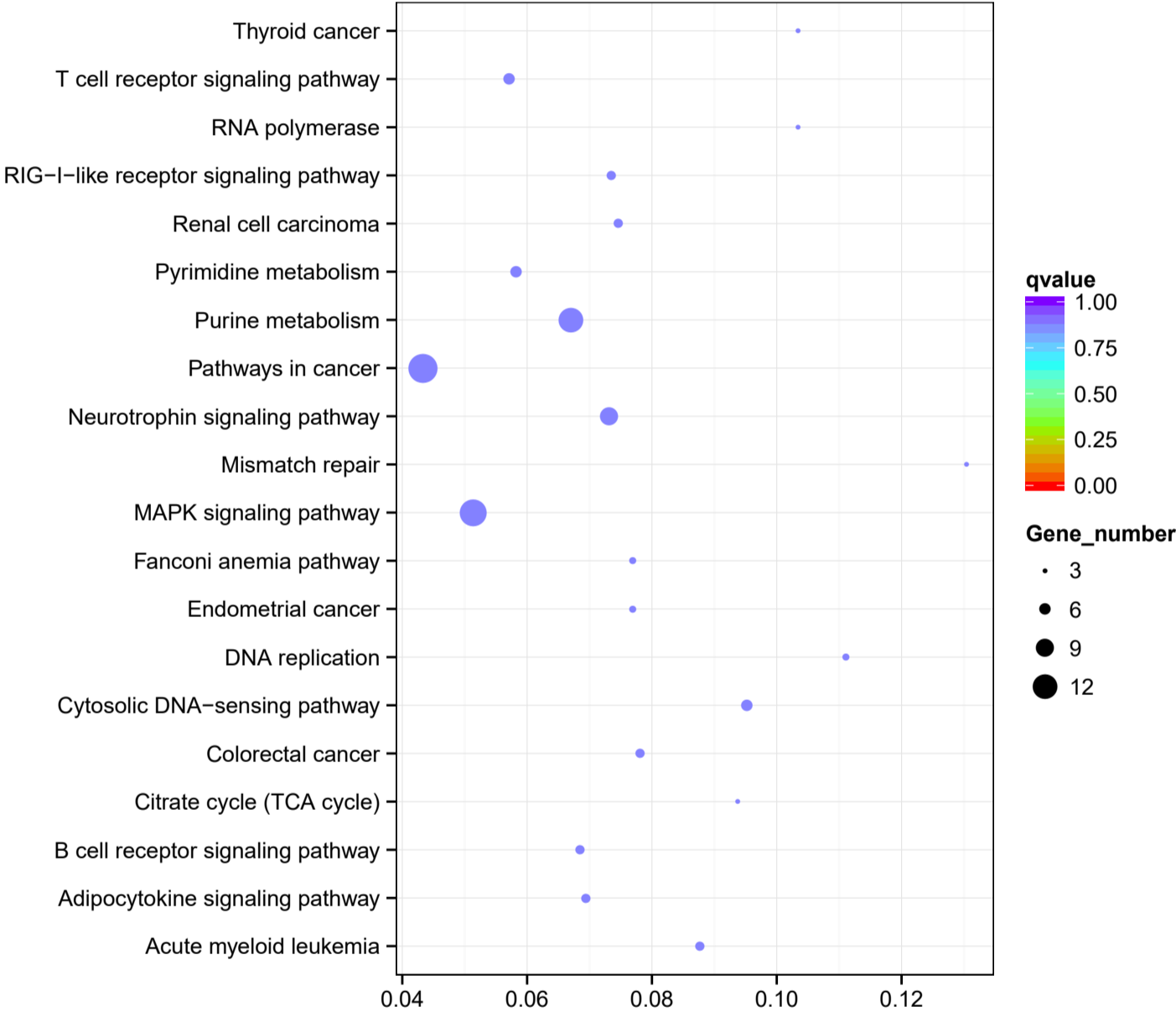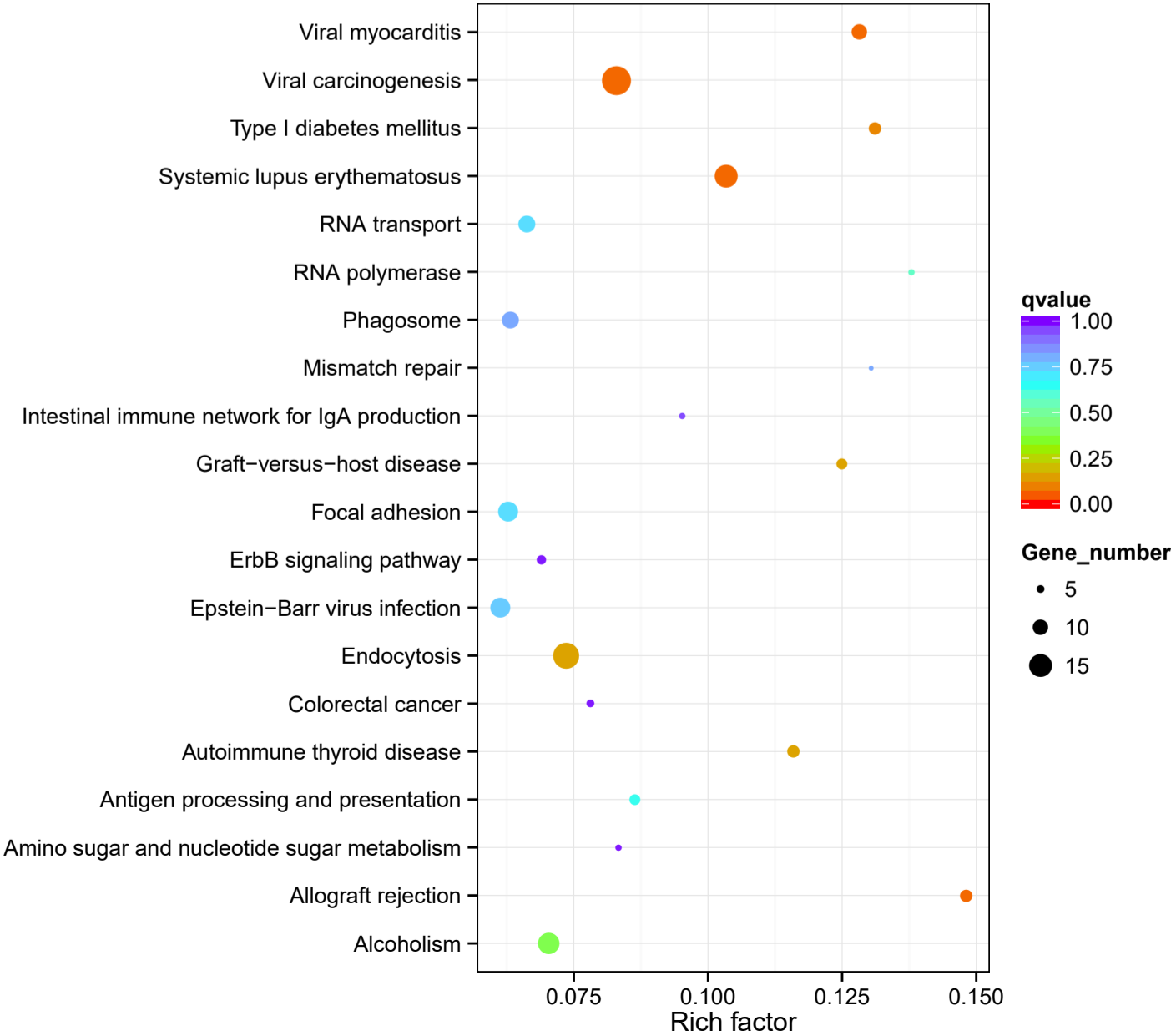

Supplement: SUPPLEMENTARY MATERIAL 12 — Pathway analysis showing the enrichment results of the differentially expressed lncRNAs in each comparison. The vertical axis indicates the names of the top 20 pathways, the horizontal axis indicates the enrichment factor, the size of the dot represents the number of differentially expressed lncRNAs in this pathway, and the color of the dot corresponds to the p-value. [file Data_Sheet_12.PDF]
