# Supplementary material for: Effects of N-methyl-D-aspartate receptor knockdown and hypoxia/reoxygenation injury on the neuronal proteome and transcriptome
Source: Front Mol Neurosci. 2022 Dec 15;15:1004375. doi: 10.3389/fnmol.2022.1004375 (PMC9799235; doi:10.3389/fnmol.2022.1004375)
Supplement: SUPPLEMENTARY MATERIAL 5 — Schematic diagram of the ribosome pathway and the proteins it contains. [file Data_Sheet_5.PDF]

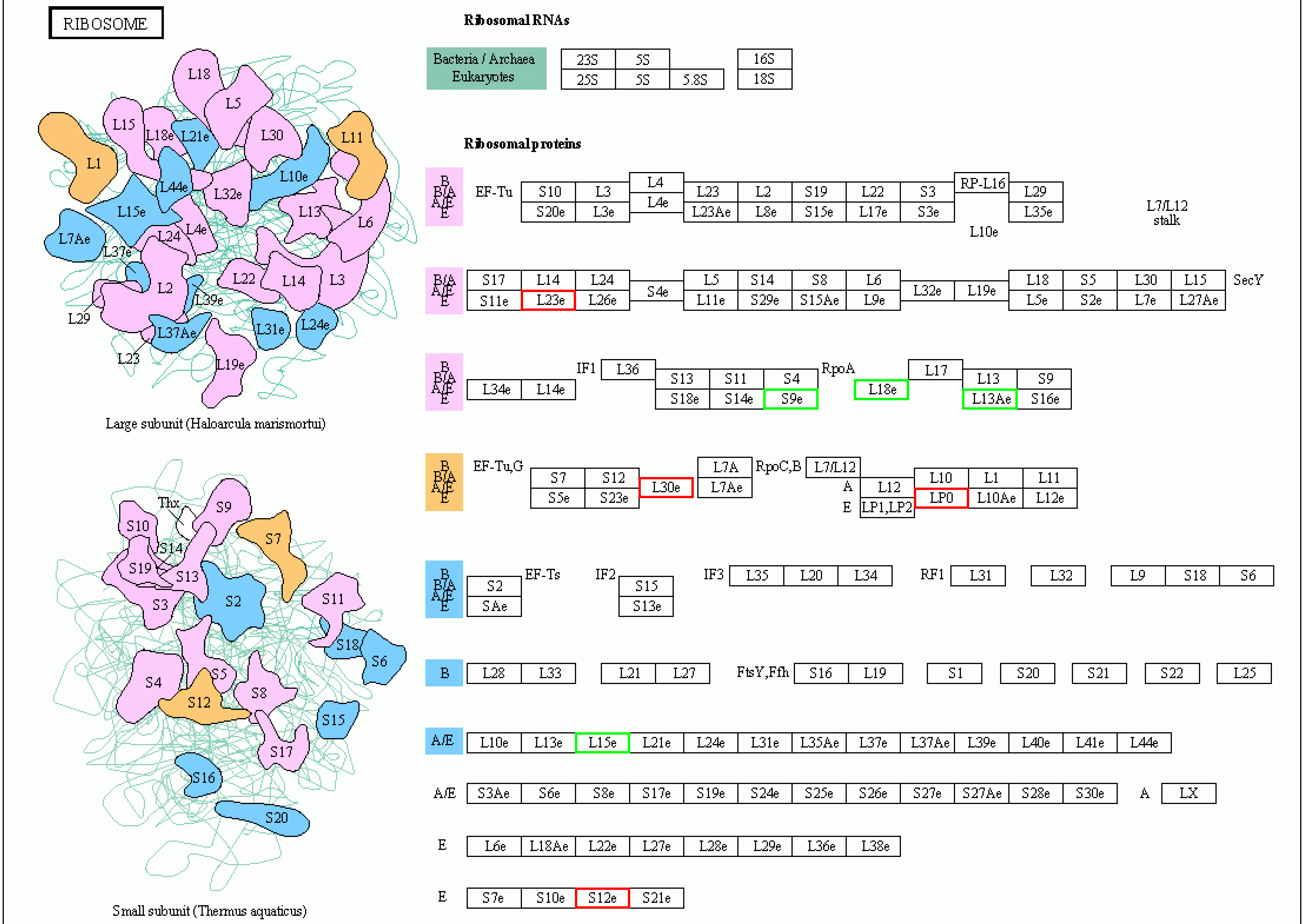
RIBOSOME
Ribosomal RNAs
Bacteria / Archaea
Eukaryotes
23S 5S 16S
25S 5S 5.8S 18S
Ribosomal proteins
B B/A A/E E
EF-Tu
S10 L3 L4 L23 L2 S19 L22 S3 RP-L16 L29
S20e L3e L4e L23Ae L8e S15e L17e S3e L10e L35e
L7/L12 stalk
B/A A/E E
S17 L14 L24 S4e L5 S14 S8 L6 L32e L19e L18 S5 L30 L15
S11e L23e L26e L11e S29e S15Ae L9e L5e S2e L7e L27Ae
SecY
B B/A A/E E
L34e L14e IF1 L36 S13 S11 S4 RpoA L18e L17 L13 S9
S18e S14e S9e L13Ae S16e
B B/A A/E E
EF-Tu,G
S7 S12 L30e L7A RpoC,B L7/L12 L10 L1 L11
S5e S23e L7Ae A L12 LP0 L10Ae L12e
E LP1,LP2
B B/A A/E E
S2 EF-Ts IF2 S15 IF3 L35 L20 L34 RF1 L31 L32 L9 S18 S6
SAe S13e
B
L28 L33 L21 L27 FtsY,Ffh S16 L19 S1 S20 S21 S22 L25
A/E
L10e L13e L15e L21e L24e L31e L35Ae L37e L37Ae L39e L40e L41e L44e
A/E
S3Ae S6e S8e S17e S19e S24e S25e S26e S27e S27Ae S28e S30e A LX
E
L6e L18Ae L22e L27e L28e L29e L36e L38e
E
S7e S10e S12e S21e
L18 L5 L15 L18e L21e L30 L11 L1 L10e L44e L32e L15e L13 L6 L7Ae L37e L24 L4e L22 L14 L3 L2 L39e L29 L37Ae L31e L24e L23 L19e
Large subunit (Haloarcula marismortui)
Thx S9 S10 S14 S7 S19 S13 S2 S3 S11 S18 S6 S4 S5 S8 S12 S15 S17 S16 S20
Small subunit (Thermus aquaticus)
