# Supplementary material for: Effects of N-methyl-D-aspartate receptor knockdown and hypoxia/reoxygenation injury on the neuronal proteome and transcriptome
Source: Front Mol Neurosci. 2022 Dec 15;15:1004375. doi: 10.3389/fnmol.2022.1004375 (PMC9799235; doi:10.3389/fnmol.2022.1004375)
Supplement: SUPPLEMENTARY MATERIAL 11 — DAG maps of GO enrichment of the differentially expressed lncRNA target genes in each comparison. Each node represents a GO term, the boxes represent the top 10 GO terms with regard to enrichment degree, and the color represents the enrichment degree (a darker color represents a higher enrichment degree). Each node displays the name and p-value of the term. [file Data_Sheet_11.PDF]

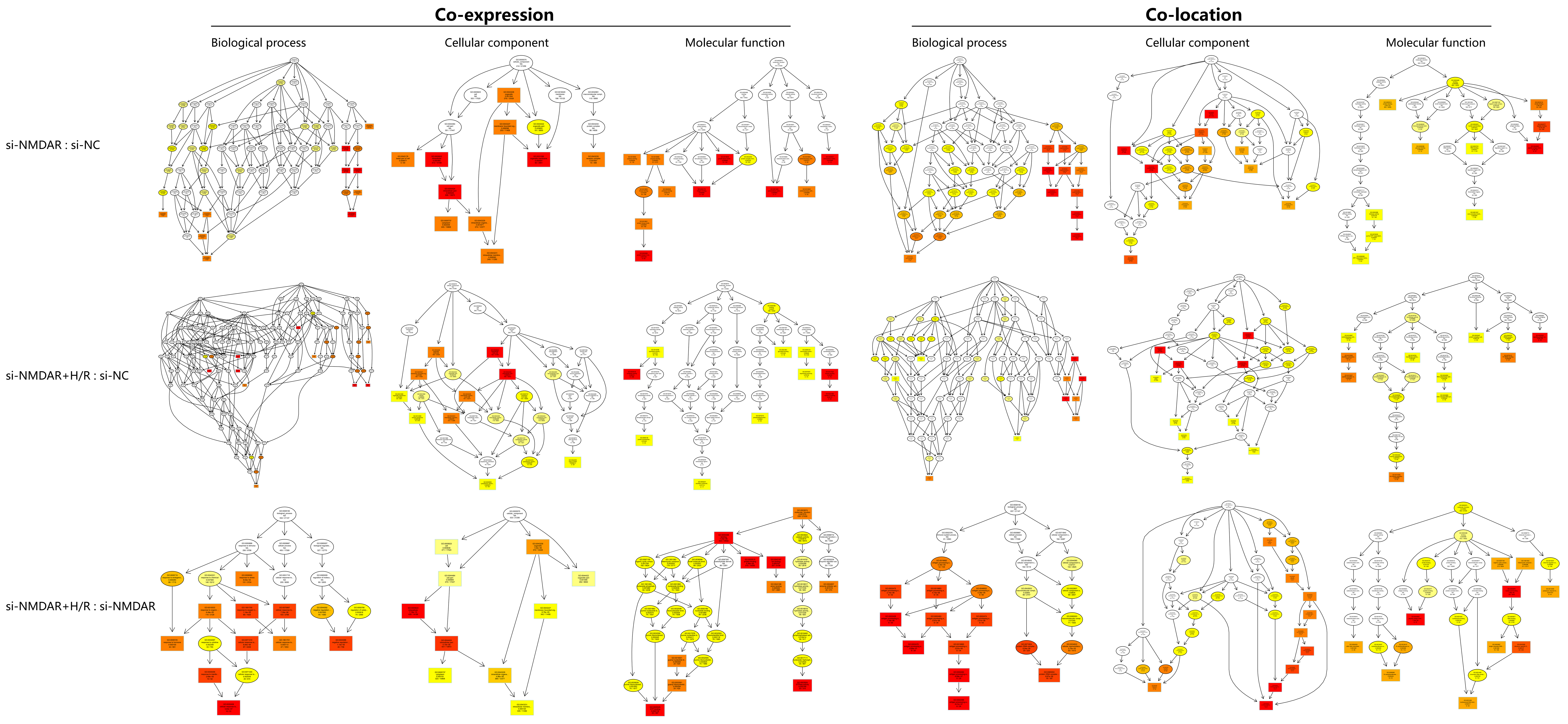

Co-expression
Co-location
Biological process
Cellular component
Molecular function
Biological process
Cellular component
Molecular function
si-NMDAR : si-NC
si-NMDAR+H/R : si-NC
si-NMDAR+H/R : si-NMDAR
