# Supplementary material for: Effects of N-methyl-D-aspartate receptor knockdown and hypoxia/reoxygenation injury on the neuronal proteome and transcriptome
Source: Front Mol Neurosci. 2022 Dec 15;15:1004375. doi: 10.3389/fnmol.2022.1004375 (PMC9799235; doi:10.3389/fnmol.2022.1004375)
Supplement: SUPPLEMENTARY MATERIAL 13 — KEGG pathways enriched for the target genes of lncRNA XLOC_159404, XLOC_031922, XLOC_161072, XLOC_065271 and Bank1. [file Data_Sheet_13.PDF]

Supplementary Material 7. The KEGG pathway enriched for the target genes of lncRNA XLOC_159404, XLOC_031922, XLOC_161072, XLOC_065271 and Bank1.

Co-expression

lncRNA Gene ID	mRNA Gene ID	mRNA Symbol	KEGG pathway item														
	ENSMUSG00000024048	Myl12a	Focal adhesion	Regulation of actin cytoskeleton	Tight junction	Platelet activation	Leukocyte transendothelial migration										
	ENSMUSG00000067562	Dmrtc1c1	-														
	ENSMUSG00000034164	Emid1	-														
	ENSMUSG00000042289	Hsd3b7	Metabolic pathways	Primary bile acid biosynthesis													
	ENSMUSG00000023047	Amhr2	Cytokine-cytokine receptor interaction	TGF-beta signaling pathway													
	ENSMUSG00000026463	Atp2b4	Salivary secretion	Calcium signaling pathway	Adrenergic signaling in cardiomyocytes	Pancreatic secretion	cAMP signaling pathway	cGMP-PKG signaling pathway									
XLOC_159404	ENSMUSG00000116024	Gm49527	-														
	ENSMUSG00000026923	Notch1	MicroRNAs in cancer	Notch signaling pathway	Thyroid hormone signaling pathway	Dorso-ventral axis formation	Prion diseases										
	ENSMUSG00000043298	Smco3	-														
	ENSMUSG00000081607	Gm15294	Metabolic pathways	HIF-1 signaling pathway	Alzheimer's disease	Glycolysis / Gluconeogenesis	Carbon metabolism	Biosynthesis of amino acids									
	ENSMUSG00000042485	Mustn1	-														
	ENSMUSG00000110040	Gm49369	Tuberculosis	Lysosome													
	ENSMUSG00000044702	Palb2	Fanconi anemia pathway														
	ENSMUSG00000024841	Eif1ad	-														
	ENSMUSG00000027797	Dclk1	-														
	ENSMUSG00000031015	Swap70	-														
	ENSMUSG00000090451	Gm6133	-														
	ENSMUSG00000039220	Ppp1r10	-														
	ENSMUSG00000047793	Sned1	-														
	ENSMUSG00000053178	Mterf1b	-														
	ENSMUSG00000024620	Pdgfrb	MicroRNAs in cancer	Calcium signaling pathway	Cytokine-cytokine receptor interaction	Focal adhesion	Regulation of actin cytoskeleton	PI3K-Akt signaling pathway	Glioma	Prostate cancer	Ras signaling pathway	Melanoma	Rap1 signaling pathway	HTLV-I infection	MAPK signaling pathway	Pathways in cancer	Gap junction
	ENSMUSG00000032806	Slc10a3	-														
	ENSMUSG00000054342	Kcnn4	Salivary secretion	Insulin secretion	Protein digestion and absorption												
XLOC_031922	ENSMUSG00000056204	Pgpep1	-														
	ENSMUSG00000031146	Plp2	-														
	ENSMUSG00000023022	Lima1	-														
	ENSMUSG00000030111	A2m	Complement and coagulation cascades														
	ENSMUSG00000028268	Gbp3	-														
	ENSMUSG00000050914	Ankrd37	-														
	ENSMUSG00000011958	Bnip2	-														
	ENSMUSG00000038925	E330034G19Rik	-														
	ENSMUSG00000037242	Clic4	-														
	ENSMUSG00000028463	Car9	Nitrogen metabolism														
	ENSMUSG00000028789	Azin2	Metabolic pathways	Arginine and proline metabolism													
XLOC_161072	ENSMUSG00000037617	Spag1	-														
	ENSMUSG00000018678	Sp2	-														
	ENSMUSG00000034157	Cipc	-														
	ENSMUSG00000042515	Pwwp3b	-														
	ENSMUSG00000022744	Cldnd1	-														
XLOC_065271	ENSMUSG00000110104	Gm45717	-														
	ENSMUSG00000069184	Zfp72	-														
	ENSMUSG00000049232	Tigd2	-														
	ENSMUSG00000064289	Tank	-														
	ENSMUSG00000050786	Ccdc126	-														
Bank1	None	None	-														

Co-location

lncRNA Gene ID	mRNA Gene ID	mRNA Symbol	KEGG pathway item														
	ENSMUSG00000031132	Cd40lg	Systemic lupus erythematosus	Allograft rejection	Cytokine-cytokine receptor interaction	Autoimmune thyroid disease	Cell adhesion molecules (CAMs)	T cell receptor signaling pathwa	Viral myocarditis	NF-kappa B signaling pathway		Asthma	Intestinal immune network for IgA production		Primary immunodeficiency	Malaria	Toxoplasmosis
	ENSMUSG00000031133	Arhgef6	Regulation of actin cytoskeleton	Pancreatic cancer													
XLOC_159404	ENSMUSG00000031130	Brs3	Neuroactive ligand-receptor interaction														
	ENSMUSG00000031131	Vgll1	-														
	ENSMUSG00000053852	Adgrg4	-														
	ENSMUSG00000067873	Htatsf1	-														
	ENSMUSG00000060807	Serpina6	-														
	ENSMUSG00000079015	Serpina1c	Complement and coagulation cascades														
	ENSMUSG00000066366	Serpina1a	-														
XLOC_031922	ENSMUSG00000071178	Serpina1b	-														
	ENSMUSG00000071179	Serpina16	-														
	ENSMUSG00000071177	Serpina1d	Complement and coagulation cascades														
	ENSMUSG00000021081	Serpina1f	-														
XLOC_161072	ENSMUSG00000067441	H2afb1	Systemic lupus erythematosus	Alcoholism													
XLOC_065271	ENSMUSG00000035842	Ddx11	-														
	ENSMUSG00000052105	Mtcl1	-														
Bank1	ENSMUSG00000037922	Bank1	-														
