# Supplementary material for: Effects of N-methyl-D-aspartate receptor knockdown and hypoxia/reoxygenation injury on the neuronal proteome and transcriptome
Source: Front Mol Neurosci. 2022 Dec 15;15:1004375. doi: 10.3389/fnmol.2022.1004375 (PMC9799235; doi:10.3389/fnmol.2022.1004375)
Supplement: SUPPLEMENTARY MATERIAL 14 — All proteins, mRNAs and lncRNAs that were up-/downregulated in each comparison. [file Data_Sheet_14.PDF]

Supplementary Material 8. All proteins, mRNAs and lncRNAs up/down-regulated in each comparison group.

Rank	Protein			mRNA			lncRNA		
	**si-NMDAR : si-NC**	**si-NMDAR HR : si-NC**	**si-NMDAR HR : si-NMDAR**	**si-NMDAR : si-NC**	**si-NMDAR HR : si-NC**	**si-NMDAR HR : si-NMDAR**	**si-NMDAR : si-NC**	**si-NMDAR HR : si-NC**	**si-NMDAR HR : si-NMDAR**
1	Tfrc	Rpl6	Rps9	Gm43738	Astn2	Gm49333	XLOC_029864	XLOC_029864	XLOC_161072
2	Ddx5	Tfrc	Rpl6	Slc22a14	Gm49333	Gm15093	Gm49936	XLOC_029876	XLOC_028364
3	Canx	Mybbp1a	H1-4	AC125149.3	Gm44973	Gcat	XLOC_159404	Gm49936	XLOC_031910
4	Hist2h2aa1	Ddx5	Rpl18	Astn2	Hist1h3e	Sh2d4b	XLOC_005151	XLOC_003058	XLOC_005156
5	Shmt2	Anxa2	H1f0	Bank1	Adtrp	Cfap57	XLOC_004709	XLOC_028364	Gm8739
6	Lrpprc	Lrrc59	Rpl14	Morn5	Kif28	Mmrn2	XLOC_155543	XLOC_031910	XLOC_111180
7	Plec	H1f5	Rps8	B4galnt2	Adcy8	Spink5	XLOC_003058	XLOC_005156	XLOC_135584
8	Slc25a5	Shmt2	Rpl15	Pcp4l1	Tnfrsf17	Gm1123	Gm11508	Gm8739	XLOC_058931
9	Pdia3	Slc25a5	H1f5	Myrfl	S100a7a	S100a7a	XLOC_031922	XLOC_111180	Gm41496
10	Cs	H1-4	Rpl24	Hist1h3e	Hist2h3c1	Hist2h3c2	Gm45193	XLOC_004709	XLOC_065271
11	Tmtc3	Plec	Rpl13a	Gm4724	Mecp2	Wipf1	Bank1	Gm45193	XLOC_078536
12	Anxa5	Lrpprc	Ndufa13	Abcc12	Ube2l3	Zfp989	Gm16685	XLOC_063215	Gm49539
13	Npm1	Ddx21	H1-1	Smoc2	Pdia5	Rnaset2a	XLOC_057817	XLOC_048326	XLOC_050059
14	Mybbp1a	Brix1	Anxa2	Adtrp	Fam122b	Atg12	XLOC_040327	Gm11508	XLOC_080942
15	Hspd1	Kpna2	Brix1	Acsm4		Pdia5	XLOC_071856	Gm49761	XLOC_112628
16	Hmga2	Pwp2	Cyp51a1	Mep1a		Ppp2r2a	XLOC_091837	Adtrp	Gm40884
17	Hspa9	Cebpz	Pwp2	Itk		4930453N24Rik	6530401F13Rik	XLOC_162785	Gm28055
18	Hspe1	Hspa9	Uncharacterized protein KIAA1143 homolog	Egln3		Srrm1	4930556G22Rik	Gm48294	XLOC_122169
19	Rpf2	Slc25a3	Rab7	Padi3		Zfand2a	Gm4724	Gm45303	XLOC_071178
20	Ndufa4	Alb	Macroh2a1	Adamts9		Itgb7	Gm11264	Adcy8	Gm16043
21	Ahnak	Hspe1	Hnrnpa1	Gm4951		mt-Nd2	Smoc2	XLOC_149580	XLOC_080523
22	Lrrc59	Cs	Eef1g	Tlcd2		Nav2	Adtrp	S100a7a	Gm43569
23	Mydgf	Canx	Rps28	Nid2			Gm20661	Gm43138	Sh2d4b
24	Snu13	Rplp0	Nop58	Mecp2			2610027K06Rik	Mecp2	S100a7a
25	Kpna2	Hist2h2aa1	Mnda	Hnrnpd			Mep1a	Ube2l3	Wipf1
26	Pdia6	Wdr43	Rps15a	Ube2l3			Itk	XLOC_141938	XLOC_095775
27	Syncrip	Hsd17b12	Nup155	Aldh1a1			Adamts9	Pdia5	Zfp989
28	Grpel1	Phb	Rps3	Mup2			XLOC_073225	Gm36107	Atg12
29	Rpn1	Rab1b	Uxs1	Iigp1			Gm13431	Fam122b	Pdia5
30	Erh	Rpn1	Ptp4a2	2610528A11Rik			Tlcd2		Ppp2r2a
31	Fkbp2	Ptcd3	Slain2	Tgtp1			Nid2		4930453N24Rik
32	Wdr43	Rpl30	Eif2s2	Ogn			Gm15614		Srrm1
33	Timm8a1	Ssr3	Cebpz	Aldh1a7			Mecp2		Zfand2a
34	Envelope protein	Snrnp200		Fam122b			Hnrnpd		Itgb7
35	Got2	Ddost		Zc3h13			Ube2l3		Nav2
36	Tsfm	Nat10		Aff3			Aldh1a1		XLOC_027423

37	Hmga1	Got2	Thbs1	XLOC_041541
38	Prkcsh	Nop56	Ush2a	Mup2
39	Calr	Dhx9	Islr	Iigp1
40	Nolc1	Rpl14	Gm4070	2610528A11Rik
41	Dlat	Rps2	Veph1	Fam122b
42	gag-pol	Hells	Gvin1	Zc3h13
43	Actr5	Envelope protein	Npr3	Aff3
44	Rplp0	Hmga2	Ifi211	Thbs1
45	Zmpste24	Nolc1	Ifit1	Ush2a
46	Mdh2	Zmpste24	Ifi202b	Gm4070
47	Rps12	Pdia3	C3	Veph1
48	Dhx9	Atp5mf	Gbp2	Gm36107
49	Tmem176b	Ndufa13	Ly6c1	Npr3
50	Ddost	Pdia6	Plscr2	C3
51	Slc25a3	Pigs	Gpcpd1	Plscr2
52	gag-pro-pol	Lamb1	Sned1	Gpcpd1
53	Lamb1	Heatr1	Col18a1	Sned1
54	Ncl	Rps3	Capn6	Capn6
55	Snrpa1	Rab7	Ptn	Postn
56	Ssr3	H1-1	Postn	Trp53inp1
57	Hsd17b12	Nop2	Trp53inp1	Gm2895
58	Anxa2	Wdr36	Selenop	Selenop
59	Acot2	Degs1	Adamts5	AV356131
60	Hspa5	Ganab	Ndrg1	Ndrg1
61	Gfm1	Rrs1	Fhl2	
62	Ckap4	Parp1		
63	Lmna	Ndufa4		
64	Hadh	Calr		
65	Nap1l1	Mdh2		
66	Drg1	Ddx56		
67	Bcap31	Acot7		
68	Ddx47	Eif4a1		
69	Txndc12	Soat1		
70	Prdx3	Hspd1		
71	Ddx39a	Tmem176b		
72	Cbx3	Nup160		
73	Rpl30	gag-pol		
74	Slc25a4	Slc25a4		
75	Nip7	Mogs		
76	Pdcd11	Aldh18a1		
77	Parp1	Gfm1		
78	Ak2	Bud31		
79	Sqle	Cyp51a1		

80	Ganab	Ncl
81	Rpl23	Phb2
82	Hnrnpc	gag-pro-pol
83	Top2a	Myof
84	Fbl	Rps10
85	Mki67	Ergic1
86	Acot9	Snu13
87	Glud1	Smarca5
88	Nop2	Bop1
89	Wdr36	Pum3
90	Nxf1	Itgb1
91	Vdac2	Atp5f1b
92	Dlst	Ppan
93	Hsp90b1	Anxa5
94	Hnrnpa0	Sqle
95	Itgb1	Uxs1
96	Ddx56	Nop58
97		Slc25a11
98		Sf3b3
99		H1f0
100		Rps11
101		Rcc1
102		Tmpo
103		Fbl
104		Ddx18
105		Uggt1
106		Pes1
107		Cd109
108		Sirt2
109		Ckap4
110		Glud1
111		C1qbp
112		Macroh2a1
113		Dhx15
114		Hnrnpab
115		Ahnak
116		Acot9
117		Dlat
118		Gtpbp4
119		Acadl
120		Prdx3
121		Syncrip
122		Atp5f1a

123	Mmp14				
124	Grpel1				
125	Hnrnpa1				
126	Atp6v0c				
127	Ddx27				
128	Baz1b				
129	Atp2a2				
130	Mki67				
131	Nup205				
132	Gcsh				
133	Dad1				
134	Snrpa1				
135	Psap				
89	Trappc6a				
88	Eif4h				
87	Sec31a				
86	Cald1				
85	Srsf2				
84	Adh5				
83	Prdx6				
82	Park7				
81	Ppp2cb				
80	Eif5a				
79	Vwa5a				
78	Tln1				
77	Epb41l2				
76	Akr1a1				
75	Sh3bgrl3				
74	Arhgdia				
73	Pafah1b3				
72	Psme1				
71	Prrc2c				
70	Ywhaz				
69	Aldh9a1				
68	Fkbp5				
67	Phgdh				
66	Rnh1				
65	Psmc4				
64	Tpm3				
63	Acat2				
62	Sptbn1				
61	Prdx2				
60	Gsta4				Selenop

59	Tubb5	Sumo2							Plscr2
58	Fdps	Akr1b3							Serpinb6b
57	Mcm7	Ugp2				Cavin2			C3
56	Marcks	Mtpn				Adamts5			Dusp1
55	Plin4	Tkt				Selenop			Gbp9
54	Coro1b	Syap1				Cox8a			Fip1l1
53	S100a6	Ehd2				Plscr2			Postn
52	Mcm6	Psmc1				Serpinb6b			Capn6
51	Pkm	Stip1				C3			Tcp11l2
50	Lgals1	Pgam1				Trim30d			Pgm5
49	St3gal4	Tmsb10				Dusp1			Ccl9
48	Fabp5	Ldhb				Ptn			Dek
47	Endog	Eef1a1				Gbp9			Thbs1
46	Ccdc117	Cnn2				Fip1l1			Aff3
45	Nasp	Rac1				Postn			Gm4070
44	Gsn	Cstb				Gbp2			Dner
43	Gstm1	Ywhae				Ly6c1			Nfe2l1
42	Krt10	Blvrb				Ifi202b			Ak4
41	Ndufs1	Xdh				Ifit1	Add1		Veph1
40	Sptbn1	Ugdh				Capn6	Grn		Iigp1
39	Vcl	Hmgb1				Tcp11l2	Fbln5		Aldh1a1
38	Eef1a1	Pkm				Pgm5	Ufc1		Gm12153
37	Pcna	Pebp1				Ccl9	Srrm1		Fos
36	Idh1	Gsn				Dek	Itgb7		Adamts9
35	Spg20	Tagln2				Thbs1	Slc25a51		Afap1l2
34	Chaf1b	Cav1				Aff3	Cipc		XLOC_040479
33	Pgam1	Cbr2				Gvin1	Dnm3os		Itk
32	Cotl1	Gpi				Egr1	Kcnd3		XLOC_085411
31	Cbr2	Stmn1				Gm4070	XLOC_005000		Spaca9
30	Ldhb	Fscn1				Dner	Ppfia4		Gm30849
29	Aldoa	Fkbp1a				Aldh1a7	Art5	Pip5k1a	Gm48910
28	H1f0	Gapdh				Nfe2l1	Gm45645	Fip1l1	XLOC_053882
27	Mif	Gstm1				Ak4	Platr8	XLOC_074153	XLOC_087168
26	Pgk1	Ppia		Add1		Ogn	XLOC_160960	Fos	Ksr2
25	Mtap	Idh1		Grn		Veph1	Fam184b	Ube2v1	Gm11264
24	Rac1	Sod1	Tsfm	Akr1c18		Iigp1	Gm12506	A930012O16Rik	Cadm1
23	Gstm2	Cfl1	Tmsb10	Fbln5		Aldh1a1	Gm44955	Dapk1	Gm4724
22	Vwa5a	Pcna	Syap1	Ufc1		Fos	XLOC_084584	Gm45820	4930556G22Rik
21	Ndrg1	Uap1l1	Erh	Srrm1		Cdo1	Col25a1	8430426J06Rik	Dync1i1
20	Ftl1	Dbi	Cstb	Itgb7		Adamts9	XLOC_043432	Gm45645	XLOC_091837
19	Gm7298	Marcks	Rps20	Abl2	Pip5k1a	Cib2	XLOC_080942	Spaca9	XLOC_017596
18	Gsta4	Ndrg1	Ppia	Slc25a51	Fip1l1	Afap1l2	Gm28055	Gm14532	XLOC_053102
17	Blvrb	Coro1b	Cdc42	Cipc	Fos	Itk	XLOC_020660	XLOC_084584	XLOC_109768

16	Rpl13a	Vcl	Anxa5	Hist2h3c2	Prr11	Tmprss11e	2310043O21Rik	Col25a1	XLOC_086437
15	Gm20390	Carhsp1	Stip1	Rgn	Ube2v1	Cdx1	Csmd3	XLOC_024770	Gm42745
14	Lrrc20	Lgals1	Gsto1	Kcnd3	Dapk1	Spaca9	XLOC 066537	Ankfn1	Gm45901
13	Ywhae	Cavin1	Sod2	Ppfia4	Bfsp1	Ly6i	Gm49539	Csmd3	Gbp8
12	Tpi1	Mettl26	Dbi	Art5	Spaca9	Ksr2	Gm32786	XLOC 053102	Trpc5
11	Sh3bgrl3	Txn	Lgals1	Ankrd2	Col25a1	Ak9	Cacna2d3	XLOC_136572	XLOC_040327
10	Acy1	S100a4	Rps29	Yipf7	Tmprss11e	Cadm1	XLOC_065271	L3mbtl4	8430426J06Rik
9	Ehd2	Lrrc71	Pgk1	Gm4907	4930486L24Rik	Gm4724	XLOC_157103	Cacna2d3	XLOC_057817
8	Gpi	Nasp	Hmgb1	Fam184b	Gabrb1	Dync1i1	XLOC 061664	XLOC 002463	XLOC 026109
7	Gapdh	Lgals3	Txn	Col25a1	Ankfn1	Gm3488	Lrrc69	Lrrc69	Gm16685
6	Rpl15	Pgk1	Cfl1	Gabrb1	Csmd3	Scn7a	XLOC_078536	XLOC_161023	Bank1
5	Prdx1	Prdx1	Tpi1	Csmd3	L3mbtl4	Gbp8	Gm33696	XLOC_046129	XLOC_161023
4	Lrrc71	Aldoa	Aldoa	Cacna2d3	Cacna2d3	Pcp4l1	Gpc5	Gm33696	XLOC_127612
3	Cavin1	EG433182	EG433182	Lrrc69	Lrrc69	Magea5	4930401G09Rik	Gpc5	Gm38048
2	Rpl18	S100a6	S100a4	Gpc5	Gpc5	Trpc5	XLOC 029806	XLOC 124858	XLOC 031922
1	Rps9	Tpi1	Lgals3	Gm14327	Gm14327	Bank1	XLOC 161072	Gm38048	XLOC 159404
